# Supplementary material for: Novel Broccoli Sulforaphane-Based Analogues Inhibit the Progression of Pancreatic Cancer without Side Effects
Source: Biomolecules. 2020 May 15;10(5):769. doi: 10.3390/biom10050769 (PMC7277136; doi:10.3390/biom10050769)
Supplement: Supplementary file 1 [file biomolecules-10-00769-s001.zip › Biomolecules upload/Suppl_FigS2 Georgikou.pdf]

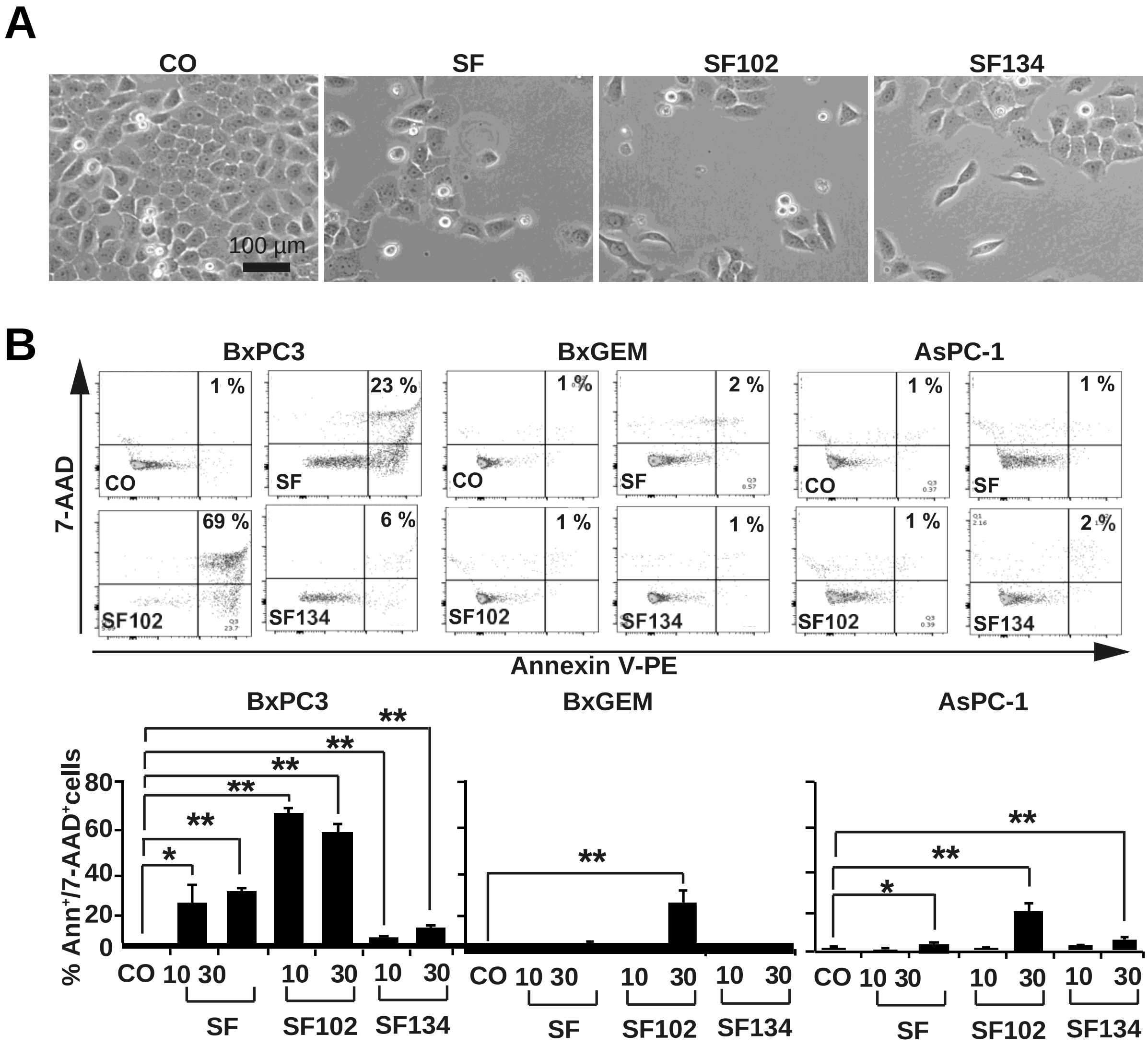

Figure S2 SF102 and SF134 induce apoptosis. (**A**) BxGEM cells were treated as described above. The cellular morphology was detected by microscopy and representative images are shown. The scale bar indicates 100 µm. (**B**) BxPc-3, BxGEM and AsPC-1 cells were treated with (SF), **SF102** or **SF134**, or were left untreated (CO). After 24 h, the cells were stained with Annexin V and 7-AAD and apoptosis was evaluated by flow cytometry, as previously described [1]. The percentage of double-stained cells ($Ann^+/7\text{-}AAD^+$) was detected and representative FACS dot blots (upper panel) along with the means ±SD are shown (lower panel). *$p < 0.05$, **$p < 0.01$.

Reference

[1] C. Georgikou, L. Yin, J. Gladkich, X. Xiao, C. Sticht, C. Torre, N. Gretz, W. Gross, M. Schafer, S. Karakhanova, I. Herr, Inhibition of miR30a-3p by sulforaphane enhances gap junction intercellular communication in pancreatic cancer, Cancer Lett., 469 (2020) 238-245.
