# Supplementary material for: Novel Broccoli Sulforaphane-Based Analogues Inhibit the Progression of Pancreatic Cancer without Side Effects
Source: Biomolecules. 2020 May 15;10(5):769. doi: 10.3390/biom10050769 (PMC7277136; doi:10.3390/biom10050769)
Supplement: Supplementary file 1 [file biomolecules-10-00769-s001.zip › Biomolecules upload/Suppl_FigS3 Georgikou.pdf]

Treatment with 10 µM sulfilimine SF101

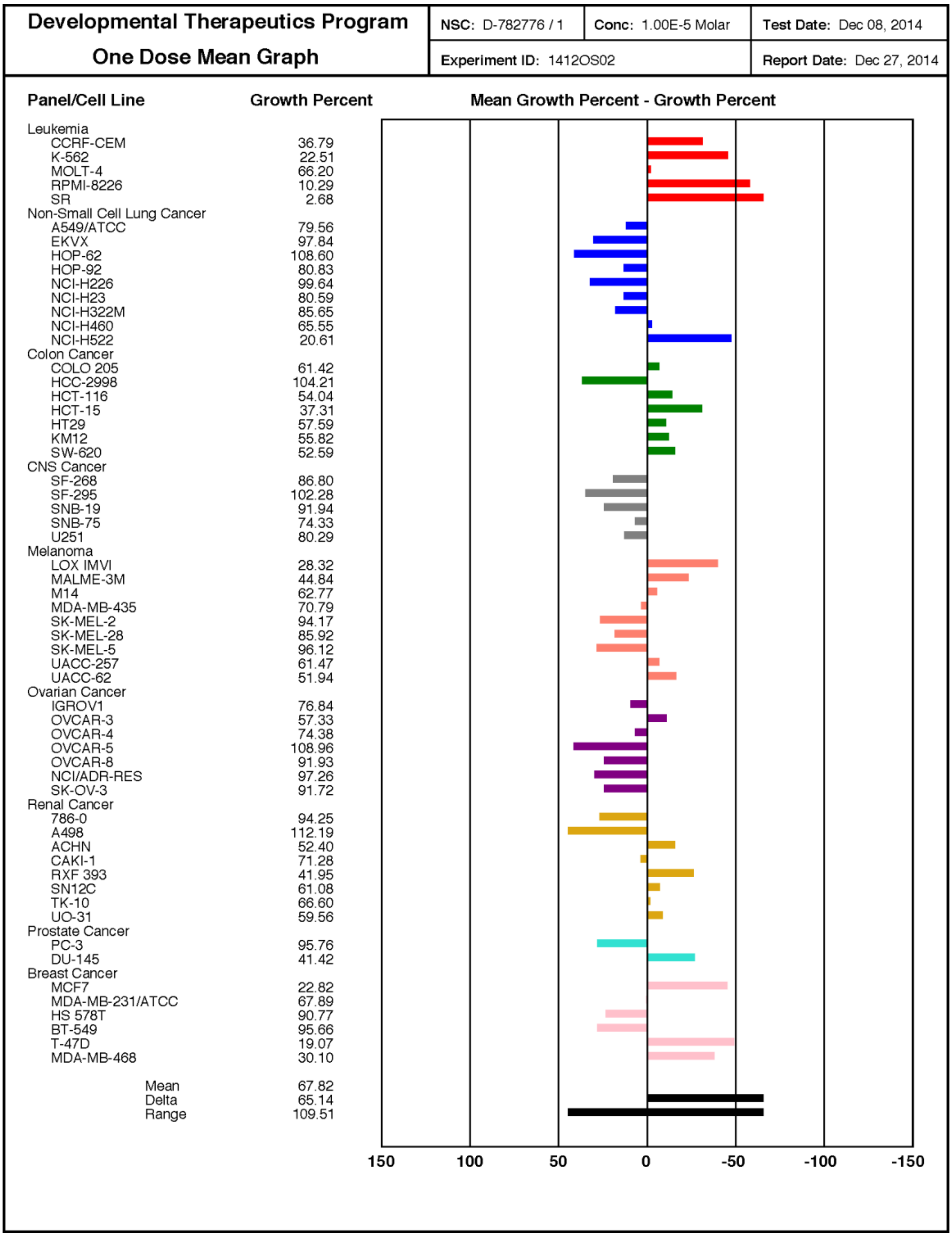

Figure S3 SF101 is effective in 60 cancer cell lines of the NCI-60 cell panel. These studies were performed by the Services of the Discovery & Developmental Therapeutics Program of the Division of Cancer Treatment and Diagnosis of the National Cancer Institute (NCI) at the National Institutes of Health, USA (http://dtp.cancer.gov). **SF101** was applied at a single dose (10 µM) to the 60 cancer cell lines representing leukemia, melanoma, non-small-cell lung carcinoma, and cancers of the brain, ovary, breast, colon, kidney, and prostate, as indicated. The growth inhibition (values between 0 and 100) and lethality (values less than 0) were measured. For example, a value of 100 means no growth inhibition. A value of 40 means 60% growth inhibition. A value of 0 means no net growth over the course of the experiment. A value of -40 means 40% lethality. A value of -100 means all cells are dead.
