# Supplementary material for: Novel Broccoli Sulforaphane-Based Analogues Inhibit the Progression of Pancreatic Cancer without Side Effects
Source: Biomolecules. 2020 May 15;10(5):769. doi: 10.3390/biom10050769 (PMC7277136; doi:10.3390/biom10050769)
Supplement: Supplementary file 1 [file biomolecules-10-00769-s001.zip › Biomolecules upload/Suppl_Information1 Georgikou.docx]

**SUPPORTING INFORMATION - CHEMISTRY**

**for**

**Sulforaphane analogs exhibit therapeutic activity in experimental pancreatic cancer**

**Christina Georgikoua#, Laura Buglionib#, Maximilian Bremerichb, Nico Roubicekb, Libo Yina, Wolfgang Grossa, Carsten Stichtc, Carsten Bolmb*, Ingrid Herra****

*aSection Surgical Research,Molecular OncoSurgery, Department of General, Visceral and Transplantation Surgery, University of Heidelberg, Heidelberg, Germany*

*bInstitute of Organic Chemistry, RWTH Aachen University, Aachen, Germany*

*cMedical Research Center, Medical Faculty Mannheim, University of Heidelberg, Mannheim, Germany*

* Corresponding author. Institute of Organic Chemistry, RWTH Aachen University, Aachen, Germany

** Corresponding author. Section Surgical Research,Molecular OncoSurgery, Department of General, Visceral and Transplantation Surgery, University of Heidelberg, Heidelberg, Germany

# Shared first authorship

*E-mail addresses*: Carsten.Bolm@oc.rwth-aachen.de (C. Bolm), i.herr@uni-heidelberg.de (I. Herr)

**Table of Content**

- General information (chemistry)

- Separation methods (chemistry)

- Instrumental analysis (chemistry)

- General procedures (chemistry)

- Characterization of the intermediates and products

- Fig. S3: Full NCI 60 cell panel for sulfilimine **SF101**

- Fig. S4: Full NCI 60 cell panel for sulfoximine **SF102**

- References

- NMR spectra

**General information (chemistry)**

Unless otherwise stated, all reagents and solvents were purchased from commercial suppliers and used without further purification. Dichloromethane (DCM), diethyl ether (Et2O), ethyl acetate (EtOAc) and pentane were purchased in technical quality and purified by distillation at atmospheric pressure before use. Reactions carried out at 0 °C or requiring removal of excess heat were cooled with an ice bath. Reactions at elevated temperature were carried out in heated silicone oil baths. All chiral products were prepared as racemates. The starting materials erucin (**1**), sulforaphane (**2**), and sulfide **4** were prepared as reported in the literature,S1 as well as the nitrene precursors **3**.S2 (4-Azidobutyl)(methyl)sulfane **12** was prepared from tetrahydrothiophene **10** according to a modified literature procedure.S3 Also the oxoimination of **12** followed a published protocol.S4 The two most active sulforaphane analogs **SF102** and **SF134** were independently prepared and purified by preparative HPLC (**SF134**) or standard flash chromatography (**SF102**). The purity was determined by analytic HPLC. **SF134** was delivered as one batch with ≥99.5% purity, **SF102** as three batches with 95.6%, 96.0% and 92.3% purity, respectively.

**Separation methods (chemistry)**

*Distillation*

Removal of solvents was carried out under reduced pressure (up to 10 mbar) on the rotary evaporator at 40 °C water bath temperature. Residual amounts of solvents were removed in a high vacuum (up to 0.05 mbar) at room temperature.

*Thin Layer Chromatography*

For TLC analysis, ALUGRAM® Xtra SIL G/UV254 TLC plates (0.2 mm silica gel, 5 cm length) from VWR were used. In addition to UV fluorescence quench detection (254 nm), various staining reagents were used. Aqueous potassium permanganate solution (0.4 g of KMnO4 in 200 mL of water) served as universal staining reagent. To stain isothiocyanates, an ethanolic solution of the silver diamine complex was used (0.1 g of AgNO3 in 5 mL of 25% aq. NH4OH and 20 mL of EtOH). Amines were stained with ethanolic ninhydrin solution (0.4 g of ninhydrin in 200 mL ethanol). Azides were first converted into their corresponding amines by dipping the plate into a solution of triphenylphosphine in DCM (0.1 g of PPh3 in 40 mL of DCM) and subsequent heating (heat gun), and then stained with ninhydrin solution. Azides could also be visualized with Ehrlich's reagent (1 g 4-*N,N*-dimethylaminobenzaldehyde in 75 mL MeOH with 25 mL conc. HCl).

*Column Chromatography*

Separations by flash column chromatography were performed with silica gel (grain size 35 μm to 60 μm) from Acros Organics as stationary phase (up to 0.4 bar overpressure).

**Instrumental analysis (chemistry)**

*NMR Spectroscopy*

All NMR spectra were recorded on a Varian-V-NMRs 600 spectrometer (1H: 600 MHz, 13C{1H}: 151 MHz, 19F: 564 MHz). Unless otherwise stated, CDCl3 was used as a solvent. Chemical shifts (δ) are given in ppm and are calibrated relative to the residual solvent signal. Coupling constants (J) are given in Hertz (Hz).

*Infrared Spectroscopy*

Infrared spectra were measured on a Perkin-Elmer Spectrum 100 FT-IR spectrometer.

*Mass Spectrometry*

Mass spectra were recorded on a Finnigan SSQ7000 spectrometer (electron impact ionization, 70 eV). High-resolution mass spectrometry was performed with a Thermo Scientific LTQ Orbitrap XL spectrometer (electrospray ionization).

*High Performance Liquid Chromatography*

Analytic and preparative HPLC was performed with a Varian PrepStar SD-1 solvent delivery system equipped with a Prostar 320 UV-vis detector (detection at 254 nm) and Kromasil-Si-100-40 cartridges.

**General procedures (chemistry)**

### *Light-mediated imidation procedure (GP1)*

To a solution of the sulfide or the sulfoxide (1.0 equiv.) and the corresponding 1,4,2-dioxazol-5-one (1.0 equiv.) **3a** or **3b** in dry toluene (4.0 mL/mmol) under argon, Ru(TPP)CO (1.0 mol%) was added, and the reaction was irradiated with a 125 W high-pressure mercury lamp at room temperature until full conversion was reached (never longer than 4 h). After evaporation of the solvent, the product was purified by column chromatography.

*Isothiocyanate formation procedure (GP2)*

The ammonium salt (1.0 equiv.) was dissolved in dry EtOH (4.0 mL/mmol) and Et3N (2.0 equiv.) was added. Then, CS2 (10 equiv.) was added dropwise, and the mixture was stirred for 1 hour at room temperature. Subsequently, Boc2O (1.0 equiv.) and DMAP (2 mol%) in dry EtOH (0.5 mL/mmol) were added at 0 °C. After stirring for further 5 minutes in an ice bath, the reaction mixture was kept at room temperature for 1 more hour. The solvent was evaporated, and the product was purified by column chromatography.

**Characterization of the intermediates and products**

**4-(Isothiocyanato)-1-(*N*-acetyl-*S*-methylsulfinimidoyl)butane** (**SF85**)

Following procedure GP1, starting from erucin **1** (40 mg, 0.25 mmol), the product was obtained after purification by column chromatography (EtOAc🡪EtOAc:EtOH 75:25) as a brownish oil (52 mg, 95% yield). 1H NMR (400 MHz, CDCl3)  3.59-3.56 (m, 2H), 3.01-2.98 (m, 1H), 2.89-2.86 (m, 1H), 2.61 (s, 3H), 2.01 (s, 3H), 1.88-1.81 (m, 4H); 13C{1H} NMR (600 MHz, CDCl3)  182.6, 131.3, 45.8, 44.5, 29.9, 28.8, 24.4, 20.6; IR (KBr) ν 2182, 2101, 1734, 1562, 1433, 1359, 1305, 980, 797 cm–1; MS (EI) *m/z* (%) 218 (10, [M]+), 159 (52), 113 (51), 72 (100), 63 (30), 55 (51); HRMS (*m/z*) [C8H14N2OS+H]+ Calcd. 219.0620, Found 219.0629.

**4-(Isothiocyanato)-1-(*N*-pentafluorobenzoyl-*S*-methylsulfinimidoyl)butane** (**SF101**)

Following procedure GP1, starting from erucin **1** (40 mg, 0.25 mmol), the product was obtained after purification by column chromatography (EtOAc🡪 EtOAc:EtOH 75:25) as a brownish oil (86 mg, 92% yield). 1H NMR (400 MHz, CDCl3)  3.63-3.59 (m, 2H), 3.11-3.09 (m, 2H), 2.78 (s, 3H), 1.98-1.89 (m, 4H); 13C{1H} NMR (100 MHz, CDCl3) δ 168.5, 144.0 (dm, *J* = 253.6 Hz), 140.3-139.7 (m), 137.5 (dm, *J* = 253.5 Hz), 131.6, 114.6 (t, *J* = 22.3 Hz), 46.1, 44.5, 30.0, 28.7, 20.5; 19F NMR (376 MHz, CDCl3)  –141.68 (dd, *J* = 22.1, 7.2 Hz, 2F), –154.77 (t, *J* = 20.7 Hz, 1F), –161.81 (dd, *J* = 20.9, 14.7 Hz, 2F); IR (KBr) ν 2186, 2102, 1584, 1496, 1417, 1337, 1263 1100, 985, 804 cm–1; MS (EI) *m/z* (%) 371 (10, [M+1]+), 195 (70), 160 (100), 114 (58), 72 (88), 55 (55); HRMS (*m/z*) [C13H11N2OS2F5+Na]+ Calcd. 393.0125, Found 393.0123.

**4-(Isothiocyanato)-1-(*N*-acetyl-*S*-methyl-sulfonimidoyl)-butane** (**SF86**)

Following procedure GP1, starting from sulforaphane **2** (44 mg, 0.25 mmol), the product was obtained after purification by column chromatography (EtOAc) as a brownish oil (38 mg, 62% yield). 1H NMR (600 MHz, CDCl3)  3.61 (t, *J* = 6.3 Hz, 2H), 3.53-3.48 (m, 1H), 3.38-3.33 (m, 1H), 3.24 (s, 3H), 2.11 (s, 3H), 2.01-1.98 (m, 2H), 1.90-1.87 (m, 2H); 13C{1H} NMR (150 MHz, CDCl3)  180.5, 131.8, 52.6, 44.6, 39.6, 28.6, 26.9, 19.6; IR (KBr) ν 2183, 2101, 1623, 1496, 1444, 1360, 1267 1207, 1036, 971, 832 cm–1; MS (EI) *m/z* (%) 234 (10, [M]+),113 (63), 114 (46), 72 (100), 55 (44); HRMS (*m/z*) [C8H14N2O2S2+Na]+ Calcd. 257.0388, Found 257.0399.

**4-(Isothiocyanato)-1-(*N*-pentafluorobenzoyl-*S*-methyl-sulfonimidoyl)-butane** (**SF102**)

Following procedure GP1, starting from sulforaphane **2** (44 mg, 0.25 mmol), the product was obtained after purification by column chromatography (EtOAc) as a brownish oil (73 mg, 75% yield). 1H NMR (600 MHz, CDCl3)  3.64-3.61 (m, 3H), 3.51-3.44 (m, 1H), 3.40 (s, H), 2.09-2.06 (m, 2H), 1.94-1.91 (m, 2H); 13C{1H} NMR (150 MHz, CDCl3)  166.1, 144.4 (dm, *J* = 253.0 Hz), 142.1 (dm, *J* = 256.5 Hz) 137.6 (dm, *J* = 253.5 Hz), 131.9, 114.0 (t, *J* = 18.5 Hz), 53.0, 44.5, 39.8, 28.4, 19.5; 19F NMR (376 MHz, CDCl3) δ –141.00 (dd, *J* = 23.6, 7.2 Hz, 2F), –152.30 (t, *J* = 20.8 Hz, 1F), –161.15 (dd, *J* = 20.9, 14.4, 2F); IR (KBr) ν 2188, 2105, 1738, 1625, 1494, 1330, 1249, 990, 761 cm–1; MS (CI, Methan) *m/z* (%) 387 (100, [M+1]+).

***tert*-Butyl [4-(*N*,*S*-dimethylsulfonimidoyl)butyl]carbamate** (**5**)

To a solution of MeNH2 (33% in EtOH, 1.5 mmol, 140.9 mg) in MeOH (6 mL/mmol), Br2 (1.04 mmol, 83.0 mg) was slowly added. After 5 min, sulfide **4** (115.0 mg, 0.52 mmol) was added, and the mixture stirred for further 12 min. Then, the solvent was evaporated and the mixture solubilized in acetone. The insoluble MeNH3Br salt was filtered off. To the filtrate, K2CO3 (1.04 mmol, 143.5 mg) and KMnO4 (1.6 mmol, 246.5 mg) were added. After 16 h, the mixture was washed with H2O (15.0 mL/mmol) and extracted with DCM (3x15.0 mL). The combined organic layers were dried over MgSO4 and the product purified by column chromatography (pentane:EtOAc 9:1🡪EtOAc:EtOH 3:1) as a colorless oil (42 mg, 30% yield). 1H NMR (600 MHz, CDCl3)  4.75 (s, 1H), 3.15-3.05 (m, 4H), 2.86 (s, 3H), 2.75 (s, 3H), 1.85-1.77 (m, 2H), 1.63-1.57 (m, 2H), 1.39 (s, 9H); 13C{1H} NMR (150 MHz, CDCl3)  156.1, 79.5, 53.4, 39.8, 38.6, 29.2, 29.1, 28.5, 20.8; IR (KBr) ν 2929, 1697, 1522, 1229, 1152, 997, 851, 759 cm–1; MS (EI) *m/z* (%) 265 (100, [M+1]+); HRMS (*m/z*) [C11H24N2O3S+Na]+ Calcd. 265.1580, Found 268.1579.

***tert*-Butyl [4-(*S*-methyl-*N*-cyano-sulfinimidoyl)butyl]carbamate** (**6**)

To a solution of sulfide **4** (200.0 mg, 0.9 mmol) and cyanamide (1.35 mmol, 56.7 mg) in CH3CN (3.0 mL/mmol), PhI(OAc)2 (0.99 mmol, 318.9 mg) was added at 0 °C, and the reaction was let stirring at room temperature over night. After evaporation of the solvent, the product was obtained after purification by column chromatography (EtOAc🡪EtOAc:EtOH 9:1) as a yellow oil (166 mg, 71% yield). 1H NMR (600 MHz, CDCl3)  4.88 (s, 1H), 3.13-3.09 (m, 3H), 2.99-2.96 (m, 1H), 2.75 (s, 3H), 1.85-1.78 (m, 2H), 1.66-1.61 (m, 2H), 1.38 (s, 9H); 13C{1H} NMR (150 MHz, CDCl3)  156.2, 120.3, 79.4, 49.0, 39.3, 32.7, 28.7, 28.4, 20.4; IR (KBr) ν 3332, 2948, 2141, 1704, 1514, 1374, 1164, 992, 764 cm–1; MS (EI) *m/z* (%) 296 (1, [M+1]+), 218 (100), 186 (40), 296 (1, [M+1]+); HRMS (*m/z*) [C11H21N3O2S+Na]+ Calcd. 282.1247, Found 282.1253.

***tert*-Butyl [4-(*S*-methyl-*N*-cyano-sulfonimidoyl)butyl]carbamate** (**7**)

To a stirred solution of the corresponding *N*-cyano sulfilimine **6** (160 mg, 0.60 mmol) in DCM (5.0 mL/mmol), were added K2CO3 (1.8 mmol, 248.8 mg) and *m*CPBA (0.9 mmol, 155.1 mg) at 0 °C. Then, the reaction was stirred at room temperature over night. The reaction mixture was washed with a sat. solution of NaHCO3 (5 mL) and extracted with DCM (3x3.0 mL). The combined organic layers were dried over MgSO4. The desired product was obtained after purification by column chromatography (EtOAc) as a colorless oil (100 mg, 59% yield). 1H NMR (400 MHz, CDCl3)  4.79 (s, 1H), 3.47-3.43 (m, 2H), 3.23 (s, 3H), 3.19-3.14 (m, 2H), 2.02-1.86 (m, 2H), 1.70-1.63 (m, 2H), 1.41 (s, 9H); 13C{1H} NMR (100 MHz, CDCl3)  156.5, 112.3, 79.7, 54.4, 40.3, 30.9, 28.7, 28.5, 19.5; IR (KBr) ν 3356, 2947, 2192, 1691, 1518, 1239, 1168, 980, 825 cm–1; MS (EI) *m/z* (%) 276 (1, [M+1]+), 220 (25), 202 (100), 176 (35), 175 (51); HRMS (*m/z*) [C11H21N3O3S+Na]+ Calcd. 298.1196, Found 298.1193.

***tert*-Butyl [4-(methylsulfinyl)butyl]carbamate** (**8**)

To a solution of sulfide **4** (280 mg, 1.3 mmol) in DCM (10.0 mL/mmol), a solution of *m*CPBA (77%, 1.1 equiv.) in DCM was added dropwise at 0 °C. Then, the mixture was stirred overnight. Subsequently, it was washed with a saturated solution of NaHCO3, extracted with DCM (3x20 mL/mmol) and dried over MgSO4. The combined organic layers were evaporated, and the reaction mixture was purified by column chromatography (EtOAc🡪EtOAc:EtOH 4:1), affording the desired product as a colorless oil (287 mg, 95% yield). 1H NMR (400 MHz, CDCl3)  4.71 (s, 1H), 3.14 (dd, *J* = 12.7, 6.3 Hz, 2H), 2.72-2.67 (m, 2H), 2.54 (s, 3H), 1.82-1.74 (m, 2H), 1.68-1.56 (m, 2H), 1.40 (s, 9H); 13C{1H} NMR (100 MHz, CDCl3)  156.1, 79.3, 54.0, 39.8, 38.6, 29.3, 28.5, 19.9; IR (KBr) ν 2973, 2931, 1695, 1525, 1364, 1249, 1167, 1023 cm–1; MS (EI) *m/z* (%) 236 (100, [M+1]+), 218 (54), 180 (68), 161 (66), 135 (86), 73 (44), 57 (95); HRMS (*m/z*) [C10H21NO3S+Na]+ Calcd. 258.1134, Found 258.1134.

***tert*-Butyl {4-[*S*-methyl-*N*-(2,2,2-trifluoroacetyl)sulfonimidoyl]butyl}carbamate** (**9**)

To a solution of the sulfoxide **8** (200 mg, 0.85 mmol), trifluoroacetamide (1.7 mmol, 192.2 g), MgO (3.4 mmol, 137.0 mg), and Rh2(OAc)4 (2.5 mol%, 9.4 mg) in DCM (5.0 mL/mmol), PhI(OAc)2 (1.3 mmol, 410.7 mg) was added at room temperature. The resulting mixture was stirred overnight. After evaporation of the solvent, the product was purified by column chromatography (pentane:EtOAc 1:1🡪EtOAc) as a white solid (190 mg, 65% yield). M.P. 115-116 °C; 1H NMR (600 MHz, CDCl3)  4.66 (s, 1H), 3.61-3.49 (m, 2H), 3.34 (s, 3H), 3.19 (dd, *J* = 12.5, 6.2 Hz, 2H), 1.95-1.88 (m, 2H), 1.69-1.64 (m, 2H), 1.43 (s, 9H); 13C{1H} NMR (150 MHz, CDCl3)  164.3 (q, *JC(O)CF3-F* = 38.1 Hz), 156.4, 116.0 (q, *JC-F* = 287.8 Hz), 79.7, 53.3, 39.2, 39.0, 28.9, 28.5, 19.1; 19F NMR (376 MHz, CDCl3) δ –75.91 (s, 3F); IR (KBr) ν 1671, 1529, 1378, 1159, 983, 831, 761 cm–1; MS (EI) *m/z* (%) 347 (1, [M+1]+), 273 (30), 166 (100), 116 (44), 98 (34), 57 (65); HRMS (*m/z*) [C12H21N2O4SF3+Na]+ Calcd. 369.4066, Found 369.4077.

**4-(Isothiocyanato)-1-(*N*-methyl-*S*-methylsulfonimidoyl)butane** (**SF113**)

To a solution of the carbamate **5** (125 mg, 0.47 mmol), in dry DCM (3.0 mL/mmol), 4m HCl in dioxane (1.41 mmol, 0.35 mL) was added and the mixture was stirred overnight. After evaporation, the hydrochloridric salt was obtained as a white solid in quantitative yield and it was used without further purification. After following GP2, **SF113** was obtained after purification by column chromatography (EtOAc🡪EtOAc:EtOH 9:1) as a yellow oil (16 mg, 75% yield). 1H NMR (400 MHz, –20 °C, CDCl3)  3.61 (t, *J* = 5.8 Hz, 2H), 3.17-3.08 (m, 2H), 2.95 (s, 3H), 2.78 (s, 3H), 1.95-1.91 (m, 2H), 1.85-1.83 (m, 2H); 13C{1H} NMR (100 MHz, –20 °C, CDCl3)  129.5, 52.6, 44.5, 38.3, 29.2, 28.5, 20.8; IR (KBr) ν 2929, 2181, 2102, 1344, 1227, 1132, 848, 733, 630, 543 cm–1; MS (EI) *m/z* (%) 207 (88, [M+1]+), 206 (34, [M]+), 148 (78), 114 (49), 93 (64), 78 (81), 72 (100), 55 (68); HRMS (*m/z*) [C7H14N2O4S2+H]+ Calcd. 207.0620, Found 207.0613.

**4-(Isothiocyanato)-1-[*N*-(2,2,2-trifluoroacetyl)-*S*-methyl-sulfonimidoyl]butane** (**SF134**)

To a solution of the carbamate **9** (178 mg, 0.50 mmol) in dry DCM (3.0 mL/mmol), 4m HCl in dioxane (1.5 mmol, 0.37 mL) was added and the mixture was stirred overnight. After evaporation, the hydrochloridric salt was obtained as a white solid in quantitative yield, and it was used without further purification. After following GP2, **SF134** was obtained after purification by column chromatography (pentane:EtOAc 1:1) as a yellow oil (72 mg, 50% yield

**4-(Isothiocyanato)-1-(*N*-carbamoyl-*S*-methyl-sulfonimidoyl)butane** (**SF135**)

To a solution of carbamate **7** (54 mg, 0.2 mmol) in DCM (10 mL/mmol), trifluoroacetic acid (2.6 mmol, 296.4 mg) and Et3SiH (0.5 mmol, 58.1 mg) were added. The solution was stirred at room temperature for 16 h. After evaporation, the hydrochloridric salt was obtained as a white solid in quantitative yield, and it was used without further purification. Following GP2, **SF135** was obtained after purification by column chromatography (EtOAc🡪EtOAc:EtOH 9:1) as a colorless oil (47 mg, 83% yield). 1H NMR (600 MHz, CDCl3)  5.07 (s, 2H), 3.61 (t, *J* = 6.3 Hz, 2H), 3.53-3.47 (m, 1H), 3.40-3.32 (m, 1H), 3.24 (s, 3H), 2.03-1.96 (m, 2H), 1.92-1.84 (m, 2H); 13C{1H} NMR (150 MHz, CDCl3)  161.8, 131.5, 53.0, 44.5, 40.0, 28.5, 19.8; IR (KBr) *ν* 3344, 2101, 1630, 1371, 1196, 1119 cm–1; MS (EI) *m/z* (%) 235 (41, [M]+), 122 (53), 107 (56), 72 (100), 58 (45), 46 (28); HRMS (*m/z*) [C7H13N3O2S2+Na]+ Calcd. 258.0.341, Found 258.0341.

**Streamlined process for the preparation of SF102 and SF134**

### 1-Methyltetrahydro-1H-thiophen-1-ium tetrafluoroborate (11)

A 500 mL-round-bottom flask was charged with tetrahydrothiophene (**10**, 20.0 g, 227 mmol) and cooled in an ice bath. Methyl iodide (32.2 g, 14.1 mL, 227 mmol, 1.00 equiv.) was added, and the mixture was kept on ice for 5 more minutes before being placed in an ultrasonic bath at 50 °C for 1 h. The mixture was then cooled in an ice bath for another 5 minutes. The resulting yellow solid was dissolved in ethanol (150 mL), and a suspension of sodium tetrafluoroborate (24.9 g, 227 mmol, 1.00 equiv.) in ethanol (150 mL) was added. The suspension was heated to reflux for 30 min, and the hot supernatant liquid was decanted off. The liquid was allowed to cool down in the fridge, and the precipitated crystals were washed with a small amount of cold ethanol. Recrystallization (twice) from ethanol and drying under reduced pressure furnished the desired product as colorless crystalline solid (36.2 g, 191 mmol, 84%).

**Note:** It should be taken care that the product is of high purity, since traces of **10** in the product will lead to the formation of tetrahydrothiophene sulfoximine derivatives in the later stages of the synthesis, which are very hard to remove, while traces of 1-methyltetrahydro-1H-thiophen-1-ium iodide can lead to the formation of highly toxic and explosive methyl azide in the next step.

1H NMR (400 MHz, D2O):  = 3.51 – 3.39 (m, 2H), 3.26 – 3.15 (m, 2H), 2.64 (s, 3H), 2.32 – 2.19 (m, 2H), 2.19 – 2.06 (m, 2H) ppm. 13C{1H} NMR (101 MHz, D2O):  = 44.7, 27.9, 24.9 ppm. MS (EI): *m/z* (%) = 101 (100, [M-2H]+), 87 (38).

**(4-Azidobutyl)(methyl)sulfane (12)**

1-Methyltetrahydro-1H-thiophene-1-ium tetrafluoroborate (**11**, 6.55 g, 34.5 mmol) was added to a suspension of sodium azide (6.72 g, 104 mmol, 3.00 equiv.) in DMF (70 mL). The mixture was heated to 80 °C for 16 h. After cooling down to room temperature, remaining sodium azide was dissolved by adding water (130 mL). The solution was extracted with pentane (3 x 70 mL). The combined organic phases were washed with water (2 x 50 mL) and brine (50 mL) and dried over anhydrous sodium sulfate. Removal of the solvent under reduced pressure (>200 mbar, lower pressure led to substance loss) furnishes the desired product as colorless liquid with intense odor (4.5 g, 31 mmol, 90%).

**Note:** For safety reasons, no experiments were carried out which would have led to more than 5 g of the desired product (full conversion assumed). The solvent distillation was carried out behind a blast shield, but no explosive or otherwise unexpected behavior of the azide **11** was observed.

1H NMR (600 MHz, CDCl3):  = 3.30 (t, *J* = 6.3 Hz, 2H), 2.52 (t, *J* = 6.7 Hz, 2H), 2.10 (s, 3H), 1.75 – 1.64 (m, 4H) ppm. 13C{1H} NMR (151 MHz, CDCl3):  = 51.0, 33.7, 27.9, 26.1, 15.5 ppm. MS (EI): *m/z* (%) = 146 [M+H]+ (12), 118 (30), 117 (72), 103 (100), 91 (17), 89 (52), 70 (33), 61 (18). IR (ATR): *ν* = 3324, 2920, 2864, 2091, 1434, 1349, 1264, 1072, 1001, 959, 902, 755 cm–1. TLC: Rf (silica, pentane/EtOAc 97:3) = 0.39.

### (4-Azidobutyl)(imino)(methyl)-λ6-sulfanone (13)

(4-Azidobutyl)(methyl)sulfane (**12**, 3.52 g, 24.2 mmol, 1.00 equiv.) was dissolved in methanol (120 mL), and the solution was cooled in an ice bath. After adding ammonium carbamate (7.56 g, 96.8 mmol, 4.00 equiv.), PIDA (19.5 g, 60.5 mmol, 2.50 equiv.) was added slowly and in portions to the solution. The mixture was stirred for 90 min at room temperature. Removal of the solvent under reduced pressure and purification by flash column chromatography (silica, DCM/EtOH 9:1), furnished the desired product as colorless liquid (2.94 g, 16.7 mmol, 69%).

1H NMR (600 MHz, CDCl3):  = 3.37 (t, *J* = 6.6 Hz, 2H), 3.16 – 3.10 (m, 2H), 3.01 (s, 3H), 2.06 (s, 1H), 2.00 – 1.91 (m, 2H), 1.78 – 1.71 (m, 2H) ppm. 13C{1H} NMR (151 MHz, CDCl3):  = 56.4, 50.9, 43.1, 27.8, 20.6 ppm. MS (EI): *m/z* (%) = 177 [M+H]+ (33), 134 (100), 93 (40), 80 (40), 79 (99), 78 (32), 77 (17), 64 (82), 63 (34), 60 (26), 55 (70), 47 (19), 46 (23). HRMS: [M+H]+ = [C5H13N4OS]+, calcd. 177.0805, found 177.0804. IR (ATR): *ν* = 3828, 3484, 3272, 2936, 2327, 2097, 1867, 1712, 1453, 1415, 1367, 1262, 1204, 1009, 876, 810, 745 cm–1. TLC: Rf (silica, DCM/EtOH 9:1) = 0.34.

### *N*-[(4-Azidobutyl)(methyl)(oxo)-λ6-sulfaneylidene]-2,2,2-trifluoroacetamide (14)

(4-Azidobutyl)(imino)(methyl)-λ6-sulfanone **(13**, 2.21 g, 12.5 mmol, 1.00 equiv.) was dissolved in DCM (60 mL), and 4-DMAP (153 mg, 1.25 mmol, 0.10 equiv.), trifluoroacetic anhydride (3.16 g, 15.0 mmol, 1.20 equiv.) and triethylamine (1.65 g, 16.3 mmol, 1.30 equiv.) were added. The mixture was stirred for 16 h at room temperature and then washed with 1 M HCl (15 mL), water (2 x 15 mL) and brine (15 mL) and dried over anhydrous sodium sulfate. After removal of the solvent under reduced pressure, the crude product was purified by flash column chromatography (silica, pentane/acetone 3:1) and obtained as brown oil (2.32 g, 8.52 mmol, 68%).

1H NMR (600 MHz, CDCl3):  = 3.56 (ddd, J = 14.0, 9.6, 6.2 Hz, 1H), 3.48 – 3.43 (m, 1H), 3.41 (t, J = 6.4 Hz, 2H), 3.36 (s, 3H), 2.06 – 1.94 (m, 2H), 1.79 (tq, J = 8.8, 6.5, 5.9 Hz, 2H) ppm. 13C{1H} NMR (151 MHz, CDCl3):  = 179.3, 164.1 (q, J = 43.6, 42.1, 38.6 Hz), 53.2, 50.5, 39.2, 27.3, 19.4 ppm. 19F NMR (564 MHz, CDCl3):  = –75.9 (s, 3F) ppm. MS (EI): *m/z* (%) = 273 [M+H]+ (89), 203 (83), 148 (31), 106 (19), 105 (26), 70 (100), 69 (18), 68 (21), 63 (19), 55 (16). HRMS (ESI): [M+Na]+ = [C7H11O2N4F3SNa]+, calcd. 295.0445, found 295.0447. IR (ATR): *ν* = 3023, 2936, 2880, 2101, 1667, 1458, 1381, 1313, 1175, 1083, 987, 901, 832, 776, 728 cm–1. TLC: Rf (pentane/acetone 3:1) = 0.36.

**4-(Isothiocyanato)-1-[*N*-(2,2,2-trifluoroacetyl)-*S*-methyl-sulfonimidoyl]butane (SF134)**

Tributyl phosphine (1.93 g, 9.53 mmol, 1.10 equiv.) was added to a solution of *N*-[(4-Azidobutyl)(methyl)(oxo)-λ6-sulfaneylidene]-2,2,2-trifluoroacetamide **14** (2.36 g, 8.66 mmol, 1.00 equiv.) in THF (45 mL) and stirred for 30 min at room temperature. Triethylamine (3.07 g, 4.23 mL, 30.3 mmol, 3.50 equiv.) and CS2 (1.98 g, 1.57 mL, 26.0 mmol, 3.00 equiv.) were added under ice cooling. After stirring at room temperature for 90 min, mesyl chloride (1.19 g, 0.805 mL, 10.4 mmol, 1.20 equiv.) was added under ice cooling, and the yellow solution was stirred for further 40 min at room temperature. The solution was diluted with Et2O (200 mL) and washed with 1 M HCl (50 mL), water (2 x 50 mL), brine (50 mL) and then dried over anhydrous sodium sulfate. The solvent was removed under reduced pressure and the crude product was purified by flash column chromatography (silica, Et2O). Yellowish oil (905 mg, 3.14 mmol, 36%).

**1H NMR** (600 MHz, CDCl3): δ = 3.63 (t, *J* = 6.3 Hz, 3H), 3.56 (ddd, *J* = 14.0, 9.2, 6.4 Hz, 1H), 3.45 (ddd, *J* = 14.1, 9.1, 6.3 Hz, 1H), 3.38 (s, 3H), 2.08 – 2.00 (m, 2H), 1.95 – 1.86 (m, 2H) ppm.

***N*-[(4-Azidobutyl)(methyl)(oxo)-λ6-sulfaneylidene]-2,3,4,5,6-pentafluoro­benz­amide (15)**

Perfluorobenzoic acid (4.33 g, 20.4 mmol, 1.50 equiv.) was dissolved in thionyl chloride (40 mL) and heated to reflux for 16 h under argon. After cooling down, excess thionyl chloride was removed under reduced pressure (40 mbar). The crude perfluorobenzoyl chloride was added to a solution of (4-azidobutyl)(imino)(methyl)-λ6-sulfanone(**13**, 2.60 g, 14.8 mmol, 1.00 equiv.) and triethylamine (2.24 g, 3.08 mL, 22.1 mmol, 1.50 equiv.) in DCM (50 mL) under argon. After stirring for 16 h at room temperature, the solvent was removed under reduced pressure. Purification by flash column chromatography (silica, Et2O) afforded the desired product as brown oil (4.04 g, 11.0 mmol, 74%).

1H NMR (600 MHz, CDCl3):  = 3.58 (dt, *J* = 13.9, 7.9 Hz, 1H), 3.47 (dt, *J* = 13.9, 7.9 Hz, 1H), 3.39 (t, *J* = 6.5 Hz, 2H), 3.37 (s, 3H), 2.01 (tt, *J* = 7.9, 6.3 Hz, 2H), 1.81 – 1.74 (m, 2H) ppm. 13C{1H} NMR (151 MHz, CDCl3):  = 165.9, 145.1 (td, J = 7.7, 3.9 Hz), 143.4 (td, J = 7.7, 3.9 Hz), 142.7 (d, J = 13.6 Hz), 141.0 (d, J = 13.7 Hz), 138.8 – 137.9 (m), 136.6 (dd, J = 17.4, 11.8 Hz), 114.0 (t, J = 17.4 Hz), 53.2, 50.6, 39.5, 27.3, 19.4 ppm. 19F NMR (564 MHz, CDCl3):  = –140.9 – –141.4 (m, 2F), –152.6 (tt, *J* = 20.6, 2.8 Hz, 1F), –161.0 – –161.6 (m, 2F) ppm. MS (EI): *m/z* (%) = 371 [M+H]+ (49), 258 (16), 195 (100), 70 (27). HRMS: [M+H]+ = [C12H12F5N4O2S]+, calcd. 371.0596, found. 371.0598. IR (ATR): *ν* = 3023, 2963, 2879, 2098, 1624, 1493, 1413, 1392, 1250, 1110, 1078, 990, 849, 815, 762 cm–1. TLC: Rf  (silica, Et2O) = 0.42.

**4-(Isothiocyanato)-1-(*N*-pentafluorobenzoyl-*S*-methyl-sulfonimidoyl)-butane** (**SF102**)

Zinc (5.81 g, 88.8 mmol, 10.0 equiv.) and ammonium chloride (1.18 g, 22.0 mmol, 2.50 equiv.) were added to a solution of *N*-[(4-azidobutyl)(methyl)(oxo)-λ6-sulfaneylidene]-2,3,4,5,6-pentafluo-robenzamide (**15,** 3.29 g, 8.88 mmol, 1.0 equiv.) in methanol (30 mL) and stirred at room temperature. After 40 min, the solution was filtered (paper), the solvent was removed under reduced pressure, and the residue was taken up in THF (20 mL). Triethylamine (4.03 g, 5.53 mL, 39.9 mmol, 4.50 equiv.) and CS2 (1.01 g, 0.805 mL, 13.3 mmol, 1.50 equiv.) were added under ice cooling, and the solution was stirred at room temperature. After 90 min, mesyl chloride (1.12 g, 0.753 mL, 9.75 mmol, 1.10 equiv.) was added under ice cooling, and the solution was stirred for another 40 min at room temperature. The solvent was removed under reduced pressure, the residue redissolved in Et2O (75 mL), and the organic layer was washed with 1 M HCl (20 mL), water (2 x 20 mL) and brine (20 mL). After drying over anhydrous sodium sulfate and removal of the solvent under reduced, the crude product was purified by a sequence of flash column chromatographies (1.: Et2O -> Et2O/acetone 9:1; 2.: DCM/acetone 99:1; 3.: Et2O/EtOAc 95:5). The desired product was obtained as yellowish oil (1.49 g, 3.82 mmol, 43%).

1H NMR (600 MHz, CDCl3):  = 3.63 (t, *J* = 6.3 Hz, 2H), 3.48 (dt, *J* = 13.8, 7.7 Hz, 1H), 3.39 (s, 3H), 2.11 – 2.01 (m, 2H), 1.97 – 1.87 (m, 2H) ppm. 13C{1H} NMR (151 MHz, CDCl3):  = 165.9, 145.1 (ddd, *J* = 11.5, 7.6, 3.9 Hz), 143.8 – 143.1 (m), 143.0 – 142.3 (m), 141.4 – 140.6 (m), 138.6 – 137.9 (m), 136.6 (t, *J* = 14.8 Hz), 131.6, 113.9 (t, *J* = 18.7 Hz), 52.8, 44.4, 39.7, 28.3, 19.4 ppm. 19F NMR (564 MHz, CDCl3):  = –140.7 – –141.2 (m, 2F), –152.3 (tt, *J* = 20.5, 2.9 Hz, 1F), –161.0 – –161.4 (m, 2F). MS (EI): *m/z* (%) = 195 (88), 167 (38), 117 (23), 72 (100), 55 (24). HRMS (ESI): [M+Na]+ = [C13H11F5N2O2S2Na]+, calcd. 409.0074, found 409.0072. IR (ATR): *ν* = 3020, 2933, 2879, 2347, 2188, 2104, 1624, 1493, 1412, 1329, 1251, 1110, 1073, 991, 850, 814, 762, 687 cm–1. HPLC: Rt (*n*-hexane/*iso*-propanol 85/15, 32 bar, 40 mL/min) = 43.3 min. TLC: Rf (silica, Et2O/EtOAc 95:5) = 0.23.

**References**

S1. a) J. R. Mays, R. L. Weller Roska, S. Sarfaraz, H. Mukhtar, S. R. Rajski, *ChemBioChem* **2008**, *9*, 729-747; b) X. Chen, Z. Li, X. Sun, H. Ma, X. Chen, J. Ren, K. Hu, *Synthesis* **2011**, 3991-3996; c) P. Kiełbasiński, J. Łuczak, T. Cierpiał, J. Błaszczyk, L. Sieroń, K. Wiktorska, K. Lubelska, M. Milczarek, Z. Chilmończyk, *Eur. J. Med. Chem.* **2014**, *76*, 332-342.

S2. a) V. Bizet, L. Buglioni, C. Bolm, *Angew. Chem. Int. Ed.* **2014**, *53*, 5639-5642; b) V. Bizet, C. Bolm, *Eur. J. Org. Chem.* **2015**, 2854-2860.

S3. D. V. Vo, V. D. Truong, T. D. Tran, V. T. N. Do, N. T. A Pham, K. M. Thai, *Lett. Org. Chem.* **2016**, *13*, 7-10.

S4. M. Zenzola, R. Doran, L. Degennaro, R. Luisi, J. A. Bull, *Angew. Chem. Int. Ed.* **2016**, *55*, 7203-7207.

**NMR spectra**


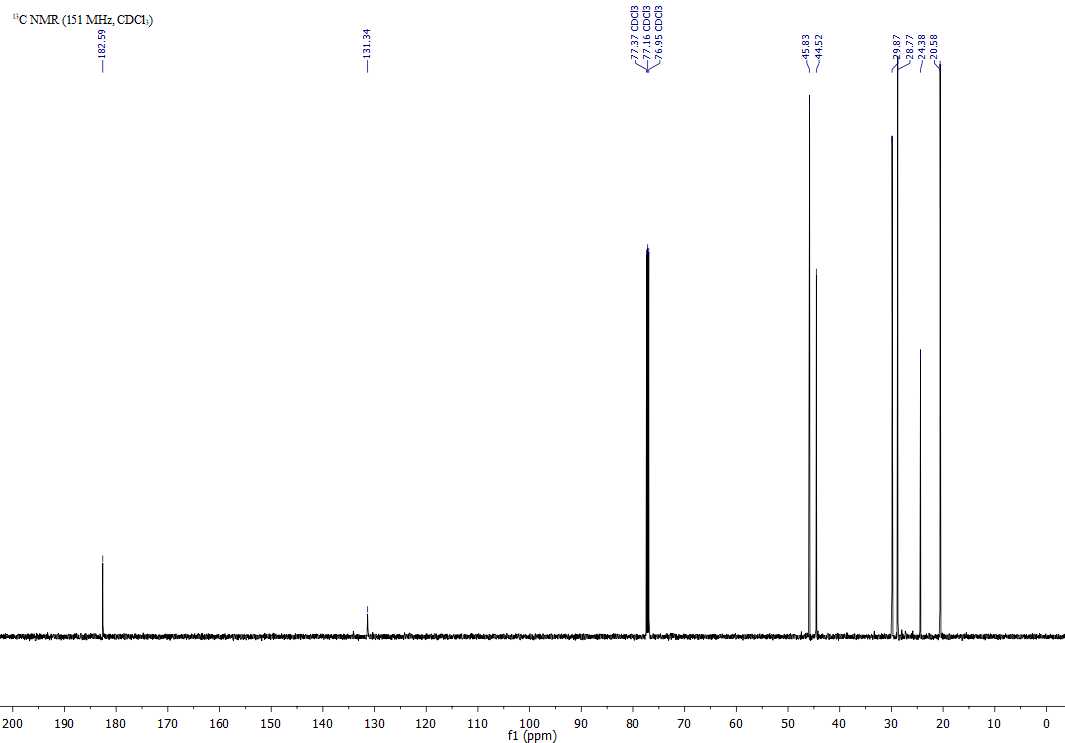
**4-(Isothiocyanato)-1-(*N*-acetyl-*S*-methylsulfinimidoyl)butane (SF85)**


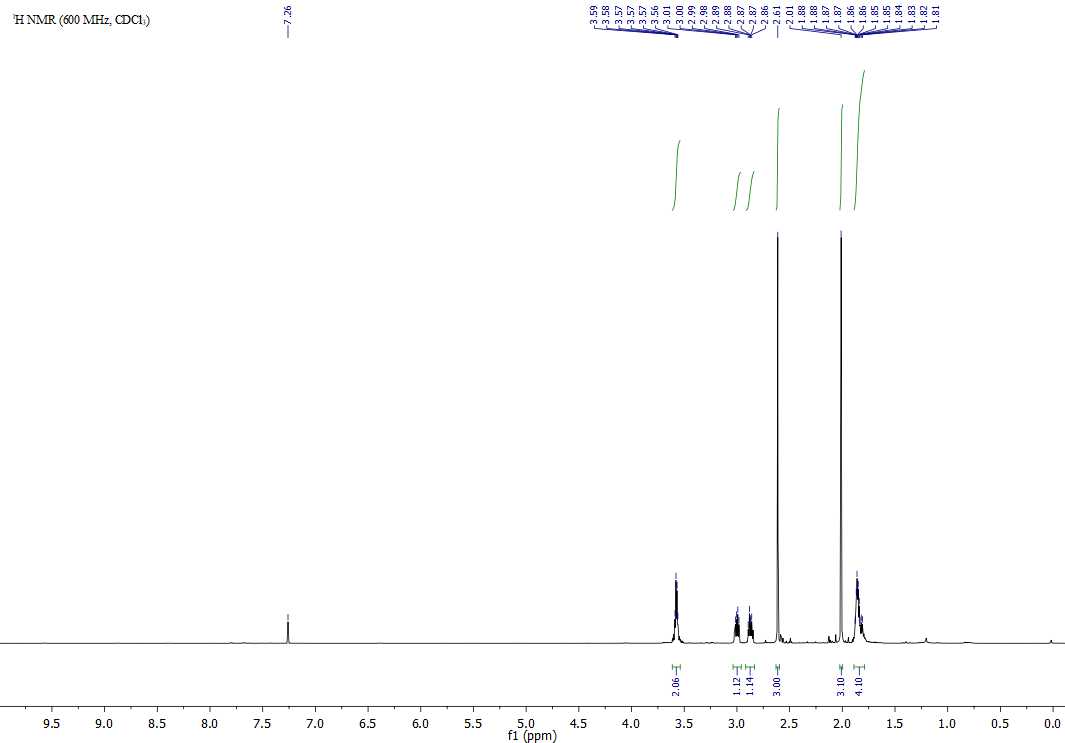


**4-(Isothiocyanato)-1-(*N*-pentafluorobenzoyl-*S*-methylsulfinimidoyl)butane (SF101)**


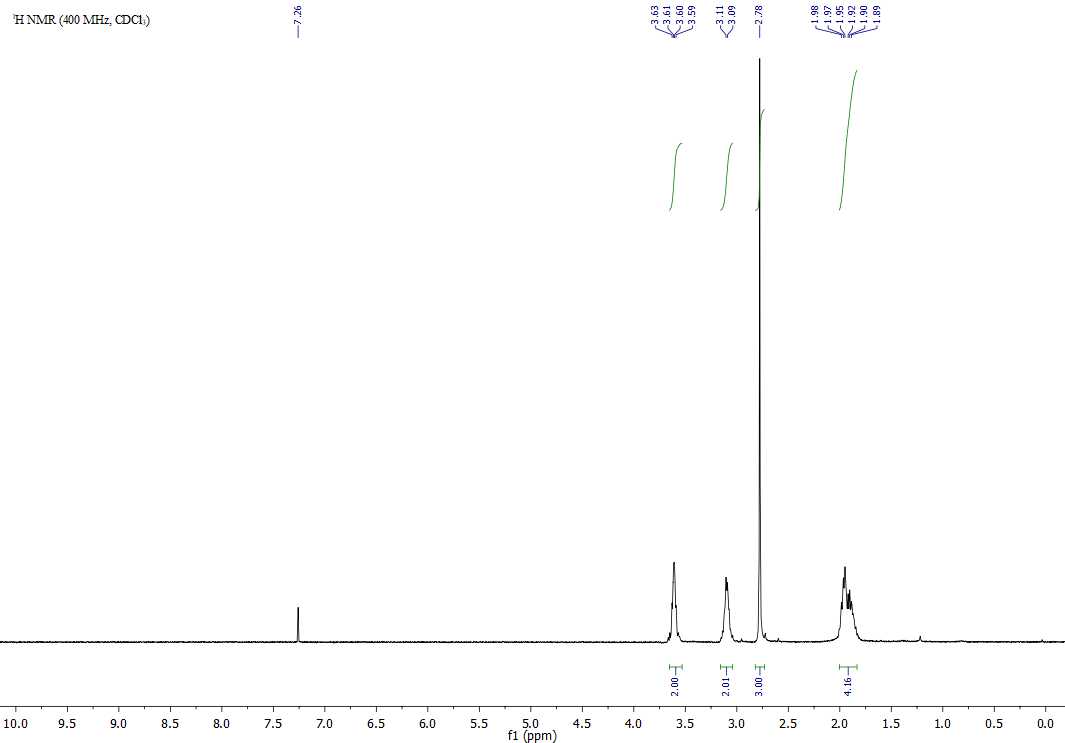


**
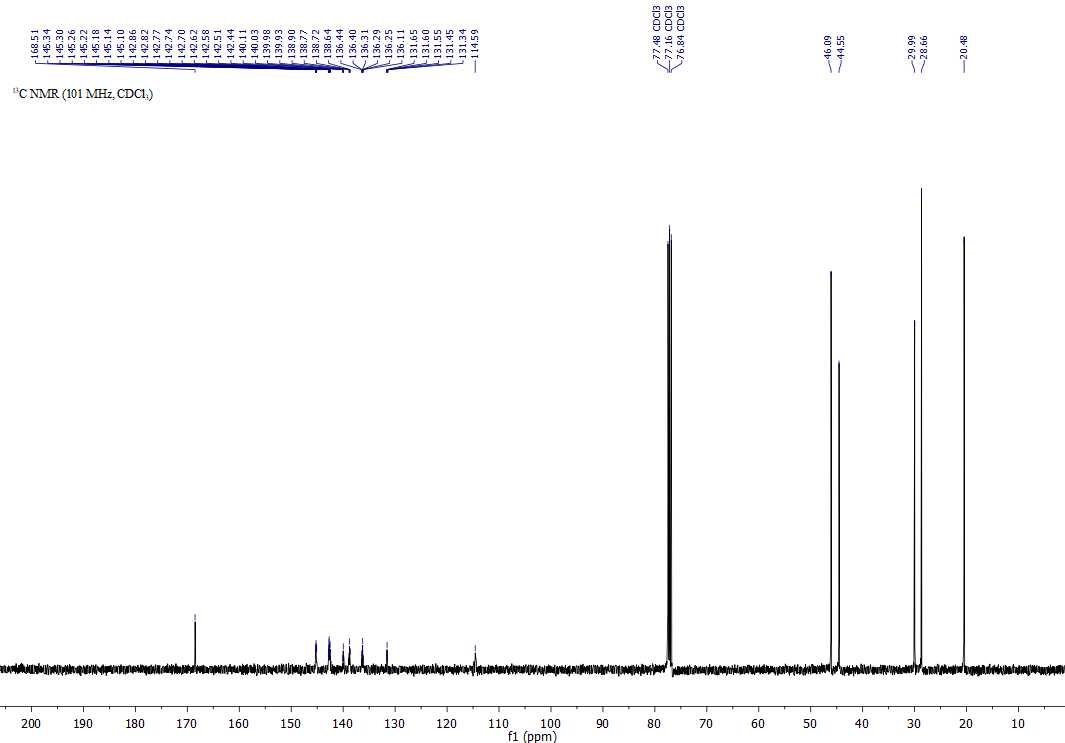
**

**
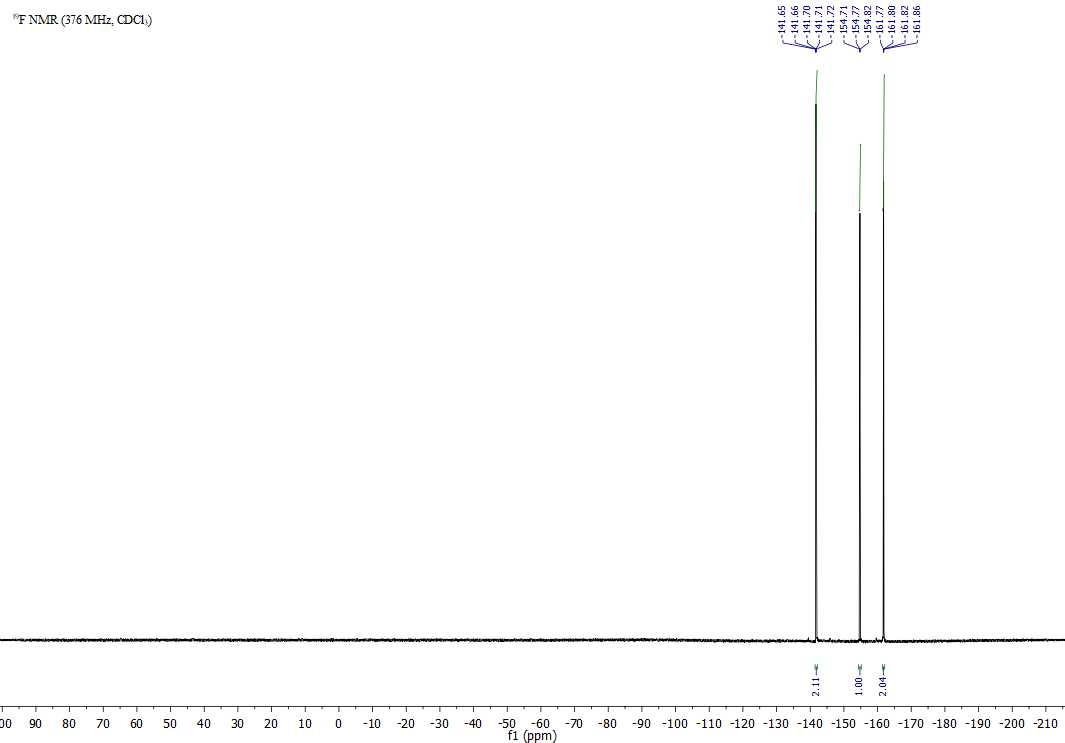
**

**4-(Isothiocyanato)-1-(*N*-acetyl-*S*-methyl-sulfonimidoyl)-butane (SF86)**


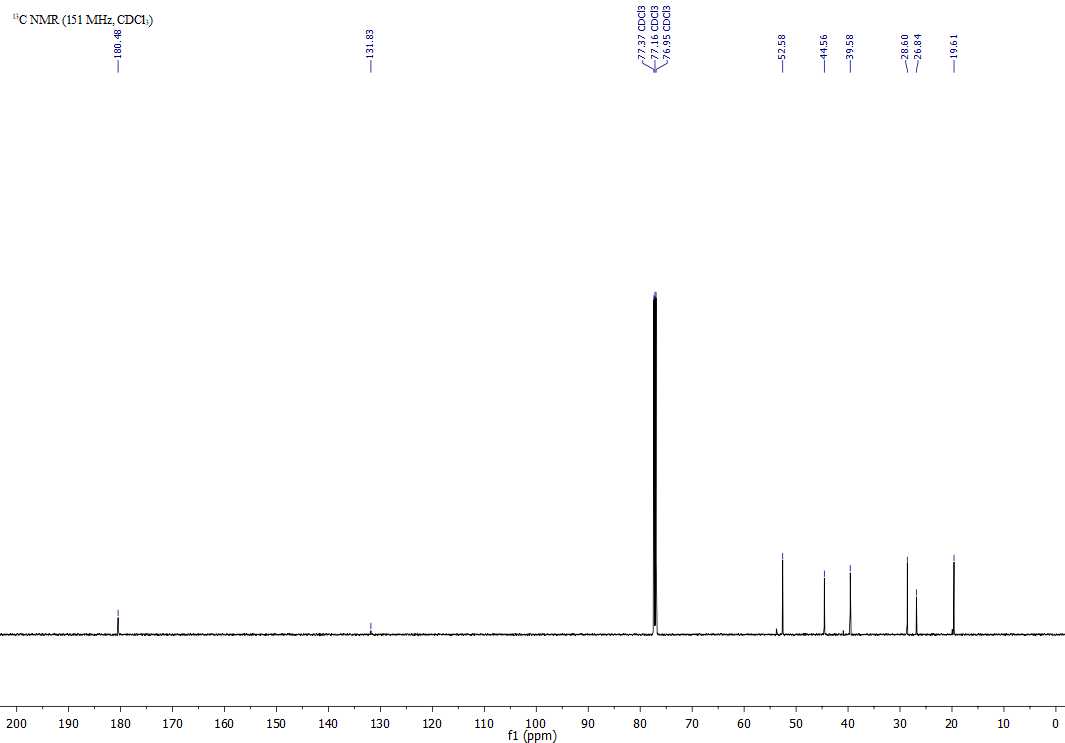

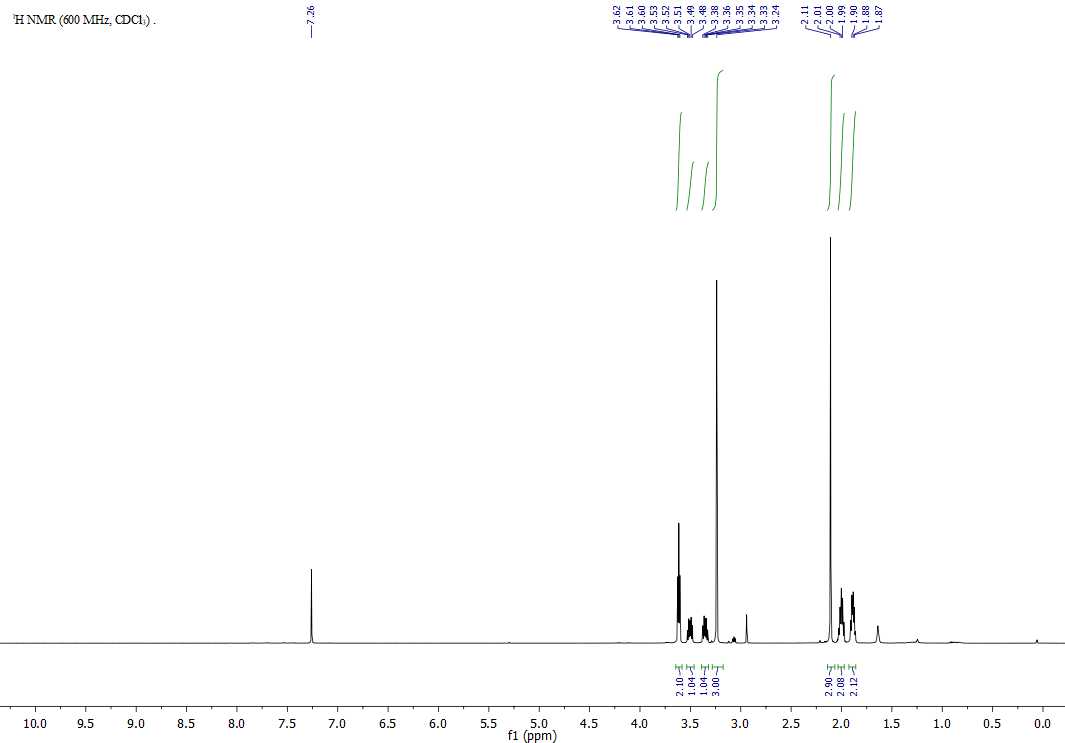


**4-(Isothiocyanato)-1-(*N*-pentafluorobenzoyl-*S*-methyl-sulfonimidoyl)-butane (SF102) - prepared by GP1 as described in Scheme 1**

**
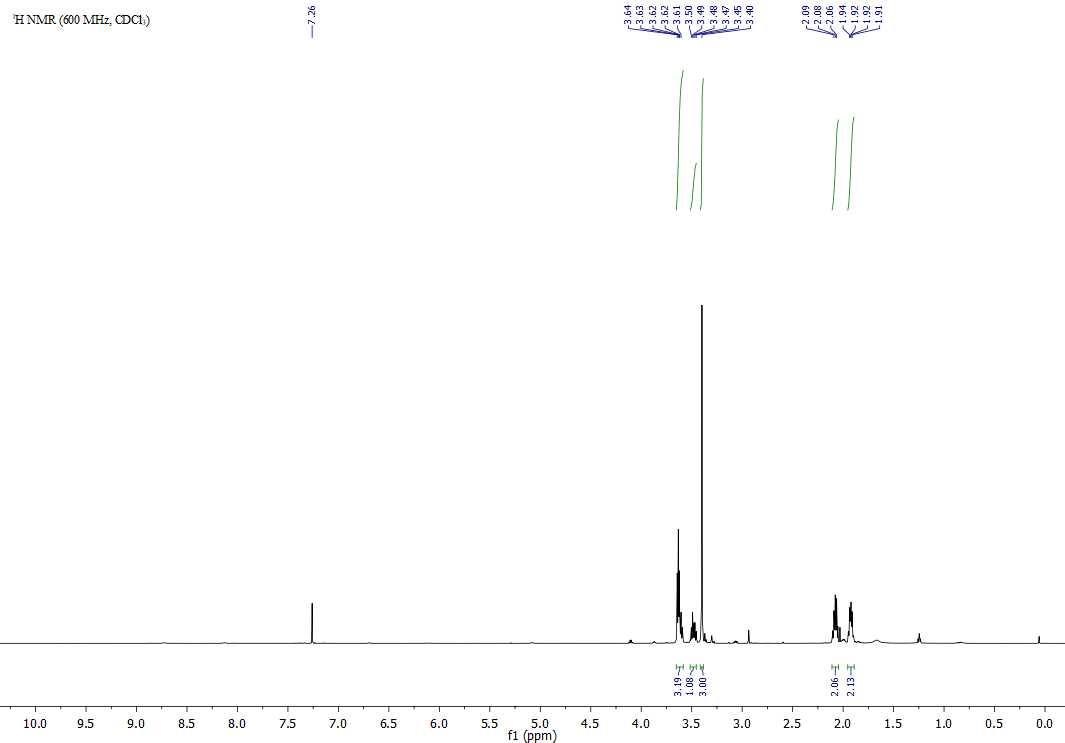
**

**
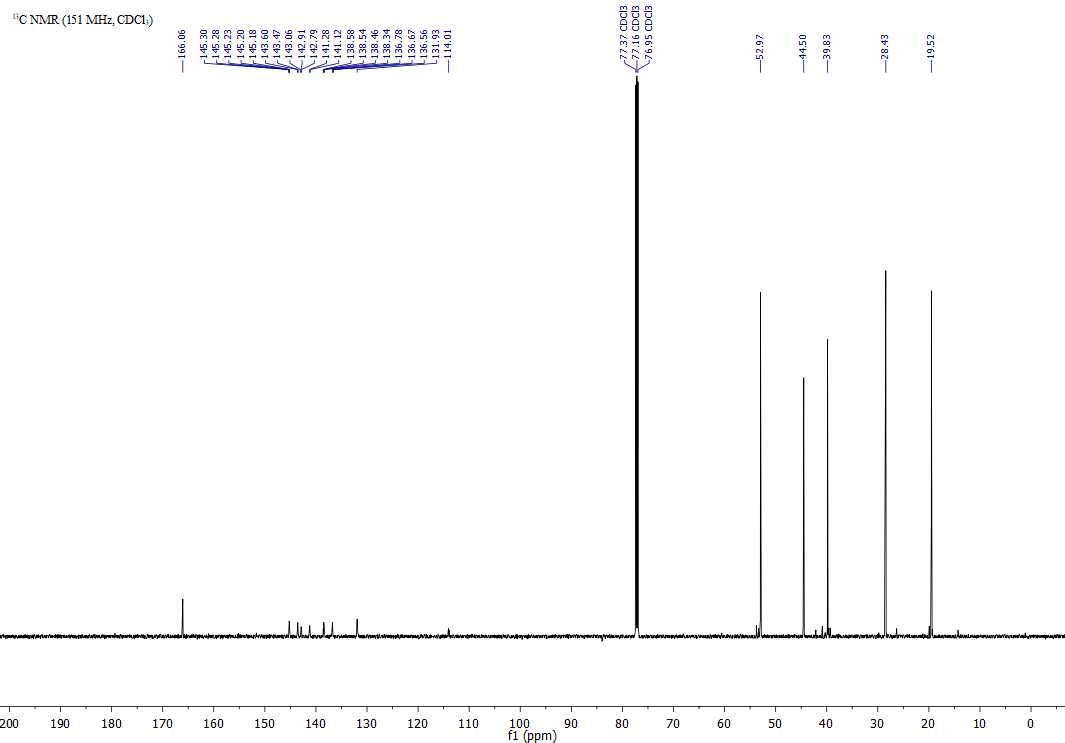
**

**
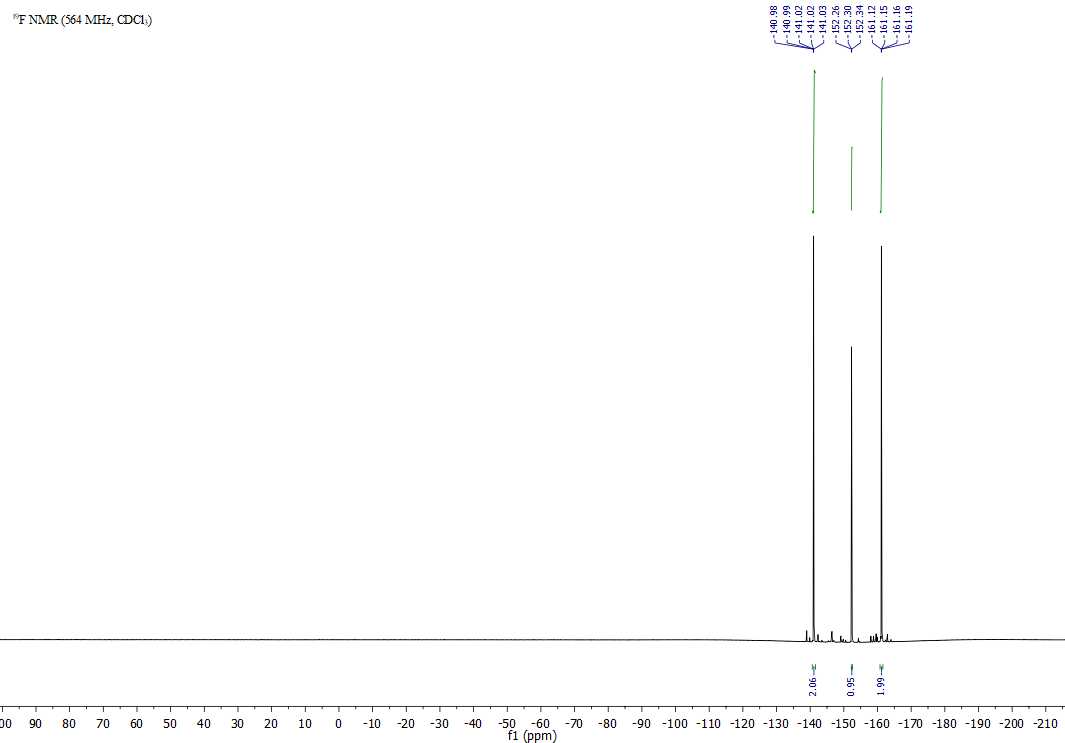
**

**
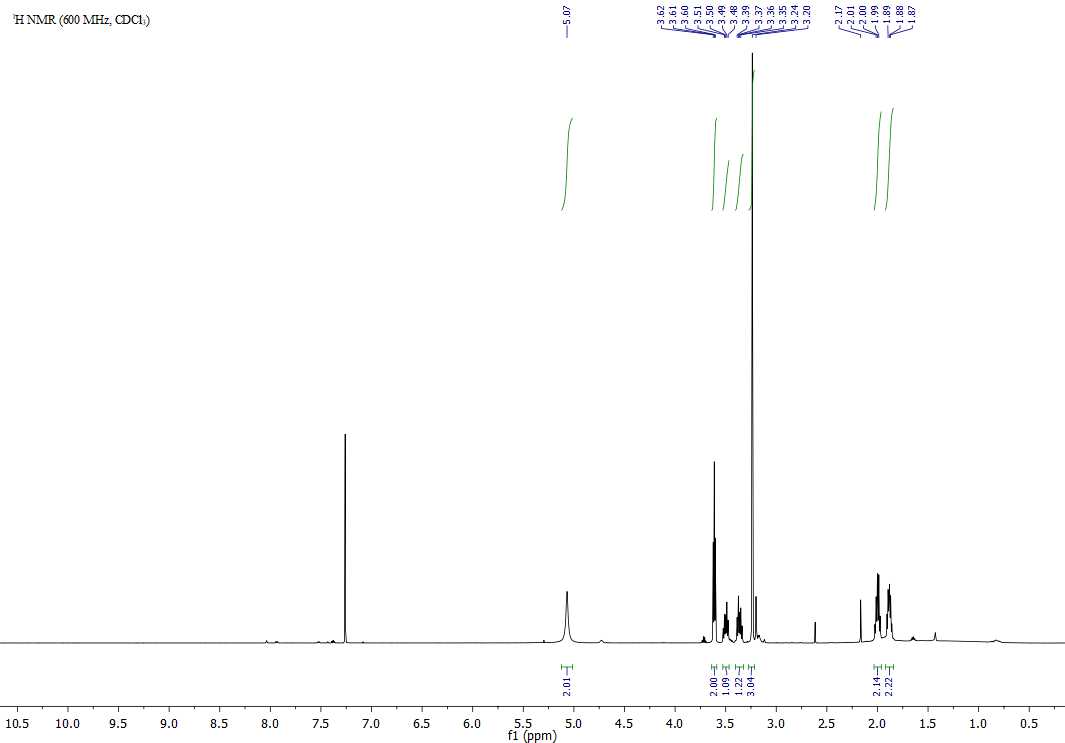
4-(Isothiocyanato)-1-(*N*-carbamoyl-*S*-methyl-sulfonimidoyl)butane (SF135)**

**
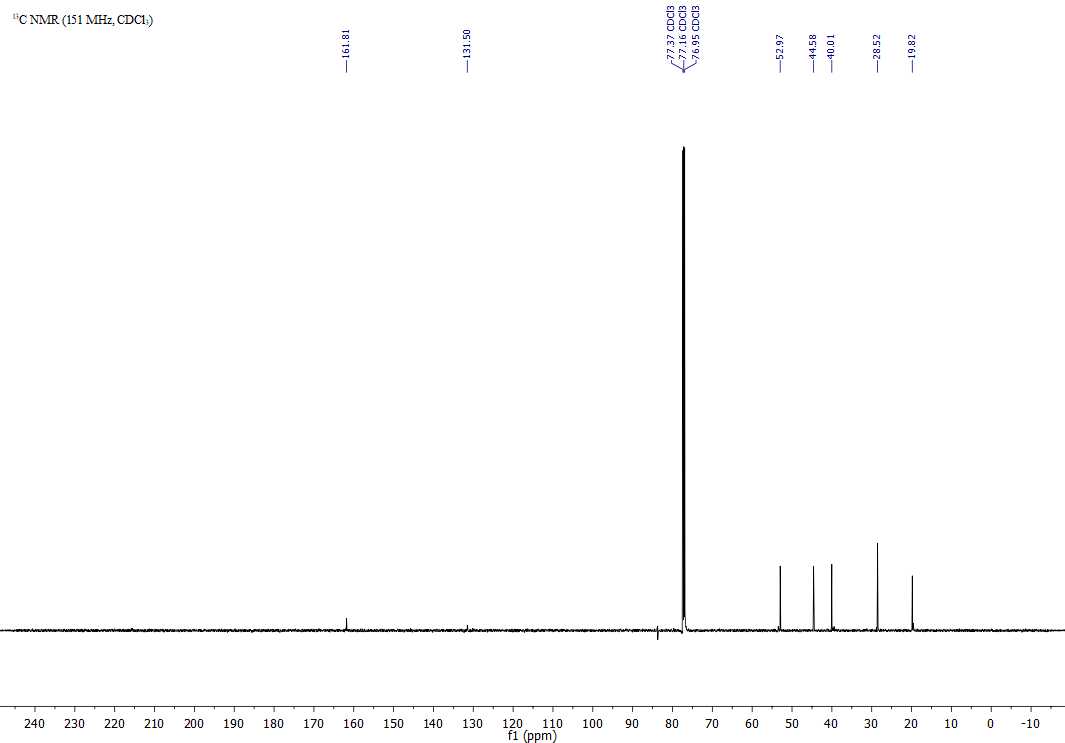
**

**
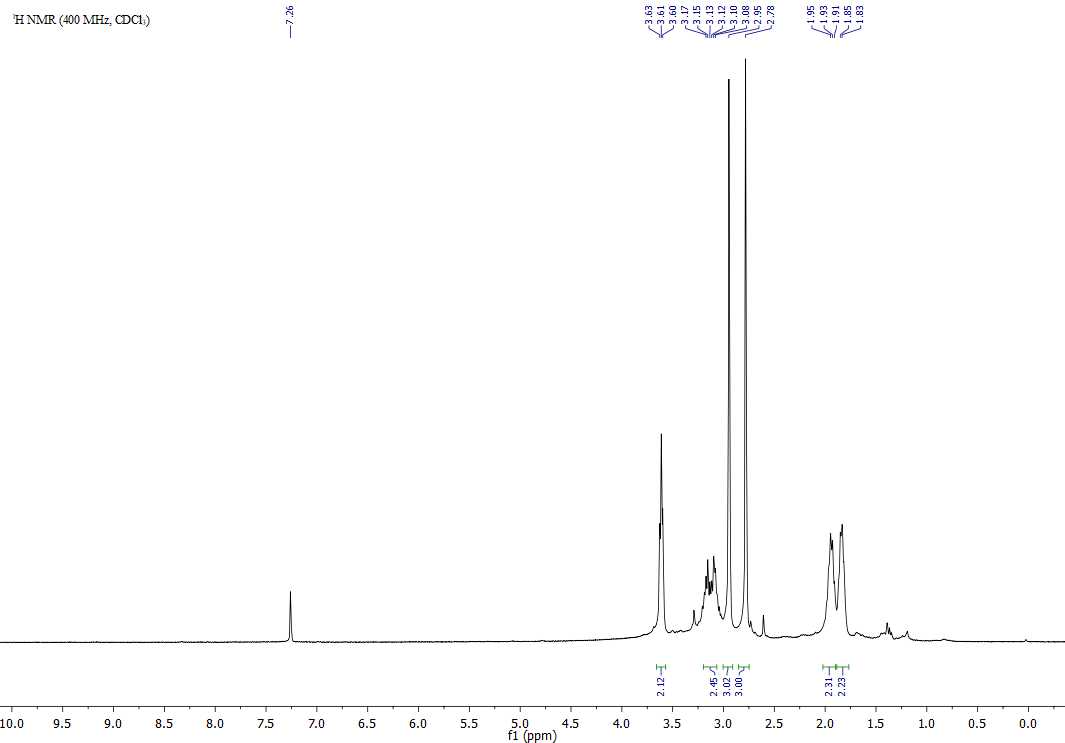
4-(Isothiocyanato)-1-(*N*-methyl-*S*-methylsulfonimidoyl)butane (SF113)**

**
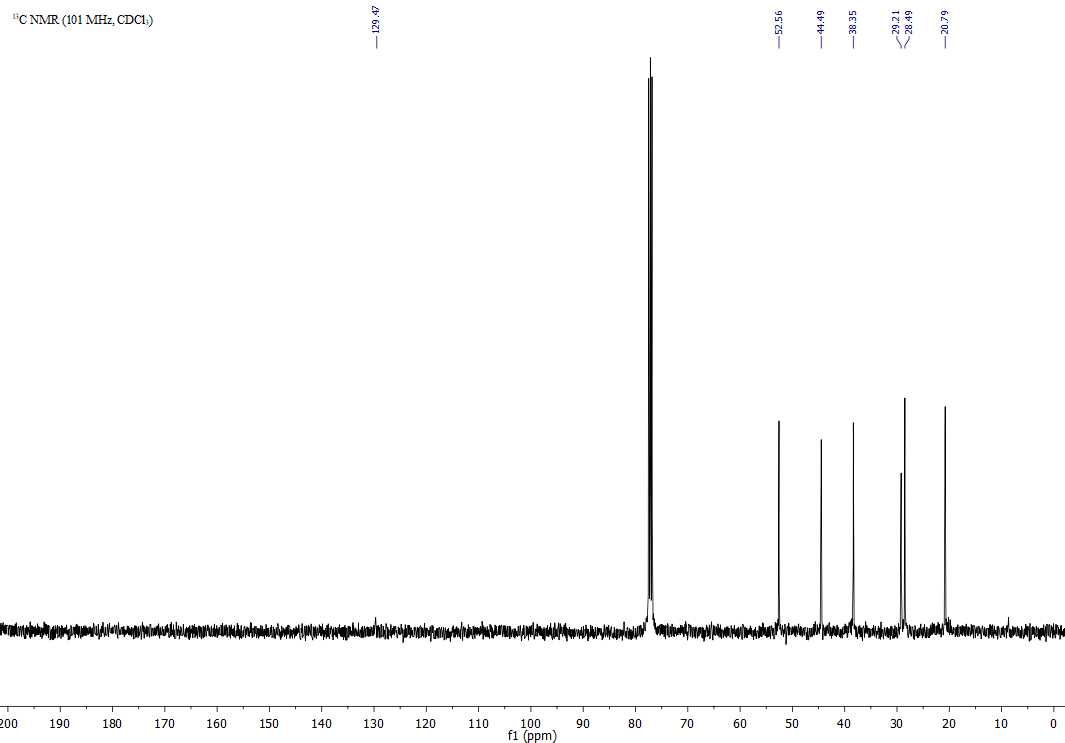
**

**
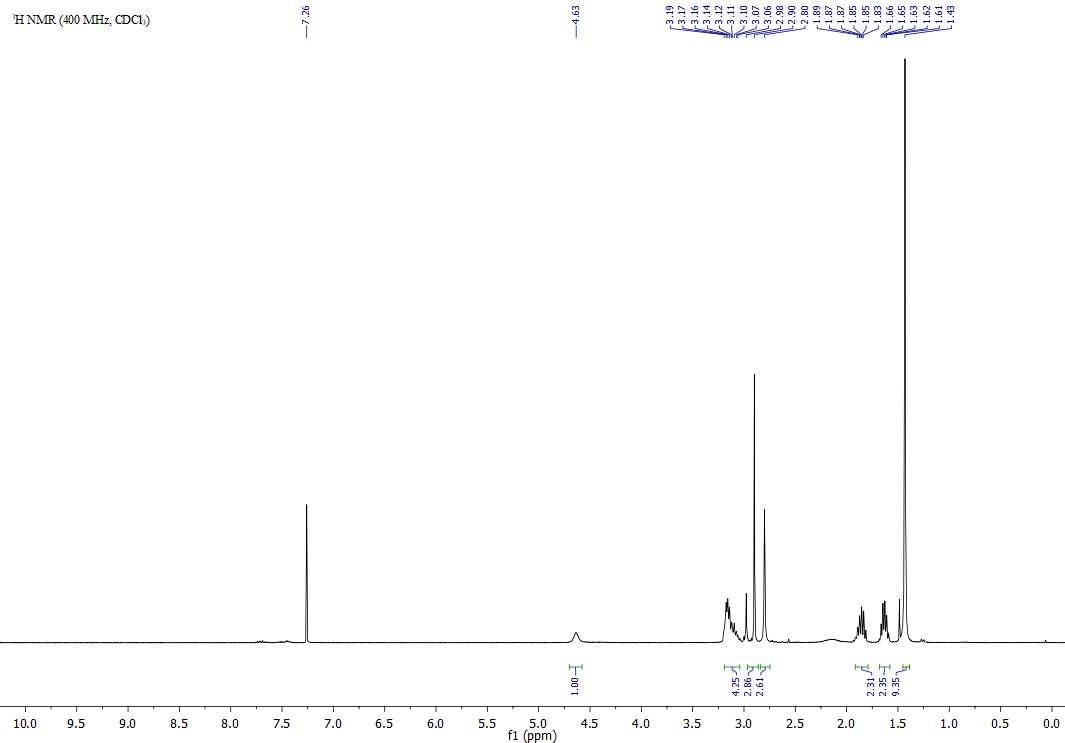
*tert*-Butyl [4-(*N*,*S*-dimethylsulfonimidoyl)butyl]carbamate (5)**

**
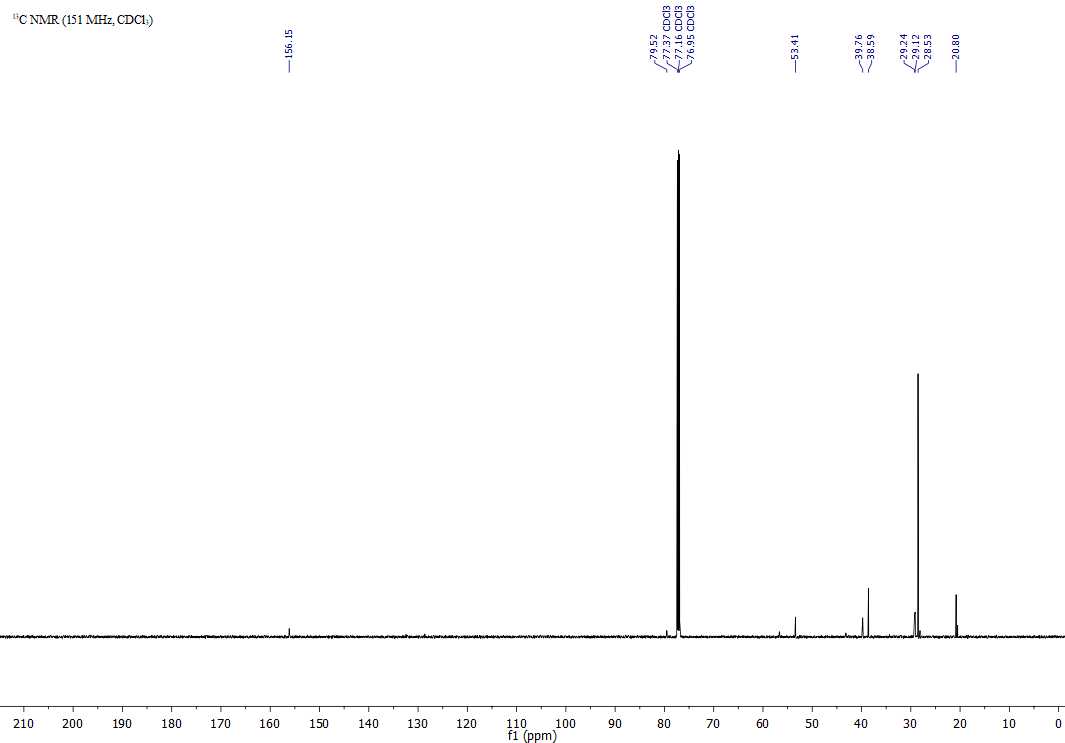
**

***tert*-Butyl [4-(*S*-methyl-*N*-cyano-sulfinimidoyl)butyl]carbamate (6)**

**
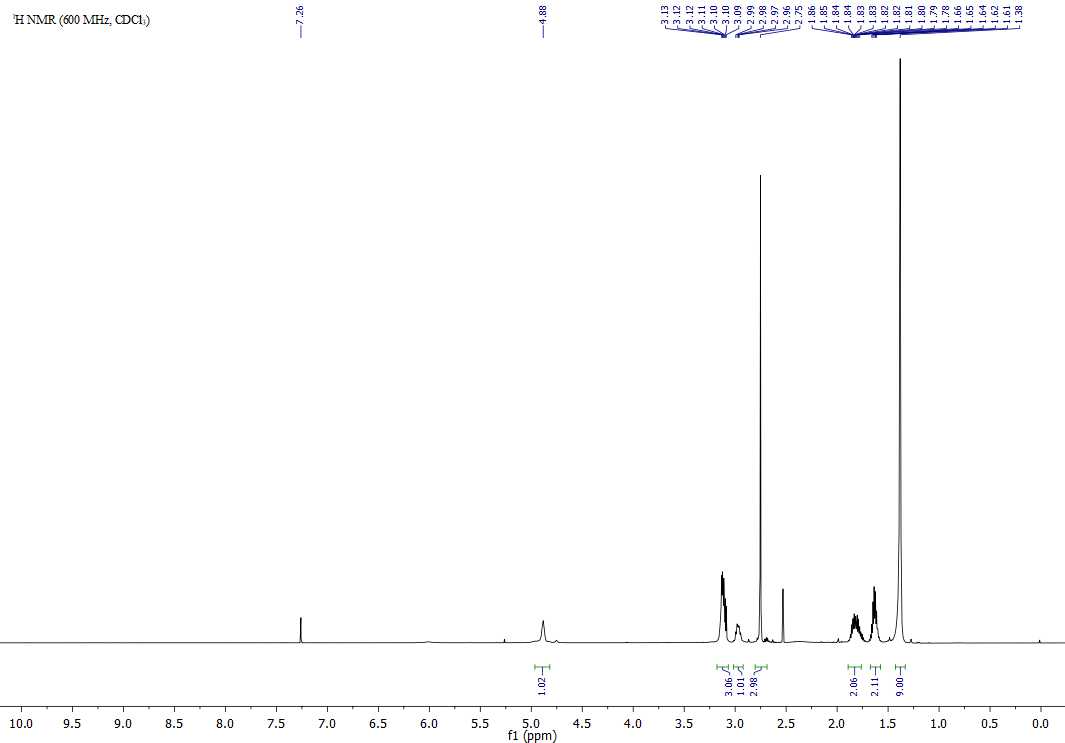
**

**
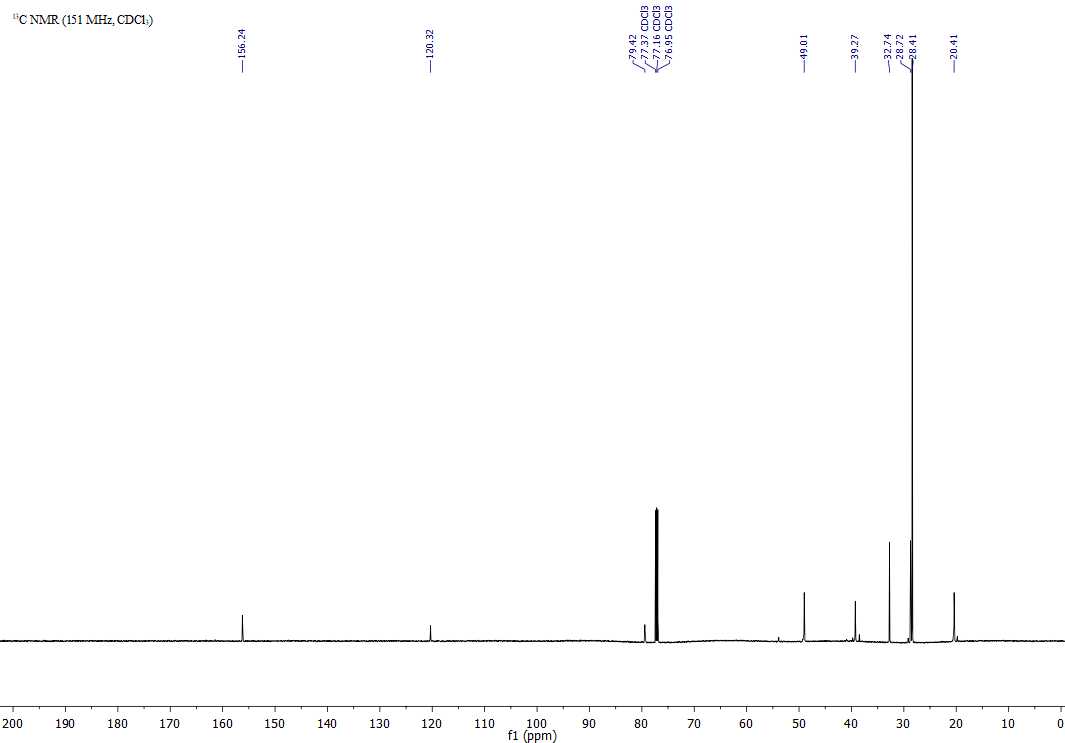
**

**
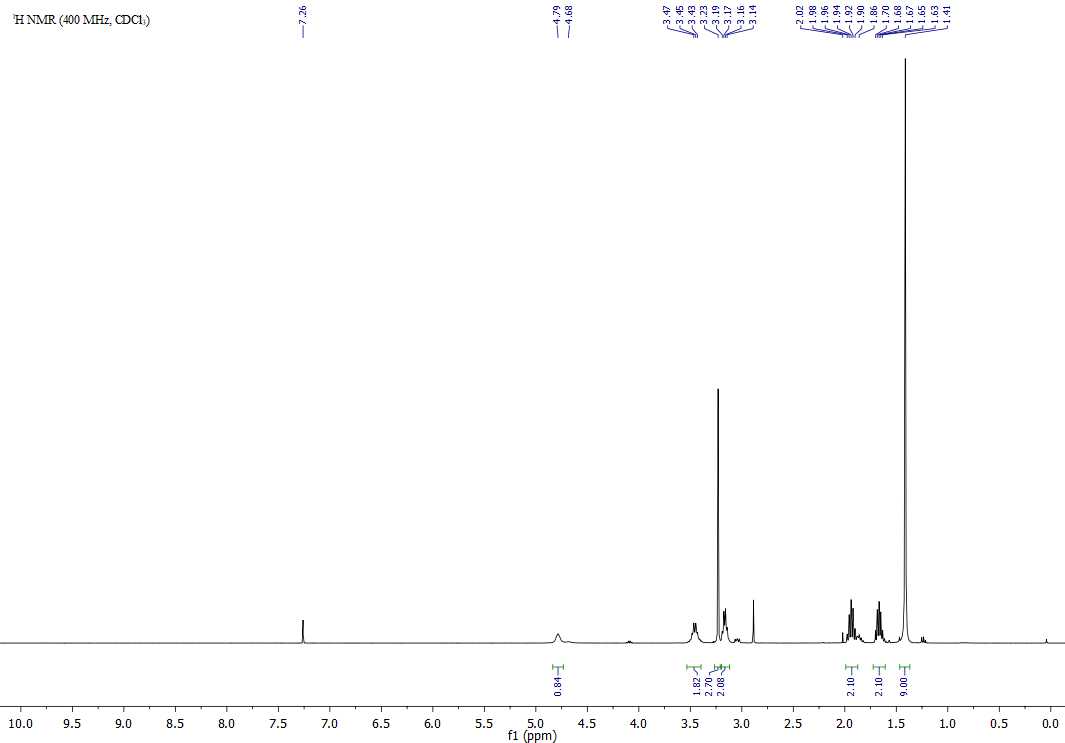
*tert*-Butyl [4-(*S*-methyl-*N*-cyano-sulfonimidoyl)butyl]carbamate (7)**

**
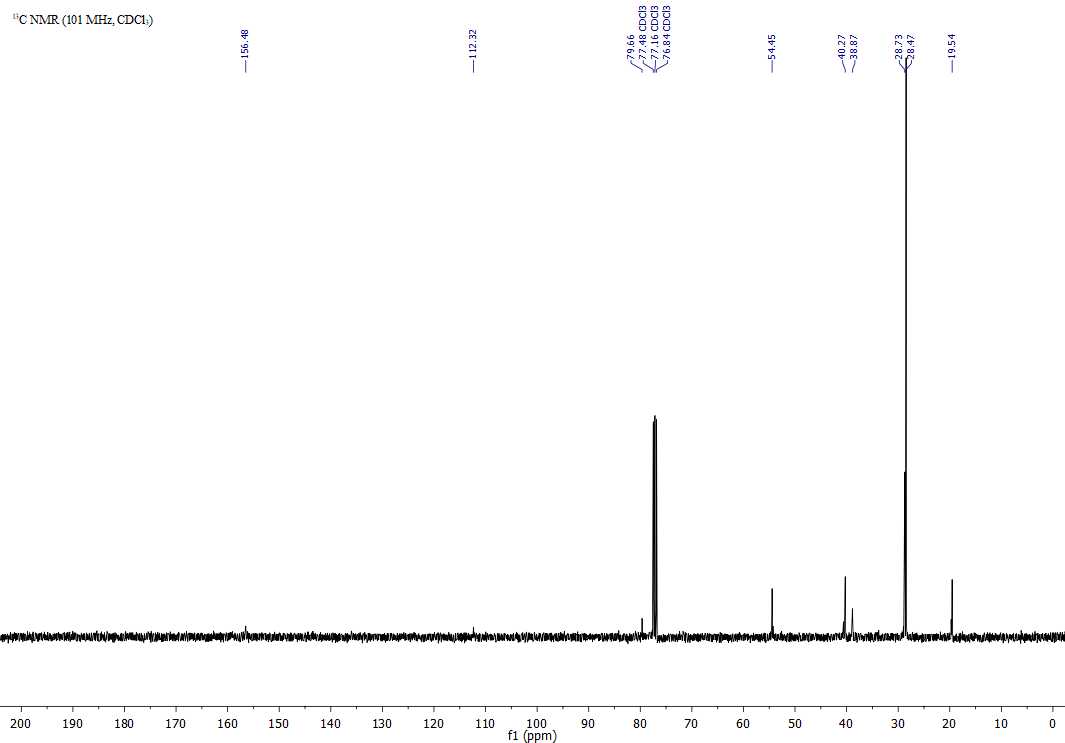
**

***tert*-Butyl [4-(methylsulfinyl)butyl]carbamate (8)**

**
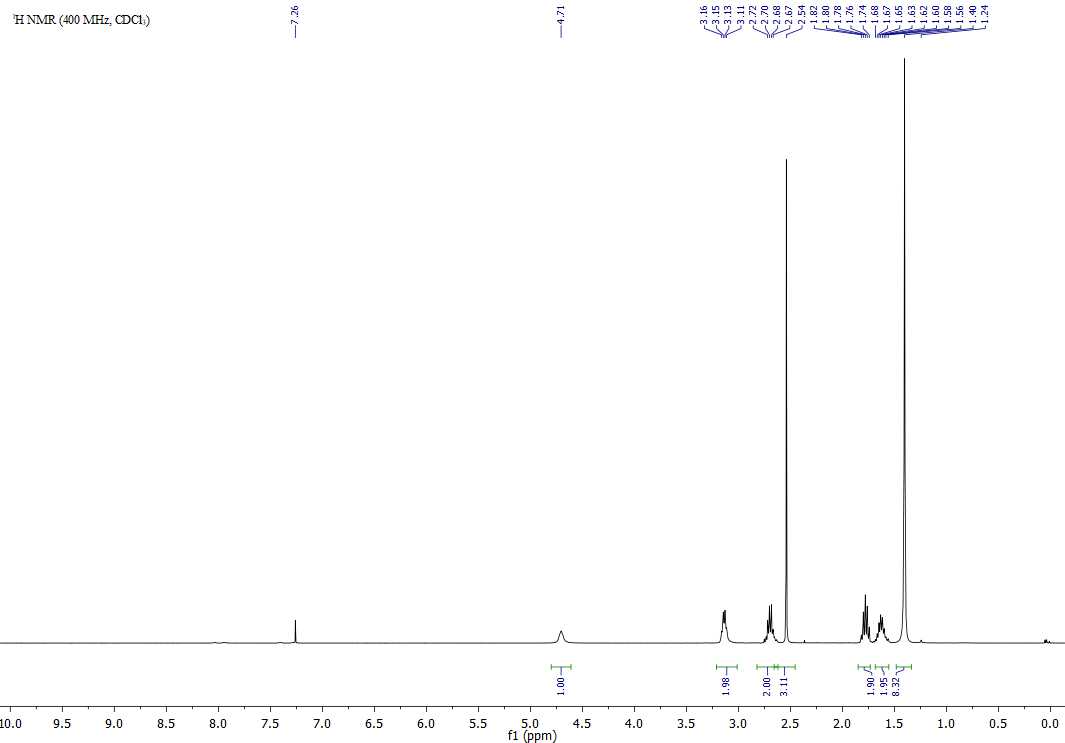
**

**
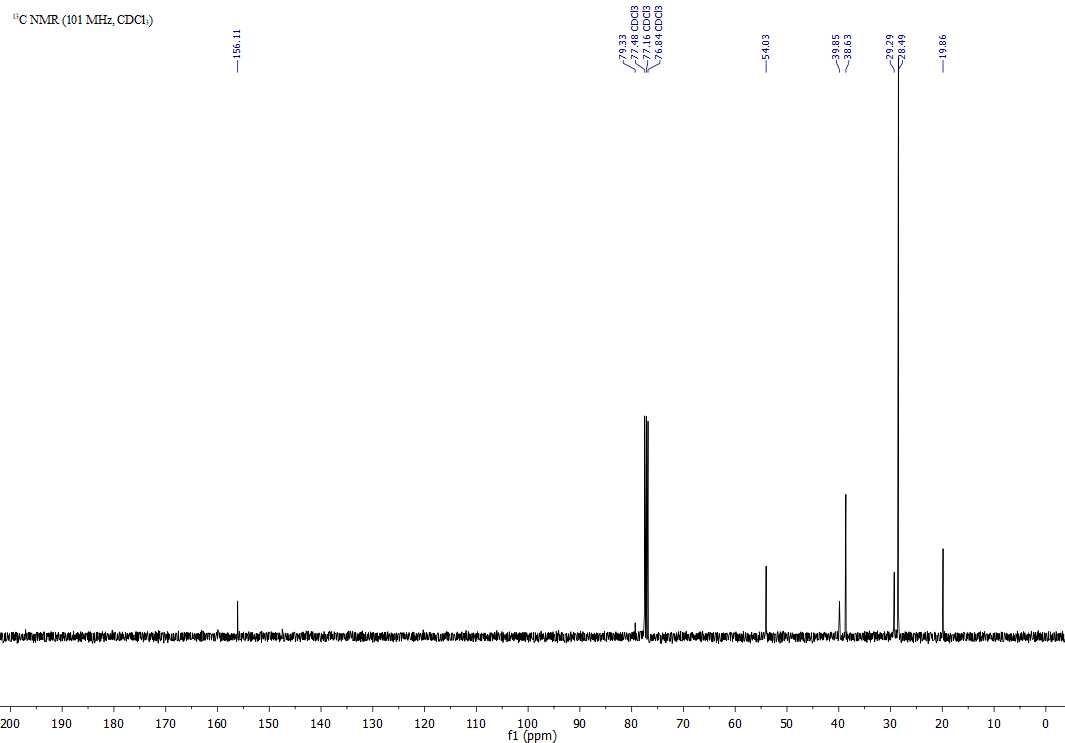
**

***tert*-Butyl {4-[*S*-methyl-*N*-(2,2,2-trifluoroacetyl)sulfonimidoyl]butyl}carbamate (9)**

**
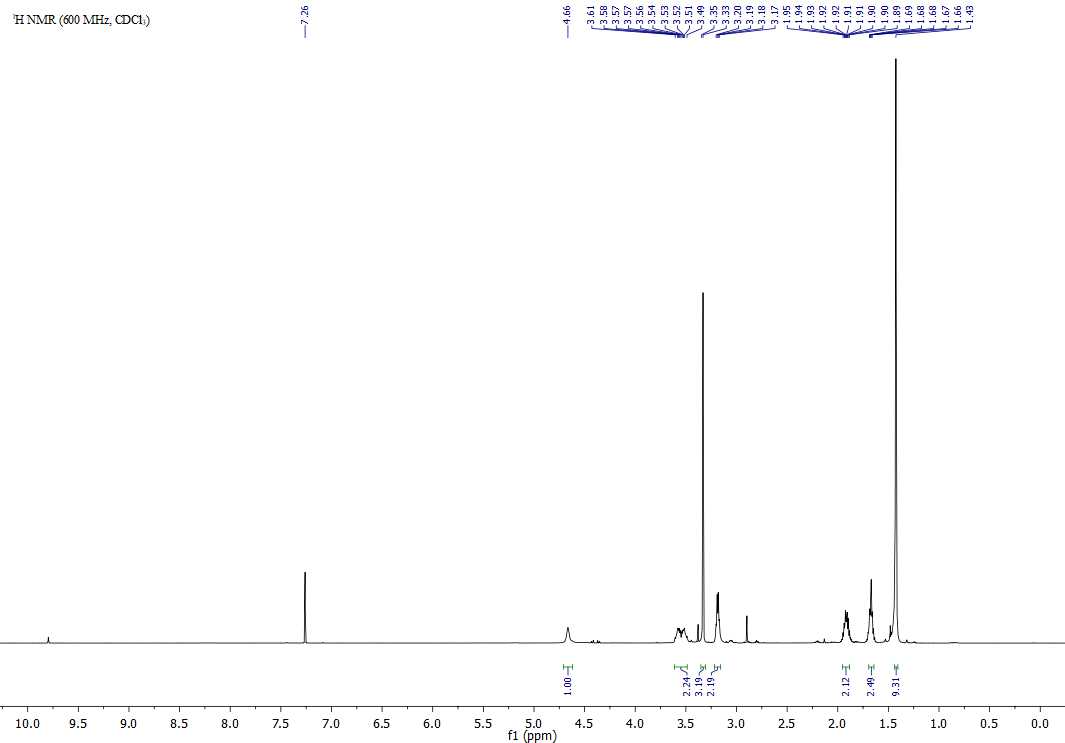
**

**
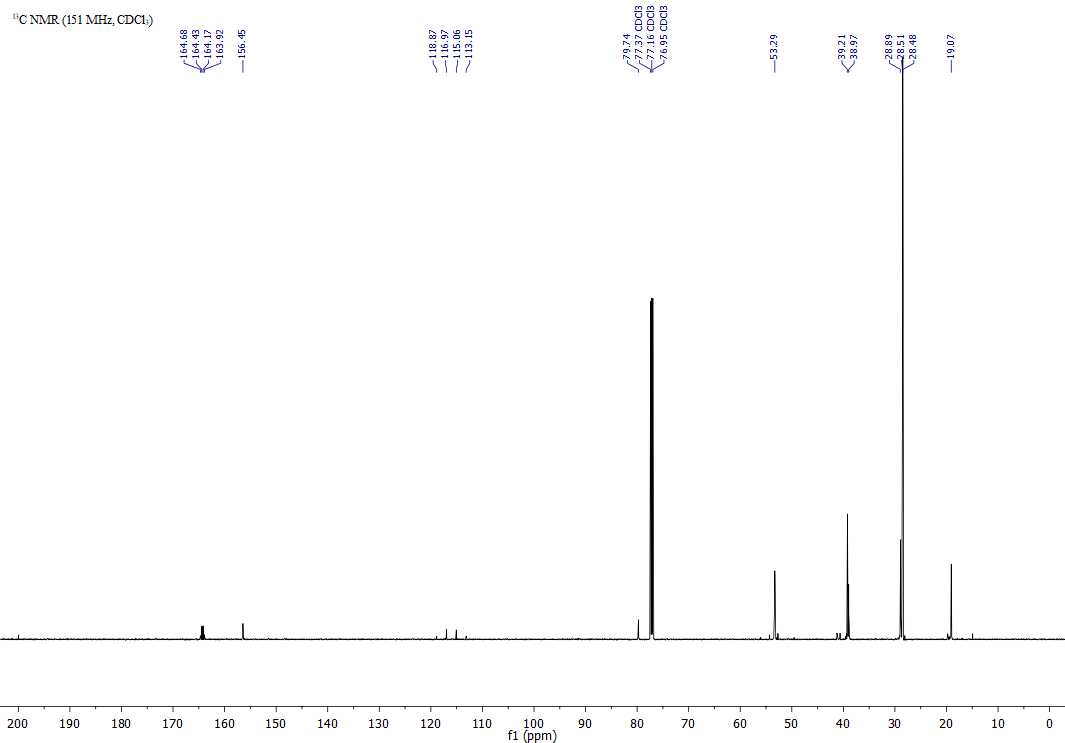
**

**
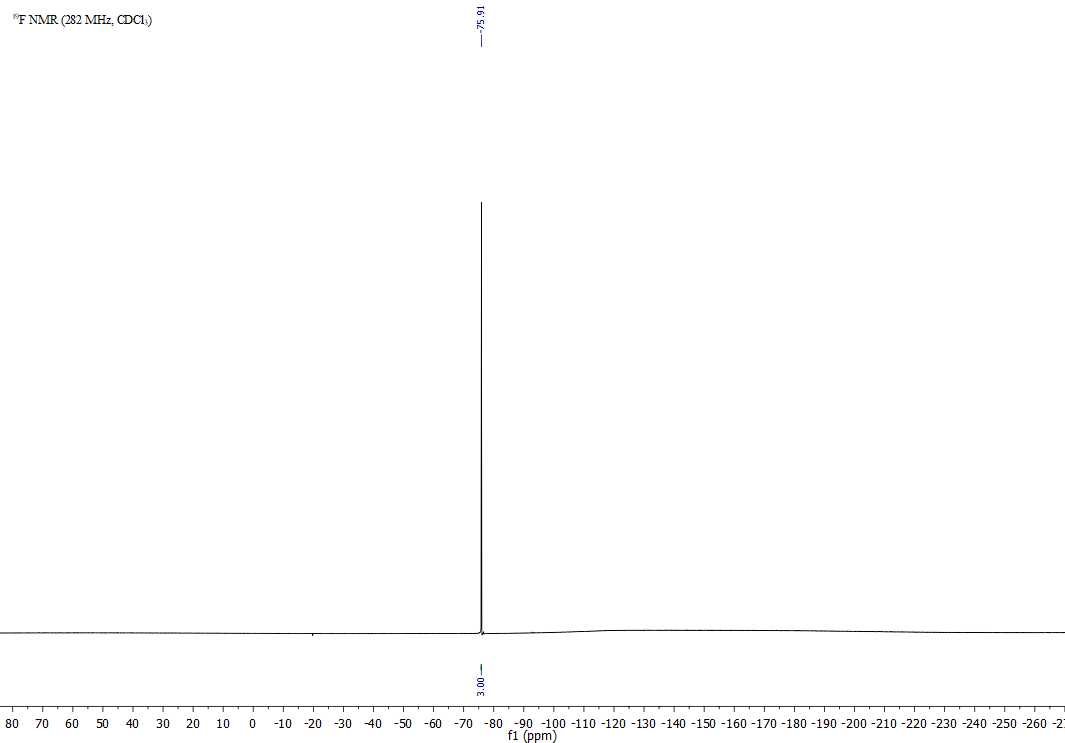
**

**
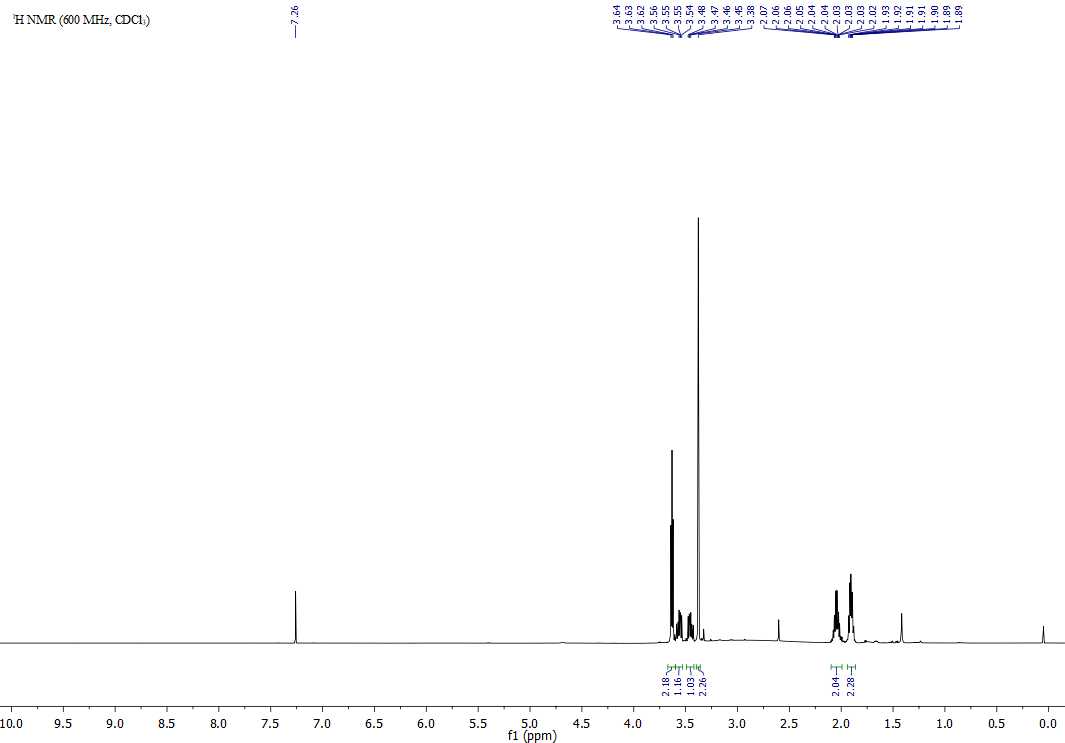
4-(Isothiocyanato)-1-[*N*-(2,2,2-trifluoroacetyl)-*S*-methyl-sulfonimidoyl]butane (SF134) - prepared by the synthesis described in Scheme 2**

**
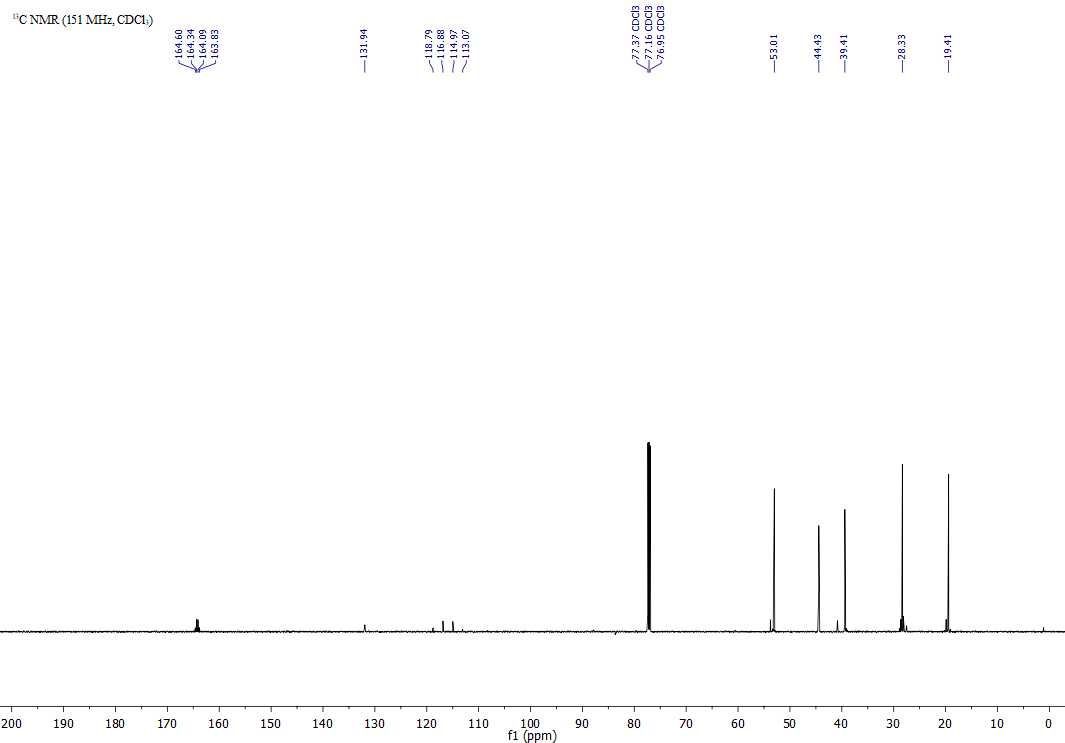
**

**
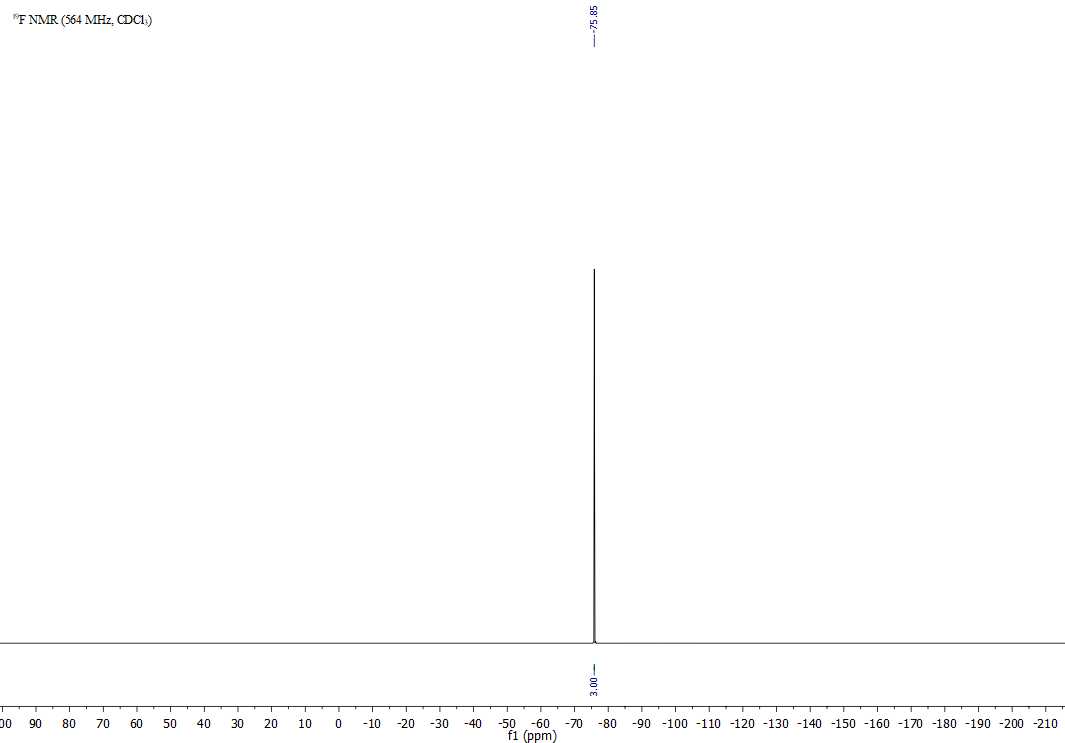
**

**1-Methyltetrahydro-1H-thiophen-1-ium tetrafluoroborate (11)**

**(4-Azidobutyl)(methyl)sulfane (12)**

**(4-Azidobutyl)(imino)(methyl)-λ6-sulfanone (13)**

***N*-[(4-Azidobutyl)(methyl)(oxo)-λ6-sulfaneylidene]-2,2,2-trifluoroacetamide (14)**

***N*-[(4-Azidobutyl)(methyl)(oxo)-λ6-sulfaneylidene]-2,3,4,5,6-pentafluorobenz­amide (15)**

###

**4-(Isothiocyanato)-1-[*N*-(2,2,2-trifluoroacetyl)-*S*-methyl-sulfonimidoyl]butane (SF134)** - prepared from **14** [before subsequent additional purification, containing traces of 2,2,2-trifluoro-*N*-(1-oxidotetrahydro-1λ6-thiophen-1-ylidene)acetamide]

**4-(Isothiocyanato)-1-(*N*-pentafluorobenzoyl-*S*-methyl-sulfonimidoyl)-butane (SF102)** - prepared from **15**
