# Supplementary material for: Novel Broccoli Sulforaphane-Based Analogues Inhibit the Progression of Pancreatic Cancer without Side Effects
Source: Biomolecules. 2020 May 15;10(5):769. doi: 10.3390/biom10050769 (PMC7277136; doi:10.3390/biom10050769)
Supplement: Supplementary file 1 [file biomolecules-10-00769-s001.zip › Biomolecules upload/Suppl_TableS1 Georgikou.pdf]

Table S1 List of miRNA candidates shown in the heatmaps of Figure 6B

Transcript ID(Array Design)	Fold change As SF vs As CO	.-log10(p-Value) for Fold change As SF vs As CO
hsa-miR-92a-1-5p	-1,372521778	3,75505874
hsa-miR-139-5p	1,643831167	2,171386888
hsa-miR-181a-3p	-1,517916889	2,264995123
hsa-miR-23b-5p	-1,320086537	3,641174453
hsa-miR-27b-5p	-1,481057778	6,676530399
hsa-miR-200a-5p	-1,008850574	4,535356109
hsa-miR-933	1,004593711	2,82838023
hsa-miR-2278	-1,454670037	4,497590006
hsa-miR-3617-5p	1,375816648	4,164613745
hsa-miR-29b-1-5p	-0,836535981	5,822817777

Transcript ID(Array Design)	Fold change As 102 vs As CO	.-log10(p-Value) for Fold change As 102 vs As CO
hsa-miR-222-5p	-1,619505926	2,46987786
hsa-miR-608	-0,813965009	2,51964538
hsa-miR-885-3p	-1,1512765	2,14622962
hsa-miR-2278	-1,953776504	5,779851604
hsa-mir-3130-1	0,544790541	2,550401342
hsa-miR-514b-5p	0,961224581	2,110771925
hsa-miR-4784	-0,617792344	4,283023847
hsa-miR-4701-3p	-1,645821574	2,327247792
hsa-miR-4740-5p	1,454254422	2,273906768
hsa-miR-6823-3p	0,841931665	2,209963967

Transcript ID(Array Design)	Fold change As 134 vs As CO	.-log10(p-Value) for Fold change As 134 vs As CO
hsa-miR-509-5p	0,990048778	3,475932331
hsa-miR-550b-2-5p	0,984038667	3,368279238
hsa-miR-885-3p	-1,132580519	2,103883581
hsa-miR-2278	-1,576873574	4,835629473
hsa-miR-4444	1,660302852	2,810881792
hsa-miR-4517	1,279263833	2,004627051
hsa-miR-4640-5p	-1,574678796	2,376120891
hsa-miR-4776-5p	1,217612185	2,226594385
hsa-miR-6729-3p	1,002040461	2,669521405
hsa-miR-525-5p	0,557206904	3,909007438
