# Supplementary material for: Novel Broccoli Sulforaphane-Based Analogues Inhibit the Progression of Pancreatic Cancer without Side Effects
Source: Biomolecules. 2020 May 15;10(5):769. doi: 10.3390/biom10050769 (PMC7277136; doi:10.3390/biom10050769)
Supplement: Supplementary file 1 [file biomolecules-10-00769-s001.zip › Biomolecules upload/Suppl_TableS2 Georgikou.pdf]

Table S2 List of miRNA raw data shown in Volcano plots of Figure 6C

Transcript ID(Array Design)	SF_CO	SF102_SF	SF134_SF
hsa-let-7a-5p	0	0	0
hsa-let-7a-3p	0	0	0
hsa-let-7a-2-3p	0	0	0
hsa-let-7b-5p	0	0	0
hsa-let-7b-3p	0	0	0
hsa-let-7c-5p	0	0	0
hsa-let-7c-3p	0	0	0
hsa-let-7d-5p	0	0	0
hsa-let-7d-3p	0	0	0
hsa-let-7e-5p	0	0	0
hsa-let-7e-3p	0	0	0
hsa-let-7f-5p	0	0	0
hsa-let-7f-1-3p	0	0	0
hsa-let-7f-2-3p	0	0	0
hsa-miR-15a-5p	0	0	0
hsa-miR-15a-3p	0	0	0
hsa-miR-16-5p	0	0	0
hsa-miR-16-1-3p	0	0	0
hsa-miR-17-5p	0	0	0
hsa-miR-17-3p	0	0	0
hsa-miR-18a-5p	0	0	0
hsa-miR-18a-3p	0	0	0
hsa-miR-19a-5p	0	0	0
hsa-miR-19a-3p	0	0	0
hsa-miR-19b-1-5p	0	0	0
hsa-miR-19b-3p	0	0	0
hsa-miR-19b-2-5p	0	0	0
hsa-miR-20a-5p	0	0	0
hsa-miR-20a-3p	0	0	0
hsa-miR-21-5p	0	0	0
hsa-miR-21-3p	0	0	0
hsa-miR-22-5p	0	0	0
hsa-miR-22-3p	1	0	0
hsa-miR-23a-5p	0	0	0
hsa-miR-23a-3p	0	0	0
hsa-miR-24-1-5p	0	0	0
hsa-miR-24-3p	0	0	0
hsa-miR-24-2-5p	0	0	0
hsa-miR-25-5p	0	1	1
hsa-miR-25-3p	0	0	0
hsa-miR-26a-5p	0	0	0
hsa-miR-26a-1-3p	0	0	0
hsa-miR-26b-5p	0	0	0
hsa-miR-26b-3p	0	0	0
hsa-miR-27a-5p	0	0	0
hsa-miR-27a-3p	0	0	0
hsa-miR-28-5p	0	0	0
hsa-miR-28-3p	0	0	0
hsa-miR-29a-5p	0	0	0

hsa-miR-29a-3p	0	0	0
hsa-miR-30a-5p	0	0	0
hsa-miR-30a-3p	0	0	0
hsa-miR-31-5p	0	0	0
hsa-miR-31-3p	0	0	0
hsa-miR-32-5p	0	0	0
hsa-miR-32-3p	0	0	0
hsa-miR-33a-5p	0	0	0
hsa-miR-33a-3p	0	0	0
hsa-miR-92a-1-5p	1	0	0
hsa-miR-92a-3p	0	0	0
hsa-miR-92a-2-5p	0	0	0
hsa-miR-93-5p	0	0	0
hsa-miR-93-3p	0	0	0
hsa-miR-95-5p	0	0	0
hsa-miR-95-3p	0	0	0
hsa-miR-96-5p	0	0	0
hsa-miR-96-3p	0	0	0
hsa-miR-98-5p	0	0	0
hsa-miR-98-3p	0	0	0
hsa-miR-99a-5p	0	0	0
hsa-miR-99a-3p	0	0	0
hsa-miR-100-5p	0	0	0
hsa-miR-100-3p	0	0	0
hsa-miR-101-5p	0	0	0
hsa-miR-101-3p	0	0	0
hsa-miR-29b-1-5p	1	1	1
hsa-miR-29b-3p	0	0	0
hsa-miR-29b-2-5p	0	0	0
hsa-miR-103a-2-5p	0	0	0
hsa-miR-103a-3p	0	0	0
hsa-miR-105-5p	0	0	1
hsa-miR-105-3p	0	0	0
hsa-miR-106a-5p	0	0	0
hsa-miR-106a-3p	0	0	0
hsa-miR-107	0	0	0
hsa-miR-16-2-3p	0	0	0
hsa-miR-192-5p	0	0	0
hsa-miR-192-3p	0	0	0
hsa-miR-196a-5p	0	0	0
hsa-miR-197-5p	0	0	0
hsa-miR-197-3p	0	0	0
hsa-miR-198	0	0	0
hsa-miR-199a-5p	0	0	0
hsa-miR-199a-3p	0	0	0
hsa-miR-208a-5p	0	0	0
hsa-miR-208a-3p	0	0	0
hsa-miR-129-5p	0	0	0
hsa-miR-129-1-3p	0	0	0
hsa-miR-148a-5p	0	0	0
hsa-miR-148a-3p	0	0	0
hsa-miR-30c-5p	0	0	0

hsa-miR-30c-2-3p	0	0	0
hsa-miR-30d-5p	0	0	0
hsa-miR-30d-3p	0	0	0
hsa-miR-139-5p	0	0	0
hsa-miR-139-3p	0	0	0
hsa-miR-147a	0	0	0
hsa-miR-7-5p	0	0	0
hsa-miR-7-1-3p	0	0	0
hsa-miR-7-2-3p	0	0	0
hsa-miR-10a-5p	0	0	0
hsa-miR-10a-3p	0	0	0
hsa-miR-10b-5p	0	0	0
hsa-miR-10b-3p	0	0	0
hsa-miR-34a-5p	0	0	0
hsa-miR-34a-3p	0	0	0
hsa-miR-181a-5p	0	0	0
hsa-miR-181a-2-3p	0	0	0
hsa-miR-181b-5p	0	0	0
hsa-miR-181b-3p	0	0	0
hsa-miR-181c-5p	0	0	0
hsa-miR-181c-3p	0	0	0
hsa-miR-182-5p	0	0	0
hsa-miR-182-3p	0	0	0
hsa-miR-183-5p	0	0	0
hsa-miR-183-3p	0	0	0
hsa-miR-187-5p	0	0	0
hsa-miR-187-3p	0	0	0
hsa-miR-196a-3p	0	0	0
hsa-miR-199b-5p	0	0	0
hsa-miR-199b-3p	0	0	0
hsa-miR-203a	0	0	0
hsa-miR-204-5p	0	0	0
hsa-miR-204-3p	0	0	0
hsa-miR-205-5p	0	0	0
hsa-miR-205-3p	0	0	0
hsa-miR-210-5p	0	0	0
hsa-miR-210-3p	0	0	0
hsa-miR-211-5p	0	0	0
hsa-miR-211-3p	0	0	0
hsa-miR-212-5p	0	0	0
hsa-miR-212-3p	0	0	0
hsa-miR-181a-3p	0	0	0
hsa-miR-214-5p	0	0	0
hsa-miR-214-3p	0	0	0
hsa-miR-215-5p	0	0	0
hsa-miR-215-3p	0	0	0
hsa-miR-216a-5p	0	0	0
hsa-miR-216a-3p	0	0	0
hsa-miR-217	0	0	0
hsa-miR-218-5p	0	0	0
hsa-miR-218-1-3p	0	0	0
hsa-miR-218-2-3p	0	0	0

hsa-miR-219a-5p	0	0	0
hsa-miR-219a-1-3p	0	0	0
hsa-miR-221-5p	0	0	0
hsa-miR-221-3p	0	0	0
hsa-miR-222-5p	0	0	0
hsa-miR-222-3p	0	0	0
hsa-miR-223-5p	0	0	0
hsa-miR-223-3p	0	0	0
hsa-miR-224-5p	0	0	0
hsa-miR-224-3p	0	0	0
hsa-miR-200b-5p	1	0	0
hsa-miR-200b-3p	0	0	0
hsa-let-7g-5p	0	0	0
hsa-let-7g-3p	0	0	0
hsa-let-7i-5p	0	0	0
hsa-let-7i-3p	0	0	0
hsa-miR-1	0	0	0
hsa-miR-15b-5p	0	0	0
hsa-miR-15b-3p	0	0	0
hsa-miR-23b-5p	1	0	0
hsa-miR-23b-3p	0	0	0
hsa-miR-27b-5p	1	1	1
hsa-miR-27b-3p	0	0	0
hsa-miR-30b-5p	0	0	0
hsa-miR-30b-3p	0	0	0
hsa-miR-122-5p	0	0	0
hsa-miR-122-3p	0	0	0
hsa-miR-124-5p	0	0	0
hsa-miR-124-3p	0	0	0
hsa-miR-125b-5p	0	0	0
hsa-miR-125b-1-3p	0	0	0
hsa-miR-128-1-5p	0	0	0
hsa-miR-128-3p	0	0	0
hsa-miR-130a-5p	0	0	0
hsa-miR-130a-3p	0	0	0
hsa-miR-132-5p	0	0	0
hsa-miR-132-3p	0	0	0
hsa-miR-133a-5p	0	0	0
hsa-miR-133a-3p	0	0	0
hsa-miR-135a-5p	0	0	0
hsa-miR-135a-3p	0	0	0
hsa-miR-137	0	0	0
hsa-miR-138-5p	0	0	0
hsa-miR-138-2-3p	0	0	0
hsa-miR-140-5p	0	0	0
hsa-miR-140-3p	0	0	0
hsa-miR-141-5p	0	0	0
hsa-miR-141-3p	0	0	0
hsa-miR-142-5p	0	0	0
hsa-miR-142-3p	0	0	0
hsa-miR-143-5p	0	0	0
hsa-miR-143-3p	0	0	0

hsa-miR-154-5p	0	0	0
hsa-miR-144-3p	0	0	0
hsa-miR-145-5p	0	0	0
hsa-miR-145-3p	0	0	0
hsa-miR-152-5p	0	0	0
hsa-miR-152-3p	0	0	0
hsa-miR-153-3p	0	0	0
hsa-miR-153-5p	0	0	0
hsa-miR-191-5p	0	0	0
hsa-miR-191-3p	0	0	0
hsa-miR-9-5p	0	0	0
hsa-miR-9-3p	0	0	0
hsa-miR-125a-5p	0	0	0
hsa-miR-125a-3p	0	0	0
hsa-miR-125b-2-3p	0	0	0
hsa-miR-126-5p	0	0	0
hsa-miR-126-3p	0	0	0
hsa-miR-127-5p	0	0	0
hsa-miR-127-3p	0	0	0
hsa-miR-129-2-3p	0	0	0
hsa-miR-134-5p	0	0	0
hsa-miR-134-3p	0	0	0
hsa-miR-136-5p	0	0	0
hsa-miR-136-3p	0	0	0
hsa-miR-138-1-3p	0	0	0
hsa-miR-146a-5p	0	0	0
hsa-miR-146a-3p	0	0	0
hsa-miR-149-5p	0	0	0
hsa-miR-149-3p	0	0	0
hsa-miR-150-5p	0	0	0
hsa-miR-150-3p	0	0	0
hsa-miR-154-5p	0	0	0
hsa-miR-154-3p	0	0	0
hsa-miR-184	0	0	0
hsa-miR-185-5p	0	0	0
hsa-miR-185-3p	0	0	0
hsa-miR-186-5p	0	0	0
hsa-miR-186-3p	0	0	0
hsa-miR-188-5p	0	0	0
hsa-miR-188-3p	0	0	0
hsa-miR-190a-5p	0	0	0
hsa-miR-190a-3p	0	0	0
hsa-miR-193a-5p	0	0	0
hsa-miR-193a-3p	0	0	0
hsa-miR-194-5p	0	0	0
hsa-miR-195-5p	0	0	0
hsa-miR-195-3p	0	0	0
hsa-miR-206	0	0	0
hsa-miR-320a	0	0	0
hsa-miR-200c-5p	0	0	0
hsa-miR-200c-3p	0	0	0
hsa-miR-155-5p	0	0	0

hsa-miR-155-3p	0	0	0
hsa-miR-128-2-5p	0	0	0
hsa-miR-194-3p	0	0	0
hsa-miR-106b-5p	0	0	0
hsa-miR-106b-3p	0	0	0
hsa-miR-29c-5p	0	0	0
hsa-miR-29c-3p	0	0	0
hsa-miR-30c-1-3p	0	0	0
hsa-miR-200a-5p	1	0	0
hsa-miR-200a-3p	0	0	0
hsa-miR-302a-5p	0	0	0
hsa-miR-302a-3p	0	0	0
hsa-miR-219a-2-3p	0	0	0
hsa-miR-34b-5p	0	0	0
hsa-miR-34b-3p	0	0	0
hsa-miR-34c-5p	0	0	0
hsa-miR-34c-3p	0	0	0
hsa-miR-299-5p	0	0	0
hsa-miR-299-3p	0	0	0
hsa-miR-301a-5p	0	0	0
hsa-miR-301a-3p	0	0	0
hsa-miR-99b-5p	0	0	0
hsa-miR-99b-3p	0	0	0
hsa-miR-296-5p	0	0	0
hsa-miR-296-3p	0	0	0
hsa-miR-130b-5p	0	0	0
hsa-miR-130b-3p	0	0	0
hsa-miR-30e-5p	0	0	0
hsa-miR-30e-3p	0	0	0
hsa-miR-26a-2-3p	0	0	0
hsa-miR-361-5p	0	0	0
hsa-miR-361-3p	0	0	0
hsa-miR-362-5p	0	0	0
hsa-miR-362-3p	0	0	0
hsa-miR-363-5p	0	0	0
hsa-miR-363-3p	0	0	0
hsa-miR-365a-5p	0	0	0
hsa-miR-365a-3p	0	0	0
hsa-miR-365b-5p	0	0	0
hsa-miR-365b-3p	0	0	0
hsa-miR-302b-5p	0	0	0
hsa-miR-302b-3p	0	0	0
hsa-miR-302c-5p	0	0	0
hsa-miR-302c-3p	0	0	0
hsa-miR-302d-5p	0	0	0
hsa-miR-302d-3p	0	0	0
hsa-miR-367-5p	0	0	0
hsa-miR-367-3p	0	0	0
hsa-miR-376c-5p	0	0	0
hsa-miR-376c-3p	0	0	0
hsa-miR-369-5p	0	0	0
hsa-miR-369-3p	0	0	0

hsa-miR-370-5p	0	0	0
hsa-miR-370-3p	0	0	0
hsa-miR-371a-5p	0	0	0
hsa-miR-371a-3p	0	0	0
hsa-miR-372-5p	0	0	0
hsa-miR-372-3p	0	0	0
hsa-miR-373-5p	0	0	0
hsa-miR-373-3p	0	0	0
hsa-miR-374a-5p	0	0	0
hsa-miR-374a-3p	0	0	0
hsa-miR-375	0	0	0
hsa-miR-376a-5p	0	0	0
hsa-miR-376a-3p	0	0	0
hsa-miR-377-5p	0	0	0
hsa-miR-377-3p	0	0	0
hsa-miR-378a-5p	0	0	0
hsa-miR-378a-3p	0	0	0
hsa-miR-379-5p	0	0	0
hsa-miR-379-3p	0	0	0
hsa-miR-380-5p	0	0	0
hsa-miR-380-3p	0	0	0
hsa-miR-381-5p	0	0	0
hsa-miR-381-3p	0	0	0
hsa-miR-382-5p	0	0	0
hsa-miR-382-3p	0	0	0
hsa-miR-383-5p	0	0	0
hsa-miR-383-3p	0	0	0
hsa-miR-340-5p	0	0	0
hsa-miR-340-3p	0	0	0
hsa-miR-330-5p	0	0	0
hsa-miR-330-3p	0	0	0
hsa-miR-328-5p	0	0	0
hsa-miR-328-3p	0	0	0
hsa-miR-342-5p	0	0	0
hsa-miR-342-3p	0	0	0
hsa-miR-337-5p	0	0	0
hsa-miR-337-3p	0	0	0
hsa-miR-323a-5p	0	0	0
hsa-miR-323a-3p	0	0	0
hsa-miR-326	0	0	0
hsa-miR-151a-5p	0	0	0
hsa-miR-151a-3p	0	0	0
hsa-miR-135b-5p	0	0	0
hsa-miR-135b-3p	0	0	0
hsa-miR-148b-5p	0	0	0
hsa-miR-148b-3p	0	0	0
hsa-miR-331-5p	0	0	0
hsa-miR-331-3p	0	0	0
hsa-miR-324-5p	0	0	0
hsa-miR-324-3p	0	0	0
hsa-miR-338-5p	0	0	0
hsa-miR-338-3p	0	0	0

hsa-miR-339-5p	0	0	0
hsa-miR-339-3p	0	0	0
hsa-miR-335-5p	0	0	0
hsa-miR-335-3p	0	0	0
hsa-miR-133b	0	0	0
hsa-miR-325	0	0	0
hsa-miR-345-5p	0	0	0
hsa-miR-345-3p	0	0	0
hsa-miR-346	0	0	0
hsa-miR-384	0	0	0
hsa-miR-196b-5p	0	0	0
hsa-miR-196b-3p	0	0	0
hsa-miR-422a	0	0	0
hsa-miR-423-5p	0	0	0
hsa-miR-423-3p	0	0	0
hsa-miR-424-5p	0	0	0
hsa-miR-424-3p	0	0	0
hsa-miR-425-5p	0	0	0
hsa-miR-425-3p	0	0	0
hsa-miR-18b-5p	0	0	0
hsa-miR-18b-3p	0	0	0
hsa-miR-20b-5p	0	0	0
hsa-miR-20b-3p	0	0	0
hsa-miR-448	0	0	0
hsa-miR-429	0	0	0
hsa-miR-449a	0	0	0
hsa-miR-450a-5p	0	0	0
hsa-miR-450a-1-3p	0	0	0
hsa-miR-431-5p	0	0	0
hsa-miR-431-3p	0	0	0
hsa-miR-433-5p	0	0	0
hsa-miR-433-3p	0	0	0
hsa-miR-329-5p	0	0	0
hsa-miR-329-3p	0	0	0
hsa-miR-451a	0	0	0
hsa-miR-452-5p	0	0	0
hsa-miR-452-3p	0	0	0
hsa-miR-409-5p	0	0	0
hsa-miR-409-3p	0	0	0
hsa-miR-412-5p	0	0	0
hsa-miR-412-3p	0	0	0
hsa-miR-410-5p	0	0	0
hsa-miR-410-3p	0	0	0
hsa-miR-376b-5p	0	0	0
hsa-miR-376b-3p	0	0	0
hsa-miR-483-5p	0	0	0
hsa-miR-483-3p	0	0	0
hsa-miR-484	0	0	0
hsa-miR-485-5p	0	0	0
hsa-miR-485-3p	0	0	0
hsa-miR-486-5p	0	0	0
hsa-miR-486-3p	0	0	0

hsa-miR-487a-5p	0	0	0
hsa-miR-487a-3p	0	0	0
hsa-miR-488-5p	0	0	0
hsa-miR-488-3p	0	0	0
hsa-miR-489-5p	0	0	0
hsa-miR-489-3p	0	0	0
hsa-miR-490-5p	0	0	0
hsa-miR-490-3p	0	0	0
hsa-miR-491-5p	0	0	0
hsa-miR-491-3p	0	0	0
hsa-miR-511-5p	0	0	0
hsa-miR-511-3p	0	0	0
hsa-miR-146b-5p	0	0	0
hsa-miR-146b-3p	0	0	0
hsa-miR-202-5p	0	0	0
hsa-miR-202-3p	0	0	0
hsa-miR-492	0	0	0
hsa-miR-493-5p	0	0	0
hsa-miR-493-3p	0	0	0
hsa-miR-432-5p	0	0	0
hsa-miR-432-3p	0	0	0
hsa-miR-494-5p	0	0	0
hsa-miR-494-3p	0	0	0
hsa-miR-495-5p	0	0	0
hsa-miR-495-3p	0	0	0
hsa-miR-496	0	0	0
hsa-miR-193b-5p	0	0	0
hsa-miR-193b-3p	0	0	0
hsa-miR-497-5p	0	0	0
hsa-miR-497-3p	0	0	0
hsa-miR-181d-5p	0	0	0
hsa-miR-181d-3p	0	0	0
hsa-miR-512-5p	0	0	0
hsa-miR-512-3p	0	0	0
hsa-miR-498	0	0	0
hsa-miR-520e	0	0	0
hsa-miR-515-5p	0	0	0
hsa-miR-515-3p	0	0	0
hsa-miR-519e-5p	0	0	0
hsa-miR-519e-3p	0	0	0
hsa-miR-520f-5p	0	0	0
hsa-miR-520f-3p	0	0	0
hsa-miR-519c-5p	0	0	0
hsa-miR-519c-3p	0	0	0
hsa-miR-520a-5p	0	0	0
hsa-miR-520a-3p	0	0	0
hsa-miR-526b-5p	0	0	0
hsa-miR-526b-3p	0	0	0
hsa-miR-519b-5p	0	0	0
hsa-miR-519b-3p	0	0	0
hsa-miR-525-5p	1	0	0
hsa-miR-525-3p	0	0	0

hsa-miR-523-5p	0	0	0
hsa-miR-523-3p	0	0	0
hsa-miR-518f-5p	0	0	0
hsa-miR-518f-3p	0	0	0
hsa-miR-520b	0	0	0
hsa-miR-518b	0	0	0
hsa-miR-526a	0	0	0
hsa-miR-520c-5p	0	0	0
hsa-miR-520c-3p	0	0	0
hsa-miR-518c-5p	0	0	0
hsa-miR-518c-3p	0	0	0
hsa-miR-524-5p	0	0	0
hsa-miR-524-3p	0	0	0
hsa-miR-517-5p	0	0	0
hsa-miR-517a-3p	0	0	0
hsa-miR-519d-5p	0	0	0
hsa-miR-519d-3p	0	0	0
hsa-miR-521	0	0	0
hsa-miR-520d-5p	0	0	0
hsa-miR-520d-3p	0	0	0
hsa-miR-517b-3p	0	0	0
hsa-miR-520g-5p	0	0	0
hsa-miR-520g-3p	0	0	0
hsa-miR-516b-5p	0	0	0
hsa-miR-516b-3p	0	0	0
hsa-miR-518e-5p	0	0	0
hsa-miR-518e-3p	0	0	0
hsa-miR-518a-5p	0	0	0
hsa-miR-518a-3p	0	0	0
hsa-miR-518d-5p	0	0	0
hsa-miR-518d-3p	0	0	0
hsa-miR-517c-3p	0	0	0
hsa-miR-520h	0	0	0
hsa-miR-522-5p	0	0	0
hsa-miR-522-3p	0	0	0
hsa-miR-519a-5p	0	0	0
hsa-miR-519a-3p	0	0	0
hsa-miR-527	0	0	0
hsa-miR-516a-5p	0	0	0
hsa-miR-516a-3p	0	0	0
hsa-miR-499a-5p	0	0	0
hsa-miR-499a-3p	0	0	0
hsa-miR-500a-5p	0	0	0
hsa-miR-500a-3p	0	0	0
hsa-miR-501-5p	0	0	0
hsa-miR-501-3p	0	0	0
hsa-miR-502-5p	0	0	0
hsa-miR-502-3p	0	0	0
hsa-miR-450a-2-3p	0	0	0
hsa-miR-503-5p	0	0	0
hsa-miR-503-3p	0	0	0
hsa-miR-504-5p	0	0	0

hsa-miR-504-3p	0	0	0
hsa-miR-505-5p	0	0	0
hsa-miR-505-3p	0	0	0
hsa-miR-513a-5p	0	0	0
hsa-miR-513a-3p	0	0	0
hsa-miR-506-5p	0	0	0
hsa-miR-506-3p	0	0	0
hsa-miR-507	0	0	0
hsa-miR-508-5p	0	0	0
hsa-miR-508-3p	0	0	0
hsa-miR-509-5p	0	0	0
hsa-miR-509-3p	0	0	0
hsa-miR-510-5p	0	0	0
hsa-miR-510-3p	0	0	0
hsa-miR-514a-5p	0	0	0
hsa-miR-514a-3p	0	0	0
hsa-miR-532-5p	0	0	0
hsa-miR-532-3p	0	0	0
hsa-miR-455-5p	0	0	0
hsa-miR-455-3p	0	0	0
hsa-miR-539-5p	0	0	0
hsa-miR-539-3p	0	0	0
hsa-miR-544a	0	0	0
hsa-miR-545-5p	0	0	0
hsa-miR-545-3p	0	0	0
hsa-miR-376a-2-5p	0	0	0
hsa-miR-487b-5p	0	0	0
hsa-miR-487b-3p	0	0	0
hsa-miR-551a	0	0	0
hsa-miR-552-5p	0	0	0
hsa-miR-552-3p	0	0	0
hsa-miR-553	0	0	0
hsa-miR-554	0	0	0
hsa-miR-92b-5p	0	0	0
hsa-miR-92b-3p	0	0	0
hsa-miR-555	0	0	0
hsa-miR-556-5p	0	0	0
hsa-miR-556-3p	0	0	0
hsa-miR-557	0	0	0
hsa-miR-558	0	0	0
hsa-miR-559	0	0	0
hsa-miR-561-5p	0	0	0
hsa-miR-561-3p	0	0	0
hsa-miR-562	0	0	0
hsa-miR-563	0	0	0
hsa-miR-564	0	0	0
hsa-miR-566	0	0	0
hsa-miR-567	0	0	0
hsa-miR-568	0	0	0
hsa-miR-551b-5p	0	0	0
hsa-miR-551b-3p	0	0	0
hsa-miR-569	0	0	0

hsa-miR-570-5p	0	0	0
hsa-miR-570-3p	0	0	0
hsa-miR-571	0	0	0
hsa-miR-572	0	0	0
hsa-miR-573	0	0	0
hsa-miR-574-5p	0	0	0
hsa-miR-574-3p	0	0	0
hsa-miR-575	0	0	0
hsa-miR-576-5p	0	0	0
hsa-miR-576-3p	0	0	0
hsa-miR-577	0	0	0
hsa-miR-578	0	0	0
hsa-miR-579-5p	0	0	0
hsa-miR-579-3p	0	0	0
hsa-miR-580-5p	0	0	0
hsa-miR-580-3p	0	0	0
hsa-miR-581	0	0	0
hsa-miR-582-5p	0	0	0
hsa-miR-582-3p	0	0	0
hsa-miR-583	0	0	0
hsa-miR-584-5p	0	0	0
hsa-miR-584-3p	0	0	0
hsa-miR-585-5p	0	0	0
hsa-miR-585-3p	0	0	0
hsa-miR-548a-3p	0	0	0
hsa-miR-586	0	0	0
hsa-miR-587	0	0	0
hsa-miR-548b-5p	0	0	0
hsa-miR-548b-3p	0	0	0
hsa-miR-588	0	0	0
hsa-miR-589-5p	0	0	0
hsa-miR-589-3p	0	0	0
hsa-miR-550a-5p	0	0	0
hsa-miR-550a-3p	0	0	0
hsa-miR-590-5p	0	0	0
hsa-miR-590-3p	0	0	0
hsa-miR-591	0	0	0
hsa-miR-592	0	0	0
hsa-miR-593-5p	0	0	0
hsa-miR-593-3p	0	0	0
hsa-miR-595	0	0	0
hsa-miR-596	0	0	0
hsa-miR-597-5p	0	0	0
hsa-miR-597-3p	0	0	0
hsa-miR-598-5p	0	0	0
hsa-miR-598-3p	0	0	0
hsa-miR-599	0	0	0
hsa-miR-548a-5p	0	0	0
hsa-miR-600	0	0	0
hsa-miR-601	0	0	0
hsa-miR-602	0	0	0
hsa-miR-603	0	0	0

hsa-miR-604	0	0	0
hsa-miR-605-5p	0	0	0
hsa-miR-605-3p	0	0	0
hsa-miR-606	0	0	0
hsa-miR-607	0	0	0
hsa-miR-608	0	0	0
hsa-miR-609	0	0	0
hsa-miR-610	0	0	0
hsa-miR-611	0	0	0
hsa-miR-612	0	0	0
hsa-miR-613	0	0	0
hsa-miR-614	0	0	0
hsa-miR-615-5p	0	0	0
hsa-miR-615-3p	0	0	0
hsa-miR-616-5p	0	0	0
hsa-miR-616-3p	0	0	0
hsa-miR-548c-5p	0	0	0
hsa-miR-548c-3p	0	0	0
hsa-miR-617	0	0	0
hsa-miR-618	0	0	0
hsa-miR-619-5p	0	0	0
hsa-miR-619-3p	0	0	0
hsa-miR-620	0	0	0
hsa-miR-621	0	0	0
hsa-miR-622	0	0	0
hsa-miR-623	0	0	0
hsa-miR-624-5p	0	0	0
hsa-miR-624-3p	0	0	0
hsa-miR-625-5p	0	0	0
hsa-miR-625-3p	0	0	0
hsa-miR-626	0	0	0
hsa-miR-627-5p	0	0	0
hsa-miR-627-3p	0	0	0
hsa-miR-628-5p	0	0	0
hsa-miR-628-3p	0	0	0
hsa-miR-629-5p	0	0	0
hsa-miR-629-3p	0	0	0
hsa-miR-630	0	0	0
hsa-miR-631	0	0	0
hsa-miR-33b-5p	0	0	0
hsa-miR-33b-3p	0	0	0
hsa-miR-632	0	0	0
hsa-miR-633	0	0	0
hsa-miR-634	0	0	0
hsa-miR-635	0	0	0
hsa-miR-636	0	0	0
hsa-miR-637	0	0	0
hsa-miR-638	0	0	0
hsa-miR-639	0	0	0
hsa-miR-640	0	0	0
hsa-miR-641	0	0	0
hsa-miR-642a-5p	0	0	0

hsa-miR-642a-3p	0	0	0
hsa-miR-643	0	0	0
hsa-miR-644a	0	0	0
hsa-miR-645	0	0	0
hsa-miR-646	0	0	0
hsa-miR-647	0	0	0
hsa-miR-648	0	0	0
hsa-miR-649	0	0	0
hsa-miR-650	0	0	0
hsa-miR-651-5p	0	0	0
hsa-miR-651-3p	0	0	0
hsa-miR-652-5p	0	0	0
hsa-miR-652-3p	0	0	0
hsa-miR-548d-5p	0	0	0
hsa-miR-548d-3p	0	0	0
hsa-miR-661	0	0	0
hsa-miR-662	0	0	0
hsa-miR-663a	0	0	0
hsa-miR-449b-5p	0	0	0
hsa-miR-449b-3p	0	0	0
hsa-miR-653-5p	0	0	0
hsa-miR-653-3p	0	0	0
hsa-miR-411-5p	0	0	0
hsa-miR-411-3p	0	0	0
hsa-miR-654-5p	0	0	0
hsa-miR-654-3p	0	0	0
hsa-miR-655-5p	0	0	0
hsa-miR-655-3p	0	0	0
hsa-miR-656-5p	0	0	0
hsa-miR-656-3p	0	0	0
hsa-miR-549a	0	0	0
hsa-miR-657	0	0	0
hsa-miR-658	0	0	0
hsa-miR-659-5p	0	0	0
hsa-miR-659-3p	0	0	0
hsa-miR-660-5p	0	0	0
hsa-miR-660-3p	0	0	0
hsa-miR-421	0	0	0
hsa-miR-542-5p	0	0	0
hsa-miR-542-3p	0	0	0
hsa-miR-758-5p	0	0	0
hsa-miR-758-3p	0	0	0
hsa-miR-1264	0	0	0
hsa-miR-671-5p	0	0	0
hsa-miR-671-3p	0	0	0
hsa-miR-668-5p	0	0	0
hsa-miR-668-3p	0	0	0
hsa-miR-550a-3-5p	0	0	0
hsa-miR-767-5p	0	0	0
hsa-miR-767-3p	0	0	0
hsa-miR-1224-5p	0	0	0
hsa-miR-1224-3p	0	0	0

hsa-miR-151b	0	0	0
hsa-miR-320b	0	0	0
hsa-miR-320c	0	0	0
hsa-miR-1296-5p	0	0	0
hsa-miR-1296-3p	0	0	0
hsa-miR-1468-5p	0	0	0
hsa-miR-1468-3p	0	0	0
hsa-miR-1323	0	0	0
hsa-miR-1271-5p	0	0	0
hsa-miR-1271-3p	0	0	0
hsa-miR-1301-5p	0	0	0
hsa-miR-1301-3p	0	0	0
hsa-miR-454-5p	0	0	0
hsa-miR-454-3p	0	0	0
hsa-miR-1185-5p	0	0	0
hsa-miR-1185-2-3p	0	0	0
hsa-miR-449c-5p	0	0	0
hsa-miR-449c-3p	0	0	0
hsa-miR-1283	0	0	0
hsa-miR-769-5p	0	0	0
hsa-miR-769-3p	0	0	0
hsa-miR-766-5p	0	0	0
hsa-miR-766-3p	0	0	0
hsa-miR-378d	0	0	0
hsa-miR-1185-1-3p	0	0	0
hsa-miR-762	0	0	0
hsa-miR-802	0	0	0
hsa-miR-670-5p	0	0	0
hsa-miR-670-3p	0	0	0
hsa-miR-1298-5p	0	0	0
hsa-miR-1298-3p	0	0	0
hsa-miR-2113	0	0	0
hsa-miR-761	0	0	0
hsa-miR-764	0	0	0
hsa-miR-759	0	0	0
hsa-miR-765	0	0	0
hsa-miR-770-5p	0	0	0
hsa-miR-675-5p	0	0	0
hsa-miR-675-3p	0	0	0
hsa-miR-298	0	0	0
hsa-miR-891a-5p	0	0	0
hsa-miR-891a-3p	0	0	0
hsa-miR-300	0	0	0
hsa-miR-892a	0	0	0
hsa-miR-450b-5p	0	0	0
hsa-miR-450b-3p	0	0	0
hsa-miR-874-5p	0	0	0
hsa-miR-874-3p	0	0	0
hsa-miR-890	0	0	0
hsa-miR-891b	0	0	0
hsa-miR-888-5p	0	0	0
hsa-miR-888-3p	0	0	0

hsa-miR-892b	0	0	0
hsa-miR-541-5p	0	0	0
hsa-miR-541-3p	0	0	0
hsa-miR-889-5p	0	0	0
hsa-miR-889-3p	0	0	0
hsa-miR-875-5p	0	0	0
hsa-miR-875-3p	0	0	0
hsa-miR-876-5p	0	0	0
hsa-miR-876-3p	0	0	0
hsa-miR-708-5p	0	0	0
hsa-miR-708-3p	0	0	0
hsa-miR-147b	0	0	0
hsa-miR-190b	0	0	0
hsa-miR-744-5p	0	0	0
hsa-miR-744-3p	0	0	0
hsa-miR-885-5p	0	0	0
hsa-miR-885-3p	0	0	0
hsa-miR-877-5p	0	0	0
hsa-miR-877-3p	0	0	0
hsa-miR-887-5p	0	0	0
hsa-miR-887-3p	0	0	0
hsa-miR-665	0	0	0
hsa-miR-873-5p	0	0	0
hsa-miR-873-3p	0	0	0
hsa-miR-543	0	0	0
hsa-miR-374b-5p	0	0	0
hsa-miR-374b-3p	0	0	0
hsa-miR-760	0	0	0
hsa-miR-301b	0	0	0
hsa-miR-216b-5p	0	0	0
hsa-miR-216b-3p	0	0	0
hsa-miR-208b-5p	0	0	0
hsa-miR-208b-3p	0	0	0
hsa-miR-920	0	0	0
hsa-miR-921	0	0	0
hsa-miR-922	0	0	0
hsa-miR-924	0	0	0
hsa-miR-509-3-5p	0	0	0
hsa-miR-933	0	0	0
hsa-miR-934	0	0	0
hsa-miR-935	0	0	0
hsa-miR-936	0	0	0
hsa-miR-937-5p	0	0	0
hsa-miR-937-3p	0	0	0
hsa-miR-938	0	0	0
hsa-miR-939-5p	0	0	0
hsa-miR-939-3p	0	0	0
hsa-miR-940	0	0	0
hsa-miR-941	0	0	0
hsa-miR-942-5p	0	0	0
hsa-miR-942-3p	0	0	0
hsa-miR-943	0	0	0

hsa-miR-944	0	0	0
hsa-miR-297	0	0	0
hsa-miR-1178-5p	0	0	0
hsa-miR-1178-3p	0	0	0
hsa-miR-1179	0	0	0
hsa-miR-1180-5p	0	0	0
hsa-miR-1180-3p	0	0	0
hsa-miR-1181	0	0	0
hsa-miR-1182	0	0	0
hsa-miR-1183	0	0	0
hsa-miR-1184	0	0	0
hsa-miR-1225-5p	0	0	0
hsa-miR-1225-3p	0	0	0
hsa-miR-1226-5p	0	0	0
hsa-miR-1226-3p	0	0	0
hsa-miR-1227-5p	0	0	0
hsa-miR-1227-3p	0	0	0
hsa-miR-1228-5p	0	0	0
hsa-miR-1228-3p	0	0	0
hsa-miR-1229-5p	0	0	0
hsa-miR-1229-3p	0	0	0
hsa-miR-1231	0	0	0
hsa-miR-1233-5p	0	0	0
hsa-miR-1233-3p	0	0	0
hsa-miR-1234-3p	0	0	0
hsa-miR-1236-5p	0	0	0
hsa-miR-1236-3p	0	0	0
hsa-miR-1237-5p	0	0	0
hsa-miR-1237-3p	0	0	0
hsa-miR-1238-5p	0	0	0
hsa-miR-1238-3p	0	0	0
hsa-miR-1200	0	0	0
hsa-miR-1202	0	0	0
hsa-miR-1203	0	0	0
hsa-miR-663b	0	0	0
hsa-miR-1204	0	0	0
hsa-miR-1205	0	0	0
hsa-miR-1206	0	0	0
hsa-miR-1207-5p	0	0	0
hsa-miR-1207-3p	0	0	0
hsa-miR-1208	0	0	0
hsa-miR-548e-5p	0	0	0
hsa-miR-548e-3p	0	0	0
hsa-miR-548j-5p	0	0	0
hsa-miR-548j-3p	0	0	0
hsa-miR-1285-5p	0	0	0
hsa-miR-1285-3p	0	0	0
hsa-miR-1286	0	0	0
hsa-miR-1287-5p	0	0	0
hsa-miR-1287-3p	0	0	0
hsa-miR-1289	0	0	0
hsa-miR-1290	0	0	0

hsa-miR-1291	0	0	0
hsa-miR-548k	0	0	0
hsa-miR-1293	0	0	0
hsa-miR-1294	0	0	0
hsa-miR-1295a	0	0	0
hsa-miR-1297	0	0	0
hsa-miR-1299	0	0	0
hsa-miR-548l	0	0	0
hsa-miR-1302	0	0	0
hsa-miR-1303	0	0	0
hsa-miR-1304-5p	0	0	0
hsa-miR-1304-3p	0	0	0
hsa-miR-1305	0	0	0
hsa-miR-1243	0	0	0
hsa-miR-548f-5p	0	0	0
hsa-miR-548f-3p	0	0	0
hsa-miR-1244	0	0	0
hsa-miR-1245a	0	0	0
hsa-miR-1246	0	0	0
hsa-miR-1247-5p	0	0	0
hsa-miR-1247-3p	0	0	0
hsa-miR-1248	0	0	0
hsa-miR-1249	0	0	0
hsa-miR-1250-5p	0	0	0
hsa-miR-1250-3p	0	0	0
hsa-miR-1251-5p	0	0	0
hsa-miR-1251-3p	0	0	0
hsa-miR-1253	0	0	0
hsa-miR-1254	0	0	0
hsa-miR-1255a	0	0	0
hsa-miR-1256	0	0	0
hsa-miR-1257	0	0	0
hsa-miR-1258	0	0	0
hsa-miR-1260a	0	0	0
hsa-miR-548g-5p	0	0	0
hsa-miR-548g-3p	0	0	0
hsa-miR-1261	0	0	0
hsa-miR-1262	0	0	0
hsa-miR-1263	0	0	0
hsa-miR-548n	0	0	0
hsa-miR-548m	0	0	0
hsa-miR-1265	0	0	0
hsa-miR-548o-3p	0	0	0
hsa-miR-1266-5p	0	0	0
hsa-miR-1266-3p	0	0	0
hsa-miR-1267	0	0	0
hsa-miR-1268a	0	0	0
hsa-miR-1269a	0	0	0
hsa-miR-1270	0	0	0
hsa-miR-1272	0	0	0
hsa-miR-1273a	0	0	0
hsa-miR-548h-5p	0	0	0

hsa-miR-548h-3p	0	0	0
hsa-miR-1275	0	0	0
hsa-miR-1276	0	0	0
hsa-miR-302e	0	0	0
hsa-miR-302f	0	0	0
hsa-miR-1277-5p	0	0	0
hsa-miR-1277-3p	0	0	0
hsa-miR-548p	0	0	0
hsa-miR-548i	0	0	0
hsa-miR-1278	0	0	0
hsa-miR-1279	0	0	0
hsa-miR-1281	0	0	0
hsa-miR-1282	0	0	0
hsa-miR-1284	0	0	0
hsa-miR-1288-5p	0	0	0
hsa-miR-1288-3p	0	0	0
hsa-miR-1292-5p	0	0	0
hsa-miR-1292-3p	0	0	0
hsa-miR-1252-5p	0	0	0
hsa-miR-1252-3p	0	0	0
hsa-miR-1255b-5p	0	0	0
hsa-miR-1255b-2-3p	0	0	0
hsa-miR-664a-5p	0	0	0
hsa-miR-664a-3p	0	0	0
hsa-miR-1306-5p	0	0	0
hsa-miR-1306-3p	0	0	0
hsa-miR-1307-5p	0	0	0
hsa-miR-1307-3p	0	0	0
hsa-miR-513b-5p	0	0	0
hsa-miR-513b-3p	0	0	0
hsa-miR-513c-5p	0	0	0
hsa-miR-513c-3p	0	0	0
hsa-miR-1321	0	0	0
hsa-miR-1322	0	0	0
hsa-miR-1197	0	0	0
hsa-miR-1324	0	0	0
hsa-miR-1469	0	0	0
hsa-miR-1470	0	0	0
hsa-miR-1471	0	0	0
hsa-miR-1537-5p	0	0	0
hsa-miR-1537-3p	0	0	0
hsa-miR-1538	0	0	0
hsa-miR-1539	0	0	0
hsa-miR-103b	0	0	0
hsa-miR-320d	0	0	0
hsa-miR-1825	0	0	0
hsa-miR-1827	0	0	0
hsa-miR-1908-5p	0	0	0
hsa-miR-1908-3p	0	0	0
hsa-miR-1909-5p	0	0	0
hsa-miR-1909-3p	0	0	0
hsa-miR-1910-5p	0	0	0

hsa-miR-1910-3p	0	0	0
hsa-miR-1911-5p	0	0	0
hsa-miR-1911-3p	0	0	0
hsa-miR-1912	0	0	0
hsa-miR-1913	0	0	0
hsa-miR-1914-5p	0	0	0
hsa-miR-1914-3p	0	0	0
hsa-miR-1915-5p	0	0	0
hsa-miR-1915-3p	0	0	0
hsa-miR-1972	0	0	0
hsa-miR-1973	0	0	0
hsa-miR-1976	0	0	0
hsa-miR-2052	0	0	0
hsa-miR-2053	0	0	0
hsa-miR-2054	0	0	0
hsa-miR-2110	0	0	0
hsa-miR-2114-5p	0	0	0
hsa-miR-2114-3p	0	0	0
hsa-miR-2115-5p	0	0	0
hsa-miR-2115-3p	0	0	0
hsa-miR-2116-5p	0	0	0
hsa-miR-2116-3p	0	0	0
hsa-miR-2117	0	0	0
hsa-miR-548q	0	0	0
hsa-miR-2276-5p	0	0	0
hsa-miR-2276-3p	0	0	0
hsa-miR-2277-5p	0	0	0
hsa-miR-2277-3p	0	0	0
hsa-miR-2278	1	0	0
hsa-miR-2681-5p	0	0	0
hsa-miR-2681-3p	0	0	0
hsa-miR-2682-5p	0	0	0
hsa-miR-2682-3p	0	0	0
hsa-miR-711	0	0	0
hsa-miR-718	0	0	0
hsa-miR-2861	0	0	0
hsa-miR-2909	0	0	0
hsa-miR-3115	0	0	0
hsa-miR-3116	0	0	0
hsa-miR-3117-5p	0	0	0
hsa-miR-3117-3p	0	0	0
hsa-miR-3118	0	0	0
hsa-miR-3119	0	0	0
hsa-miR-3120-5p	0	0	0
hsa-miR-3120-3p	0	0	0
hsa-miR-3121-5p	0	0	0
hsa-miR-3121-3p	0	0	0
hsa-miR-3122	0	0	0
hsa-miR-3123	0	0	0
hsa-miR-3124-5p	0	0	0
hsa-miR-3124-3p	0	0	0
hsa-miR-548s	0	0	0

hsa-miR-3125	0	0	0
hsa-miR-3126-5p	0	0	0
hsa-miR-3126-3p	0	0	0
hsa-miR-3127-5p	0	0	0
hsa-miR-3127-3p	0	0	0
hsa-miR-3128	0	0	0
hsa-miR-3129-5p	0	0	0
hsa-miR-3129-3p	0	0	0
hsa-miR-3130-5p	0	0	0
hsa-miR-3130-3p	0	0	0
hsa-miR-3131	0	0	0
hsa-miR-3132	0	0	0
hsa-miR-3133	0	0	0
hsa-miR-378b	0	0	0
hsa-miR-3134	0	0	0
hsa-miR-3135a	0	0	0
hsa-miR-466	0	0	0
hsa-miR-3136-5p	0	0	0
hsa-miR-3136-3p	0	0	0
hsa-miR-544b	0	0	0
hsa-miR-3137	0	0	0
hsa-miR-3138	0	0	0
hsa-miR-3139	0	0	0
hsa-miR-3140-5p	0	0	0
hsa-miR-3140-3p	0	0	0
hsa-miR-548t-5p	0	0	0
hsa-miR-548t-3p	0	0	0
hsa-miR-3141	0	0	0
hsa-miR-3142	0	0	0
hsa-miR-3143	0	0	0
hsa-miR-548u	0	0	0
hsa-miR-3144-5p	0	0	0
hsa-miR-3144-3p	0	0	0
hsa-miR-3145-5p	0	0	0
hsa-miR-3145-3p	0	0	0
hsa-miR-1273c	0	0	0
hsa-miR-3146	0	0	0
hsa-miR-3147	0	0	0
hsa-miR-548v	0	0	0
hsa-miR-3148	0	0	0
hsa-miR-3149	0	0	0
hsa-miR-3150a-5p	0	0	0
hsa-miR-3150a-3p	0	0	0
hsa-miR-3151-5p	0	0	0
hsa-miR-3151-3p	0	0	0
hsa-miR-3152-5p	0	0	0
hsa-miR-3152-3p	0	0	0
hsa-miR-3153	0	0	0
hsa-miR-3074-5p	0	0	0
hsa-miR-3074-3p	0	0	0
hsa-miR-3154	0	0	0
hsa-miR-3155a	0	0	0

hsa-miR-3156-5p	0	0	0
hsa-miR-3156-3p	0	0	0
hsa-miR-3157-5p	0	0	0
hsa-miR-3157-3p	0	0	0
hsa-miR-3158-5p	0	0	0
hsa-miR-3158-3p	0	0	0
hsa-miR-3159	0	0	0
hsa-miR-3160-5p	0	0	0
hsa-miR-3160-3p	0	0	0
hsa-miR-3161	0	0	0
hsa-miR-3162-5p	0	0	0
hsa-miR-3162-3p	0	0	0
hsa-miR-3163	0	0	0
hsa-miR-3164	0	0	0
hsa-miR-3165	0	0	0
hsa-miR-3166	0	0	0
hsa-miR-1260b	0	0	0
hsa-miR-3167	0	0	0
hsa-miR-3168	0	0	0
hsa-miR-3169	0	0	0
hsa-miR-3170	0	0	0
hsa-miR-3171	0	0	0
hsa-miR-3173-5p	0	0	0
hsa-miR-3173-3p	0	0	0
hsa-miR-1193	0	0	0
hsa-miR-323b-5p	0	0	0
hsa-miR-323b-3p	0	0	0
hsa-miR-3174	0	0	0
hsa-miR-3175	0	0	0
hsa-miR-3176	0	0	0
hsa-miR-3177-5p	0	0	0
hsa-miR-3177-3p	0	0	0
hsa-miR-3178	0	0	0
hsa-miR-3179	0	0	0
hsa-miR-3180-5p	0	0	0
hsa-miR-3180-3p	0	0	0
hsa-miR-548w	0	0	0
hsa-miR-3181	0	0	0
hsa-miR-3182	0	0	0
hsa-miR-3183	0	0	0
hsa-miR-3184-5p	0	0	0
hsa-miR-3184-3p	0	0	0
hsa-miR-3185	0	0	0
hsa-miR-3065-5p	0	0	0
hsa-miR-3065-3p	0	0	0
hsa-miR-3186-5p	0	0	0
hsa-miR-3186-3p	0	0	0
hsa-miR-3187-5p	0	0	0
hsa-miR-3187-3p	0	0	0
hsa-miR-3188	0	0	0
hsa-miR-3189-5p	0	0	0
hsa-miR-3189-3p	0	0	0

hsa-miR-320e	0	0	0
hsa-miR-3190-5p	0	0	0
hsa-miR-3190-3p	0	0	0
hsa-miR-3191-5p	0	0	0
hsa-miR-3191-3p	0	0	0
hsa-miR-3192-5p	0	0	0
hsa-miR-3192-3p	0	0	0
hsa-miR-3193	0	0	0
hsa-miR-3194-5p	0	0	0
hsa-miR-3194-3p	0	0	0
hsa-miR-3195	0	0	0
hsa-miR-3196	0	0	0
hsa-miR-548x-5p	0	0	0
hsa-miR-548x-3p	0	0	0
hsa-miR-3197	0	0	0
hsa-miR-3198	0	0	0
hsa-miR-3199	0	0	0
hsa-miR-3200-5p	0	0	0
hsa-miR-3200-3p	0	0	0
hsa-miR-3201	0	0	0
hsa-miR-514b-5p	0	0	0
hsa-miR-514b-3p	0	0	0
hsa-miR-3202	0	0	0
hsa-miR-1273d	0	0	0
hsa-miR-4295	0	0	0
hsa-miR-4296	0	0	0
hsa-miR-4297	0	0	0
hsa-miR-378c	0	0	0
hsa-miR-4293	0	0	0
hsa-miR-4294	0	0	0
hsa-miR-4301	0	0	0
hsa-miR-4299	0	0	0
hsa-miR-4298	0	0	0
hsa-miR-4300	0	0	0
hsa-miR-4304	0	0	0
hsa-miR-4302	0	0	0
hsa-miR-4303	0	0	0
hsa-miR-4305	0	0	0
hsa-miR-4306	0	0	0
hsa-miR-4309	0	0	0
hsa-miR-4307	0	0	0
hsa-miR-4308	0	0	0
hsa-miR-4310	0	0	0
hsa-miR-4311	0	0	0
hsa-miR-4312	0	0	0
hsa-miR-4313	0	0	0
hsa-miR-4315	0	0	0
hsa-miR-4316	0	0	0
hsa-miR-4314	0	0	0
hsa-miR-4318	0	0	0
hsa-miR-4319	0	0	0
hsa-miR-4320	0	0	0

hsa-miR-4317	0	0	0
hsa-miR-4322	0	0	0
hsa-miR-4321	0	0	0
hsa-miR-4323	0	0	0
hsa-miR-4324	0	0	0
hsa-miR-4256	0	0	0
hsa-miR-4257	0	0	0
hsa-miR-4258	0	0	0
hsa-miR-4259	0	0	0
hsa-miR-4260	0	0	0
hsa-miR-4253	0	0	0
hsa-miR-4251	0	0	0
hsa-miR-4254	0	0	0
hsa-miR-4255	0	0	0
hsa-miR-4252	0	0	0
hsa-miR-4325	0	0	0
hsa-miR-4326	0	0	0
hsa-miR-4327	0	0	0
hsa-miR-4261	0	0	0
hsa-miR-4265	0	0	0
hsa-miR-4266	0	0	0
hsa-miR-4267	0	0	0
hsa-miR-4262	0	0	0
hsa-miR-2355-5p	0	0	0
hsa-miR-2355-3p	0	0	0
hsa-miR-4268	0	0	0
hsa-miR-4269	0	0	0
hsa-miR-4263	0	0	0
hsa-miR-4264	0	0	0
hsa-miR-4270	0	0	0
hsa-miR-4271	0	0	0
hsa-miR-4272	0	0	0
hsa-miR-4273	0	0	0
hsa-miR-4276	0	0	0
hsa-miR-4275	0	0	0
hsa-miR-4274	0	0	0
hsa-miR-4281	0	0	0
hsa-miR-4277	0	0	0
hsa-miR-4279	0	0	0
hsa-miR-4278	0	0	0
hsa-miR-4280	0	0	0
hsa-miR-4282	0	0	0
hsa-miR-4285	0	0	0
hsa-miR-4283	0	0	0
hsa-miR-4284	0	0	0
hsa-miR-4286	0	0	0
hsa-miR-4287	0	0	0
hsa-miR-4288	0	0	0
hsa-miR-4292	0	0	0
hsa-miR-4289	0	0	0
hsa-miR-4290	0	0	0
hsa-miR-4291	0	0	0

hsa-miR-4329	0	0	0
hsa-miR-4330	0	0	0
hsa-miR-500b-5p	0	0	0
hsa-miR-500b-3p	0	0	0
hsa-miR-4328	0	0	0
hsa-miR-3605-5p	0	0	0
hsa-miR-3605-3p	0	0	0
hsa-miR-3606-5p	0	0	0
hsa-miR-3606-3p	0	0	0
hsa-miR-3607-5p	0	0	0
hsa-miR-3607-3p	0	0	0
hsa-miR-3609	0	0	0
hsa-miR-3610	0	0	0
hsa-miR-3611	0	0	0
hsa-miR-3612	0	0	0
hsa-miR-3613-5p	0	0	0
hsa-miR-3613-3p	0	0	0
hsa-miR-3614-5p	0	0	0
hsa-miR-3614-3p	0	0	0
hsa-miR-3615	0	0	0
hsa-miR-3616-5p	0	0	0
hsa-miR-3616-3p	0	0	0
hsa-miR-3617-5p	1	0	0
hsa-miR-3617-3p	0	0	0
hsa-miR-3618	0	0	0
hsa-miR-3619-5p	0	0	0
hsa-miR-3619-3p	0	0	0
hsa-miR-23c	0	0	0
hsa-miR-3620-5p	0	0	0
hsa-miR-3620-3p	0	0	0
hsa-miR-3621	0	0	0
hsa-miR-3622a-5p	0	0	0
hsa-miR-3622a-3p	0	0	0
hsa-miR-3622b-5p	0	0	0
hsa-miR-3622b-3p	0	0	0
hsa-miR-3646	0	0	0
hsa-miR-3648	0	0	0
hsa-miR-3649	0	0	0
hsa-miR-3650	0	0	0
hsa-miR-3651	0	0	0
hsa-miR-3652	0	0	0
hsa-miR-3653	0	0	0
hsa-miR-3654	0	0	0
hsa-miR-3655	0	0	0
hsa-miR-3656	0	0	0
hsa-miR-3657	0	0	0
hsa-miR-3658	0	0	0
hsa-miR-1273e	0	0	0
hsa-miR-3659	0	0	0
hsa-miR-3660	0	0	0
hsa-miR-3661	0	0	0
hsa-miR-3662	0	0	0

hsa-miR-3663-5p	0	0	0
hsa-miR-3663-3p	0	0	0
hsa-miR-3664-5p	0	0	0
hsa-miR-3664-3p	0	0	0
hsa-miR-3665	0	0	0
hsa-miR-3666	0	0	0
hsa-miR-3667-5p	0	0	0
hsa-miR-3667-3p	0	0	0
hsa-miR-3668	0	0	0
hsa-miR-3669	0	0	0
hsa-miR-3670	0	0	0
hsa-miR-3671	0	0	0
hsa-miR-3672	0	0	0
hsa-miR-3673	0	0	0
hsa-miR-3674	0	0	0
hsa-miR-3675-5p	0	0	0
hsa-miR-3675-3p	0	0	0
hsa-miR-3677-5p	0	0	0
hsa-miR-3677-3p	0	0	0
hsa-miR-3678-5p	0	0	0
hsa-miR-3678-3p	0	0	0
hsa-miR-3679-5p	0	0	0
hsa-miR-3679-3p	0	0	0
hsa-miR-3680-5p	0	0	0
hsa-miR-3680-3p	0	0	0
hsa-miR-3681-5p	0	0	0
hsa-miR-3681-3p	0	0	0
hsa-miR-3682-5p	0	0	0
hsa-miR-3682-3p	0	0	0
hsa-miR-3683	0	0	0
hsa-miR-3684	0	0	0
hsa-miR-3685	0	0	0
hsa-miR-3686	0	0	0
hsa-miR-3687	0	0	0
hsa-miR-3688-5p	0	0	0
hsa-miR-3688-3p	0	0	0
hsa-miR-3689a-5p	0	0	0
hsa-miR-3689a-3p	0	0	0
hsa-miR-3690	0	0	0
hsa-miR-3691-5p	0	0	0
hsa-miR-3691-3p	0	0	0
hsa-miR-3692-5p	0	0	0
hsa-miR-3692-3p	0	0	0
hsa-miR-3713	0	0	0
hsa-miR-3714	0	0	0
hsa-miR-3180	0	0	0
hsa-miR-3907	0	0	0
hsa-miR-3689b-5p	0	0	0
hsa-miR-3689b-3p	0	0	0
hsa-miR-3908	0	0	0
hsa-miR-3909	0	0	0
hsa-miR-3910	0	0	0

hsa-miR-3911	0	0	0
hsa-miR-3912-5p	0	0	0
hsa-miR-3912-3p	0	0	0
hsa-miR-3913-5p	0	0	0
hsa-miR-3913-3p	0	0	0
hsa-miR-3914	0	0	0
hsa-miR-3915	0	0	0
hsa-miR-3916	0	0	0
hsa-miR-3917	0	0	0
hsa-miR-3918	0	0	0
hsa-miR-3919	0	0	0
hsa-miR-3150b-5p	0	0	0
hsa-miR-3150b-3p	0	0	0
hsa-miR-3920	0	0	0
hsa-miR-3921	0	0	0
hsa-miR-3922-5p	0	0	0
hsa-miR-3922-3p	0	0	0
hsa-miR-3923	0	0	0
hsa-miR-3924	0	0	0
hsa-miR-3925-5p	0	0	0
hsa-miR-3925-3p	0	0	0
hsa-miR-3926	0	0	0
hsa-miR-3927-5p	0	0	0
hsa-miR-3927-3p	0	0	0
hsa-miR-676-5p	0	0	0
hsa-miR-676-3p	0	0	0
hsa-miR-3928-5p	0	0	0
hsa-miR-3928-3p	0	0	0
hsa-miR-3929	0	0	0
hsa-miR-3934-5p	0	0	0
hsa-miR-3934-3p	0	0	0
hsa-miR-3935	0	0	0
hsa-miR-3936	0	0	0
hsa-miR-3937	0	0	0
hsa-miR-3938	0	0	0
hsa-miR-548y	0	0	0
hsa-miR-3939	0	0	0
hsa-miR-3940-5p	0	0	0
hsa-miR-3940-3p	0	0	0
hsa-miR-3941	0	0	0
hsa-miR-3942-5p	0	0	0
hsa-miR-3942-3p	0	0	0
hsa-miR-3943	0	0	0
hsa-miR-3944-5p	0	0	0
hsa-miR-3944-3p	0	0	0
hsa-miR-3945	0	0	0
hsa-miR-374c-5p	0	0	0
hsa-miR-374c-3p	0	0	0
hsa-miR-642b-5p	0	0	0
hsa-miR-642b-3p	0	0	0
hsa-miR-550b-2-5p	0	0	0
hsa-miR-550b-3p	0	0	0

hsa-miR-548z	0	0	0
hsa-miR-548aa	0	0	0
hsa-miR-548o-5p	0	0	0
hsa-miR-1268b	0	0	0
hsa-miR-378e	0	0	0
hsa-miR-548ab	0	0	0
hsa-miR-4417	0	0	0
hsa-miR-4418	0	0	0
hsa-miR-4419a	0	0	0
hsa-miR-378f	0	0	0
hsa-miR-4420	0	0	0
hsa-miR-4421	0	0	0
hsa-miR-4422	0	0	0
hsa-miR-4423-5p	0	0	0
hsa-miR-4423-3p	0	0	0
hsa-miR-378g	0	0	0
hsa-miR-548ac	0	0	0
hsa-miR-4424	0	0	0
hsa-miR-4425	0	0	0
hsa-miR-4426	0	0	0
hsa-miR-4427	0	0	0
hsa-miR-4428	0	0	0
hsa-miR-4429	0	0	0
hsa-miR-4430	0	0	0
hsa-miR-548ad	0	0	0
hsa-miR-4431	0	0	0
hsa-miR-4432	0	0	0
hsa-miR-4433-5p	0	0	0
hsa-miR-4433-3p	0	0	0
hsa-miR-4434	0	0	0
hsa-miR-4435	0	0	0
hsa-miR-4436a	0	0	0
hsa-miR-4437	0	0	0
hsa-miR-548ae	0	0	0
hsa-miR-4438	0	0	0
hsa-miR-4439	0	0	0
hsa-miR-4440	0	0	0
hsa-miR-4441	0	0	0
hsa-miR-4442	0	0	0
hsa-miR-4443	0	0	0
hsa-miR-4444	0	0	0
hsa-miR-4445-5p	0	0	0
hsa-miR-4445-3p	0	0	0
hsa-miR-4446-5p	0	0	0
hsa-miR-4446-3p	0	0	0
hsa-miR-4447	0	0	0
hsa-miR-4448	0	0	0
hsa-miR-4449	0	0	0
hsa-miR-548ag	0	0	0
hsa-miR-4450	0	0	0
hsa-miR-548ah-5p	0	0	0
hsa-miR-548ah-3p	0	0	0

hsa-miR-4451	0	0	0
hsa-miR-4452	0	0	0
hsa-miR-4453	0	0	0
hsa-miR-4454	0	0	0
hsa-miR-4455	0	0	0
hsa-miR-4456	0	0	0
hsa-miR-4457	0	0	0
hsa-miR-4458	0	0	0
hsa-miR-4459	0	0	0
hsa-miR-4460	0	0	0
hsa-miR-4461	0	0	0
hsa-miR-378h	0	0	0
hsa-miR-3135b	0	0	0
hsa-miR-4462	0	0	0
hsa-miR-4463	0	0	0
hsa-miR-4464	0	0	0
hsa-miR-548ai	0	0	0
hsa-miR-548aj-3p	0	0	0
hsa-miR-548aj-5p	0	0	0
hsa-miR-4465	0	0	0
hsa-miR-4466	0	0	0
hsa-miR-4467	0	0	0
hsa-miR-4468	0	0	0
hsa-miR-4469	0	0	0
hsa-miR-4470	0	0	0
hsa-miR-4471	0	0	0
hsa-miR-4472	0	0	0
hsa-miR-4473	0	0	0
hsa-miR-4474-5p	0	0	0
hsa-miR-4474-3p	0	0	0
hsa-miR-4475	0	0	0
hsa-miR-4476	0	0	0
hsa-miR-4477a	0	0	0
hsa-miR-4477b	0	0	0
hsa-miR-4478	0	0	0
hsa-miR-3689c	0	0	0
hsa-miR-3689d	0	0	0
hsa-miR-3689e	0	0	0
hsa-miR-3689f	0	0	0
hsa-miR-4479	0	0	0
hsa-miR-3155b	0	0	0
hsa-miR-548ak	0	0	0
hsa-miR-4480	0	0	0
hsa-miR-4481	0	0	0
hsa-miR-4482-5p	0	0	0
hsa-miR-4482-3p	0	0	0
hsa-miR-4483	0	0	0
hsa-miR-4484	0	0	0
hsa-miR-4485	0	0	0
hsa-miR-4486	0	0	0
hsa-miR-4487	0	0	0
hsa-miR-4488	0	0	0

hsa-miR-4489	0	0	0
hsa-miR-548al	0	0	0
hsa-miR-4490	0	0	0
hsa-miR-4491	0	0	0
hsa-miR-4492	0	0	0
hsa-miR-4493	0	0	0
hsa-miR-4494	0	0	0
hsa-miR-4495	0	0	0
hsa-miR-4496	0	0	0
hsa-miR-4497	0	0	0
hsa-miR-4498	0	0	0
hsa-miR-4419b	0	0	0
hsa-miR-4499	0	0	0
hsa-miR-4500	0	0	0
hsa-miR-4501	0	0	0
hsa-miR-4502	0	0	0
hsa-miR-4503	0	0	0
hsa-miR-4504	0	0	0
hsa-miR-4505	0	0	0
hsa-miR-4506	0	0	0
hsa-miR-2392	0	0	0
hsa-miR-4507	0	0	0
hsa-miR-4508	0	0	0
hsa-miR-4509	0	0	0
hsa-miR-4510	0	0	0
hsa-miR-4511	0	0	0
hsa-miR-4512	0	0	0
hsa-miR-4513	0	0	0
hsa-miR-4514	0	0	0
hsa-miR-4515	0	0	0
hsa-miR-4516	0	0	0
hsa-miR-4517	0	0	0
hsa-miR-4518	0	0	0
hsa-miR-4519	0	0	0
hsa-miR-4520a-5p	0	0	0
hsa-miR-4520a-3p	0	0	0
hsa-miR-4521	1	0	1
hsa-miR-1269b	0	0	0
hsa-miR-4522	0	0	0
hsa-miR-4523	0	0	0
hsa-miR-4524a-5p	0	0	0
hsa-miR-4524a-3p	0	0	0
hsa-miR-4525	0	0	0
hsa-miR-4526	0	0	0
hsa-miR-4527	0	0	0
hsa-miR-4528	0	0	0
hsa-miR-4529-5p	0	0	0
hsa-miR-4529-3p	0	0	0
hsa-miR-4530	0	0	0
hsa-miR-4531	0	0	0
hsa-miR-4532	0	0	0
hsa-miR-4533	0	0	0

hsa-miR-4534	0	0	0
hsa-miR-378i	0	0	0
hsa-miR-4535	0	0	0
hsa-miR-548am-5p	0	0	0
hsa-miR-548am-3p	0	0	0
hsa-miR-1587	0	0	0
hsa-miR-4536-5p	0	0	0
hsa-miR-4536-3p	0	0	0
hsa-miR-548an	0	0	0
hsa-miR-4537	0	0	0
hsa-miR-4538	0	0	0
hsa-miR-4539	0	0	0
hsa-miR-4540	0	0	0
hsa-miR-3960	0	0	0
hsa-miR-3972	0	0	0
hsa-miR-3973	0	0	0
hsa-miR-3974	0	0	0
hsa-miR-3975	0	0	0
hsa-miR-3976	0	0	0
hsa-miR-3977	0	0	0
hsa-miR-3978	0	0	0
hsa-miR-4632-5p	0	0	0
hsa-miR-4632-3p	0	0	0
hsa-miR-4633-5p	0	0	0
hsa-miR-4633-3p	0	0	0
hsa-miR-4634	0	0	0
hsa-miR-4635	0	0	0
hsa-miR-4636	0	0	0
hsa-miR-4637	0	0	0
hsa-miR-4638-5p	0	0	0
hsa-miR-4638-3p	0	0	0
hsa-miR-4639-5p	0	0	0
hsa-miR-4639-3p	0	0	0
hsa-miR-4640-5p	0	0	0
hsa-miR-4640-3p	0	0	0
hsa-miR-4641	0	0	0
hsa-miR-4642	0	0	0
hsa-miR-4643	0	0	0
hsa-miR-4644	0	0	0
hsa-miR-4645-5p	0	0	0
hsa-miR-4645-3p	0	0	0
hsa-miR-4646-5p	0	0	0
hsa-miR-4646-3p	0	0	0
hsa-miR-4647	0	0	0
hsa-miR-4648	0	0	0
hsa-miR-4649-5p	0	0	0
hsa-miR-4649-3p	0	0	0
hsa-miR-4650-5p	0	0	0
hsa-miR-4650-3p	0	0	0
hsa-miR-4651	0	0	0
hsa-miR-4652-5p	0	0	0
hsa-miR-4652-3p	0	0	0

hsa-miR-4653-5p	0	0	0
hsa-miR-4653-3p	0	0	0
hsa-miR-4654	0	0	0
hsa-miR-4655-5p	0	0	0
hsa-miR-4655-3p	0	0	0
hsa-miR-4656	0	0	0
hsa-miR-4657	0	0	0
hsa-miR-4658	0	0	0
hsa-miR-4659a-5p	0	0	0
hsa-miR-4659a-3p	0	0	0
hsa-miR-4660	0	0	0
hsa-miR-4661-5p	0	0	0
hsa-miR-4661-3p	0	0	0
hsa-miR-4662a-5p	0	0	0
hsa-miR-4662a-3p	0	0	0
hsa-miR-4659b-5p	0	0	0
hsa-miR-4659b-3p	0	0	0
hsa-miR-4663	0	0	0
hsa-miR-4662b	0	0	0
hsa-miR-4664-5p	0	0	0
hsa-miR-4664-3p	0	0	0
hsa-miR-4665-5p	0	0	0
hsa-miR-4665-3p	0	0	0
hsa-miR-4666a-5p	0	0	0
hsa-miR-4666a-3p	0	0	0
hsa-miR-4667-5p	0	0	0
hsa-miR-4667-3p	0	0	0
hsa-miR-4668-5p	0	0	0
hsa-miR-4668-3p	0	0	0
hsa-miR-219b-5p	0	0	0
hsa-miR-219b-3p	0	0	0
hsa-miR-4669	0	0	0
hsa-miR-4670-5p	0	0	0
hsa-miR-4670-3p	0	0	0
hsa-miR-4671-5p	0	0	0
hsa-miR-4671-3p	0	0	0
hsa-miR-4672	0	0	0
hsa-miR-4673	0	0	0
hsa-miR-4674	0	0	0
hsa-miR-4675	0	0	0
hsa-miR-4676-5p	0	0	0
hsa-miR-4676-3p	0	0	0
hsa-miR-4677-5p	0	0	0
hsa-miR-4677-3p	0	0	0
hsa-miR-4678	0	0	0
hsa-miR-4679	0	0	0
hsa-miR-4680-5p	0	0	0
hsa-miR-4680-3p	0	0	0
hsa-miR-4681	0	0	0
hsa-miR-4682	0	0	0
hsa-miR-4683	0	0	0
hsa-miR-4684-5p	0	0	0

hsa-miR-4684-3p	0	0	0
hsa-miR-4685-5p	0	0	0
hsa-miR-4685-3p	0	0	0
hsa-miR-4686	0	0	0
hsa-miR-4687-5p	0	0	0
hsa-miR-4687-3p	0	0	0
hsa-miR-1343-5p	0	0	0
hsa-miR-1343-3p	0	0	0
hsa-miR-4688	0	0	0
hsa-miR-4689	0	0	0
hsa-miR-4690-5p	0	0	0
hsa-miR-4690-3p	0	0	0
hsa-miR-4691-5p	0	0	0
hsa-miR-4691-3p	0	0	0
hsa-miR-4692	0	0	0
hsa-miR-4693-5p	0	0	0
hsa-miR-4693-3p	0	0	0
hsa-miR-4694-5p	0	0	0
hsa-miR-4694-3p	0	0	0
hsa-miR-4695-5p	0	0	0
hsa-miR-4695-3p	0	0	0
hsa-miR-4696	0	0	0
hsa-miR-4697-5p	0	0	0
hsa-miR-4697-3p	0	0	0
hsa-miR-4698	0	0	0
hsa-miR-4699-5p	0	0	0
hsa-miR-4699-3p	0	0	0
hsa-miR-4700-5p	0	0	0
hsa-miR-4700-3p	0	0	0
hsa-miR-4701-5p	0	0	0
hsa-miR-4701-3p	0	0	0
hsa-miR-4703-5p	0	0	0
hsa-miR-4703-3p	0	0	0
hsa-miR-4704-5p	0	0	0
hsa-miR-4704-3p	0	0	0
hsa-miR-4705	0	0	0
hsa-miR-4706	0	0	0
hsa-miR-4707-5p	0	0	0
hsa-miR-4707-3p	0	0	0
hsa-miR-4708-5p	0	0	0
hsa-miR-4708-3p	0	0	0
hsa-miR-4709-5p	0	0	0
hsa-miR-4709-3p	0	0	0
hsa-miR-203b-5p	0	0	0
hsa-miR-203b-3p	0	0	0
hsa-miR-4710	0	0	0
hsa-miR-4711-5p	0	0	0
hsa-miR-4711-3p	0	0	0
hsa-miR-4712-5p	0	0	0
hsa-miR-4712-3p	0	0	0
hsa-miR-4713-5p	0	0	0
hsa-miR-4713-3p	0	0	0

hsa-miR-4714-5p	0	0	0
hsa-miR-4714-3p	0	0	0
hsa-miR-4715-5p	0	0	0
hsa-miR-4715-3p	0	0	0
hsa-miR-4716-5p	0	0	0
hsa-miR-4716-3p	0	0	0
hsa-miR-3529-5p	0	0	0
hsa-miR-3529-3p	0	0	0
hsa-miR-4717-5p	0	0	0
hsa-miR-4717-3p	0	0	0
hsa-miR-4718	0	0	0
hsa-miR-4719	0	0	0
hsa-miR-4720-5p	0	0	0
hsa-miR-4720-3p	0	0	0
hsa-miR-4721	0	0	0
hsa-miR-4722-5p	0	0	0
hsa-miR-4722-3p	0	0	0
hsa-miR-4520b-5p	0	0	0
hsa-miR-4520b-3p	0	0	0
hsa-miR-4723-5p	0	0	0
hsa-miR-4723-3p	0	0	0
hsa-miR-451b	0	0	0
hsa-miR-4724-5p	0	0	0
hsa-miR-4724-3p	0	0	0
hsa-miR-4725-5p	0	0	0
hsa-miR-4725-3p	0	0	0
hsa-miR-4726-5p	0	0	0
hsa-miR-4726-3p	0	0	0
hsa-miR-4727-5p	0	0	0
hsa-miR-4727-3p	0	0	0
hsa-miR-4728-5p	0	0	0
hsa-miR-4728-3p	0	0	0
hsa-miR-4729	0	0	0
hsa-miR-4730	0	0	0
hsa-miR-4731-5p	0	0	0
hsa-miR-4731-3p	0	0	0
hsa-miR-4732-5p	0	0	0
hsa-miR-4732-3p	0	0	0
hsa-miR-4733-5p	0	0	0
hsa-miR-4733-3p	0	0	0
hsa-miR-4734	0	0	0
hsa-miR-4735-5p	0	0	0
hsa-miR-4735-3p	0	0	0
hsa-miR-4736	0	0	0
hsa-miR-4737	1	0	1
hsa-miR-3064-5p	0	0	0
hsa-miR-3064-3p	0	0	0
hsa-miR-4738-5p	0	0	0
hsa-miR-4738-3p	0	0	0
hsa-miR-4739	0	0	0
hsa-miR-4740-5p	0	0	0
hsa-miR-4740-3p	0	0	0

hsa-miR-4741	0	0	0
hsa-miR-4742-5p	0	0	0
hsa-miR-4742-3p	0	0	0
hsa-miR-4743-5p	0	0	0
hsa-miR-4743-3p	0	0	0
hsa-miR-4744	0	0	0
hsa-miR-3591-5p	0	0	0
hsa-miR-3591-3p	0	0	0
hsa-miR-4745-5p	0	0	0
hsa-miR-4745-3p	0	0	0
hsa-miR-4746-5p	0	0	0
hsa-miR-4746-3p	0	0	0
hsa-miR-4747-5p	0	0	0
hsa-miR-4747-3p	0	0	0
hsa-miR-4748	0	0	0
hsa-miR-4749-5p	0	0	0
hsa-miR-4749-3p	0	0	0
hsa-miR-4750-5p	0	0	0
hsa-miR-4750-3p	0	0	0
hsa-miR-4751	0	0	0
hsa-miR-4752	0	0	0
hsa-miR-4753-5p	0	0	0
hsa-miR-4753-3p	0	0	0
hsa-miR-371b-5p	0	0	0
hsa-miR-371b-3p	0	0	0
hsa-miR-4754	0	0	0
hsa-miR-4755-5p	0	0	0
hsa-miR-4755-3p	0	0	0
hsa-miR-499b-5p	0	0	0
hsa-miR-499b-3p	0	0	0
hsa-miR-4756-5p	0	0	0
hsa-miR-4756-3p	0	0	0
hsa-miR-4757-5p	0	0	0
hsa-miR-4757-3p	0	0	0
hsa-miR-4758-5p	0	0	0
hsa-miR-4758-3p	0	0	0
hsa-miR-4759	0	0	0
hsa-miR-4760-5p	0	0	0
hsa-miR-4760-3p	0	0	0
hsa-miR-4761-5p	0	0	0
hsa-miR-4761-3p	0	0	0
hsa-miR-4762-5p	0	0	0
hsa-miR-4762-3p	0	0	0
hsa-miR-4763-5p	0	0	0
hsa-miR-4763-3p	0	0	0
hsa-miR-4764-5p	0	0	0
hsa-miR-4764-3p	0	0	0
hsa-miR-4765	0	0	0
hsa-miR-4766-5p	0	0	0
hsa-miR-4766-3p	0	0	0
hsa-miR-4767	0	0	0
hsa-miR-4768-5p	0	0	0

hsa-miR-4768-3p	0	0	0
hsa-miR-4769-5p	0	0	0
hsa-miR-4769-3p	0	0	0
hsa-miR-4770	0	0	0
hsa-miR-4771	0	0	0
hsa-miR-4772-5p	0	0	0
hsa-miR-4772-3p	0	0	0
hsa-miR-4773	0	0	0
hsa-miR-4774-5p	1	0	0
hsa-miR-4774-3p	0	0	0
hsa-miR-4775	0	0	0
hsa-miR-4776-5p	0	0	0
hsa-miR-4776-3p	0	0	0
hsa-miR-4777-5p	0	0	0
hsa-miR-4777-3p	0	0	0
hsa-miR-4778-5p	0	0	0
hsa-miR-4778-3p	0	0	0
hsa-miR-4779	0	0	0
hsa-miR-4780	0	0	0
hsa-miR-4436b-5p	0	0	0
hsa-miR-4436b-3p	0	0	0
hsa-miR-4781-5p	0	0	0
hsa-miR-4781-3p	0	0	0
hsa-miR-4782-5p	0	0	0
hsa-miR-4782-3p	0	0	0
hsa-miR-4783-5p	0	0	0
hsa-miR-4783-3p	0	0	0
hsa-miR-4784	1	0	0
hsa-miR-4785	0	0	0
hsa-miR-1245b-5p	0	0	0
hsa-miR-1245b-3p	0	0	0
hsa-miR-2467-5p	0	0	0
hsa-miR-2467-3p	0	0	0
hsa-miR-4786-5p	0	0	0
hsa-miR-4786-3p	0	0	0
hsa-miR-4787-5p	0	0	0
hsa-miR-4787-3p	0	0	0
hsa-miR-4788	0	0	0
hsa-miR-4789-5p	0	0	0
hsa-miR-4789-3p	0	0	0
hsa-miR-4790-5p	0	0	0
hsa-miR-4790-3p	0	0	0
hsa-miR-4791	0	0	0
hsa-miR-4792	0	0	0
hsa-miR-4793-5p	0	0	0
hsa-miR-4793-3p	0	0	0
hsa-miR-4794	0	0	0
hsa-miR-4795-5p	0	0	0
hsa-miR-4795-3p	0	0	0
hsa-miR-4796-5p	0	0	0
hsa-miR-4796-3p	0	0	0
hsa-miR-4797-5p	0	0	0

hsa-miR-4797-3p	0	0	0
hsa-miR-4798-5p	0	0	0
hsa-miR-4798-3p	0	0	0
hsa-miR-4799-5p	0	0	0
hsa-miR-4799-3p	0	0	0
hsa-miR-4800-5p	0	0	0
hsa-miR-4800-3p	0	0	0
hsa-miR-4801	0	0	0
hsa-miR-4802-5p	0	0	0
hsa-miR-4802-3p	0	0	0
hsa-miR-4803	0	0	0
hsa-miR-4804-5p	0	0	0
hsa-miR-4804-3p	0	0	0
hsa-miR-4999-5p	0	0	0
hsa-miR-4999-3p	0	0	0
hsa-miR-5000-5p	0	0	0
hsa-miR-5000-3p	0	0	0
hsa-miR-5001-5p	0	0	0
hsa-miR-5001-3p	0	0	0
hsa-miR-5002-5p	0	0	0
hsa-miR-5002-3p	0	0	0
hsa-miR-5003-5p	0	0	0
hsa-miR-5003-3p	0	0	0
hsa-miR-5004-5p	0	0	0
hsa-miR-5004-3p	0	0	0
hsa-miR-548ao-5p	0	0	0
hsa-miR-548ao-3p	0	0	0
hsa-miR-5006-5p	0	0	0
hsa-miR-5006-3p	0	0	0
hsa-miR-5007-5p	0	0	0
hsa-miR-5007-3p	0	0	0
hsa-miR-548ap-5p	0	0	0
hsa-miR-548ap-3p	0	0	0
hsa-miR-5008-5p	0	0	0
hsa-miR-5008-3p	0	0	0
hsa-miR-5009-5p	0	0	0
hsa-miR-5009-3p	0	0	0
hsa-miR-5010-5p	0	0	0
hsa-miR-5010-3p	0	0	0
hsa-miR-5011-5p	0	0	0
hsa-miR-5011-3p	0	0	0
hsa-miR-5047	0	0	0
hsa-miR-5087	0	0	0
hsa-miR-5088-5p	0	0	0
hsa-miR-5088-3p	0	0	0
hsa-miR-5089-5p	0	1	0
hsa-miR-5089-3p	0	0	0
hsa-miR-5090	0	0	0
hsa-miR-5091	0	0	0
hsa-miR-5092	0	0	0
hsa-miR-5093	0	0	0
hsa-miR-5094	0	0	0

hsa-miR-5095	0	0	0
hsa-miR-1273f	0	0	0
hsa-miR-1273g-5p	0	0	0
hsa-miR-1273g-3p	0	0	0
hsa-miR-5096	0	0	0
hsa-miR-5186	0	0	0
hsa-miR-5187-5p	0	0	0
hsa-miR-5187-3p	0	0	0
hsa-miR-5188	0	0	0
hsa-miR-5189-5p	0	0	0
hsa-miR-5189-3p	0	0	0
hsa-miR-5190	0	0	0
hsa-miR-5191	0	0	0
hsa-miR-5192	0	0	0
hsa-miR-5193	0	0	0
hsa-miR-5194	0	0	0
hsa-miR-5195-5p	0	0	0
hsa-miR-5195-3p	0	0	0
hsa-miR-5196-5p	0	0	0
hsa-miR-5196-3p	0	0	0
hsa-miR-5197-5p	0	0	0
hsa-miR-5197-3p	0	0	0
hsa-miR-4524b-5p	0	0	0
hsa-miR-4524b-3p	0	0	0
hsa-miR-5571-5p	0	0	0
hsa-miR-5571-3p	0	0	0
hsa-miR-5100	0	0	0
hsa-miR-5572	0	0	0
hsa-miR-548aq-5p	0	0	0
hsa-miR-548aq-3p	0	0	0
hsa-miR-548ar-5p	0	0	0
hsa-miR-548ar-3p	0	0	0
hsa-miR-548as-5p	0	0	0
hsa-miR-548as-3p	0	0	0
hsa-miR-5579-5p	0	0	0
hsa-miR-5579-3p	0	0	0
hsa-miR-664b-5p	0	0	0
hsa-miR-664b-3p	0	0	0
hsa-miR-5580-5p	0	0	0
hsa-miR-5580-3p	0	0	0
hsa-miR-5581-5p	0	0	0
hsa-miR-5581-3p	0	0	0
hsa-miR-548at-5p	0	0	0
hsa-miR-548at-3p	0	0	0
hsa-miR-5582-5p	0	0	0
hsa-miR-5582-3p	0	0	0
hsa-miR-5583-5p	0	0	0
hsa-miR-5583-3p	0	0	0
hsa-miR-5584-5p	0	0	0
hsa-miR-5584-3p	0	0	0
hsa-miR-5585-5p	0	0	0
hsa-miR-5585-3p	0	0	0

hsa-miR-5586-5p	0	0	0
hsa-miR-5586-3p	0	0	0
hsa-miR-5587-5p	0	0	0
hsa-miR-5587-3p	0	0	0
hsa-miR-548au-5p	0	0	0
hsa-miR-548au-3p	0	0	0
hsa-miR-1295b-5p	0	0	0
hsa-miR-1295b-3p	0	0	0
hsa-miR-5588-5p	0	0	0
hsa-miR-5588-3p	0	0	0
hsa-miR-5589-5p	0	0	0
hsa-miR-5589-3p	0	0	0
hsa-miR-5590-5p	0	0	0
hsa-miR-5590-3p	0	0	0
hsa-miR-5591-5p	0	0	0
hsa-miR-5591-3p	0	0	0
hsa-miR-548av-5p	0	0	0
hsa-miR-548av-3p	0	0	0
hsa-miR-5680	0	0	0
hsa-miR-5681a	0	0	0
hsa-miR-5682	0	0	0
hsa-miR-548aw	0	0	0
hsa-miR-5683	0	0	0
hsa-miR-5684	0	0	0
hsa-miR-548ax	0	0	0
hsa-miR-5685	0	0	0
hsa-miR-5692c	0	0	0
hsa-miR-5687	0	0	0
hsa-miR-5688	0	0	0
hsa-miR-5681b	0	0	0
hsa-miR-5689	0	0	0
hsa-miR-5690	0	0	0
hsa-miR-5691	0	0	0
hsa-miR-5692a	0	0	0
hsa-miR-4666b	0	0	0
hsa-miR-5693	0	0	0
hsa-miR-5694	0	0	0
hsa-miR-5695	0	0	0
hsa-miR-5696	0	0	0
hsa-miR-5697	0	0	0
hsa-miR-5698	0	0	0
hsa-miR-5699-5p	0	0	0
hsa-miR-5699-3p	0	0	0
hsa-miR-5700	0	0	0
hsa-miR-5701	0	0	0
hsa-miR-5702	0	0	0
hsa-miR-5703	0	0	0
hsa-miR-5692b	0	0	0
hsa-miR-5704	0	0	0
hsa-miR-5705	0	0	0
hsa-miR-5706	0	0	0
hsa-miR-5707	0	0	0

hsa-miR-5708	0	0	0
hsa-miR-5739	0	0	0
hsa-miR-5787	0	0	0
hsa-miR-1199-5p	0	0	0
hsa-miR-1199-3p	0	0	0
hsa-miR-6068	0	0	0
hsa-miR-6069	0	0	0
hsa-miR-6070	0	0	0
hsa-miR-6071	0	0	0
hsa-miR-6072	0	0	0
hsa-miR-6073	0	0	0
hsa-miR-6074	0	0	0
hsa-miR-6075	0	0	0
hsa-miR-6076	0	0	0
hsa-miR-6077	0	0	0
hsa-miR-6078	0	0	0
hsa-miR-6079	0	0	0
hsa-miR-6080	0	0	0
hsa-miR-6081	0	0	0
hsa-miR-6082	0	0	0
hsa-miR-6083	0	0	0
hsa-miR-6084	0	0	0
hsa-miR-6085	0	0	0
hsa-miR-6086	0	0	0
hsa-miR-6087	0	0	0
hsa-miR-6088	0	0	0
hsa-miR-6089	0	0	0
hsa-miR-6090	0	0	0
hsa-miR-6124	0	0	0
hsa-miR-6125	0	0	0
hsa-miR-6126	0	0	0
hsa-miR-6127	0	0	0
hsa-miR-6128	0	0	0
hsa-miR-378j	0	0	0
hsa-miR-6129	0	0	0
hsa-miR-6130	0	0	0
hsa-miR-6131	0	0	0
hsa-miR-6132	0	0	0
hsa-miR-6133	0	0	0
hsa-miR-6134	0	0	0
hsa-miR-6165	0	0	0
hsa-miR-6499-5p	0	0	0
hsa-miR-6499-3p	0	0	0
hsa-miR-548ay-5p	0	0	0
hsa-miR-548ay-3p	0	0	0
hsa-miR-6500-5p	0	0	0
hsa-miR-6500-3p	0	0	0
hsa-miR-548az-5p	0	0	0
hsa-miR-548az-3p	0	0	0
hsa-miR-6501-5p	0	0	0
hsa-miR-6501-3p	0	0	0
hsa-miR-6502-5p	0	0	0

hsa-miR-6502-3p	0	0	0
hsa-miR-6503-5p	0	0	0
hsa-miR-6503-3p	0	0	0
hsa-miR-6504-5p	0	0	0
hsa-miR-6504-3p	0	0	0
hsa-miR-6505-5p	0	0	0
hsa-miR-6505-3p	0	0	0
hsa-miR-6506-5p	0	0	0
hsa-miR-6506-3p	0	0	0
hsa-miR-6507-5p	0	0	0
hsa-miR-6507-3p	0	0	0
hsa-miR-6508-5p	0	0	0
hsa-miR-6508-3p	0	0	0
hsa-miR-6509-5p	0	0	0
hsa-miR-6509-3p	0	0	0
hsa-miR-6510-5p	0	0	0
hsa-miR-6510-3p	0	0	0
hsa-miR-6511a-5p	0	0	0
hsa-miR-6511a-3p	0	0	0
hsa-miR-6512-5p	0	0	0
hsa-miR-6512-3p	0	0	0
hsa-miR-6513-5p	0	0	0
hsa-miR-6513-3p	0	0	0
hsa-miR-6514-5p	0	0	0
hsa-miR-6514-3p	0	0	0
hsa-miR-6515-5p	0	0	0
hsa-miR-6515-3p	0	0	0
hsa-miR-6715a-3p	0	0	0
hsa-miR-6715b-5p	0	0	0
hsa-miR-6715b-3p	0	0	0
hsa-miR-6716-5p	0	0	0
hsa-miR-6716-3p	0	0	0
hsa-miR-6717-5p	0	0	0
hsa-miR-6511b-5p	0	0	0
hsa-miR-6511b-3p	0	0	0
hsa-miR-6718-5p	0	0	0
hsa-miR-6719-3p	0	0	0
hsa-miR-6720-5p	0	0	0
hsa-miR-6720-3p	0	0	0
hsa-miR-6721-5p	0	0	0
hsa-miR-6722-5p	0	0	0
hsa-miR-6722-3p	0	0	0
hsa-miR-6723-5p	0	0	0
hsa-miR-6724-5p	0	0	0
hsa-miR-892c-5p	0	0	0
hsa-miR-892c-3p	0	0	0
hsa-miR-6726-5p	0	0	0
hsa-miR-6726-3p	0	0	0
hsa-miR-6727-5p	0	0	0
hsa-miR-6727-3p	0	0	0
hsa-miR-6728-5p	0	0	0
hsa-miR-6728-3p	0	0	0

hsa-miR-6729-5p	0	0	0
hsa-miR-6729-3p	0	0	0
hsa-miR-6730-5p	0	0	0
hsa-miR-6730-3p	0	0	0
hsa-miR-6731-5p	0	0	0
hsa-miR-6731-3p	0	0	0
hsa-miR-6732-5p	0	0	0
hsa-miR-6732-3p	0	0	0
hsa-miR-6733-5p	0	0	0
hsa-miR-6733-3p	0	0	0
hsa-miR-6734-5p	0	0	0
hsa-miR-6734-3p	0	0	0
hsa-miR-6735-5p	0	0	0
hsa-miR-6735-3p	0	0	0
hsa-miR-6736-5p	0	0	0
hsa-miR-6736-3p	0	0	0
hsa-miR-6737-5p	0	0	0
hsa-miR-6737-3p	0	0	0
hsa-miR-6738-5p	0	0	0
hsa-miR-6738-3p	0	0	0
hsa-miR-6739-5p	0	0	0
hsa-miR-6739-3p	0	0	0
hsa-miR-6740-5p	0	0	0
hsa-miR-6740-3p	0	0	0
hsa-miR-6741-5p	0	0	0
hsa-miR-6741-3p	0	0	0
hsa-miR-6742-5p	0	0	0
hsa-miR-6742-3p	0	0	0
hsa-miR-6743-5p	0	0	0
hsa-miR-6743-3p	0	0	0
hsa-miR-6744-5p	0	0	0
hsa-miR-6744-3p	0	0	0
hsa-miR-6745	0	0	0
hsa-miR-6746-5p	0	0	0
hsa-miR-6746-3p	0	0	0
hsa-miR-6747-5p	0	0	0
hsa-miR-6747-3p	0	0	0
hsa-miR-6748-5p	0	0	0
hsa-miR-6748-3p	0	0	0
hsa-miR-6749-5p	0	0	0
hsa-miR-6749-3p	0	0	0
hsa-miR-6750-5p	0	0	0
hsa-miR-6750-3p	0	0	0
hsa-miR-6751-5p	0	0	0
hsa-miR-6751-3p	0	0	0
hsa-miR-6752-5p	0	0	0
hsa-miR-6752-3p	0	0	0
hsa-miR-6753-5p	0	0	0
hsa-miR-6753-3p	0	0	0
hsa-miR-6754-5p	0	0	0
hsa-miR-6754-3p	0	0	0
hsa-miR-6755-5p	0	0	0

hsa-miR-6755-3p	0	0	0
hsa-miR-6756-5p	0	0	0
hsa-miR-6756-3p	0	0	0
hsa-miR-6757-5p	0	0	0
hsa-miR-6757-3p	0	0	0
hsa-miR-6758-5p	0	0	0
hsa-miR-6758-3p	0	0	0
hsa-miR-6759-5p	0	0	0
hsa-miR-6759-3p	0	0	0
hsa-miR-6760-5p	0	0	0
hsa-miR-6760-3p	0	0	0
hsa-miR-6761-5p	0	0	0
hsa-miR-6761-3p	0	0	0
hsa-miR-6762-5p	0	0	0
hsa-miR-6762-3p	0	0	0
hsa-miR-6763-5p	0	0	0
hsa-miR-6763-3p	0	0	0
hsa-miR-6764-5p	0	0	0
hsa-miR-6764-3p	0	0	0
hsa-miR-6765-5p	0	0	0
hsa-miR-6765-3p	0	0	0
hsa-miR-6766-5p	0	0	0
hsa-miR-6766-3p	0	0	0
hsa-miR-6767-5p	0	0	0
hsa-miR-6767-3p	0	0	0
hsa-miR-6768-5p	0	0	0
hsa-miR-6768-3p	0	0	0
hsa-miR-6769a-5p	0	0	0
hsa-miR-6769a-3p	0	0	0
hsa-miR-6770-5p	0	0	0
hsa-miR-6770-3p	0	0	0
hsa-miR-6771-5p	0	0	0
hsa-miR-6771-3p	0	0	0
hsa-miR-6772-5p	0	0	0
hsa-miR-6772-3p	0	0	0
hsa-miR-6773-5p	0	0	0
hsa-miR-6773-3p	0	0	0
hsa-miR-6774-5p	0	0	0
hsa-miR-6774-3p	0	0	0
hsa-miR-6775-5p	0	0	0
hsa-miR-6775-3p	0	0	0
hsa-miR-6776-5p	0	0	0
hsa-miR-6776-3p	0	0	0
hsa-miR-6777-5p	0	0	0
hsa-miR-6777-3p	0	0	0
hsa-miR-6778-5p	0	0	0
hsa-miR-6778-3p	0	0	0
hsa-miR-6779-5p	1	0	0
hsa-miR-6779-3p	0	0	0
hsa-miR-6780a-5p	0	0	0
hsa-miR-6780a-3p	0	0	0
hsa-miR-6781-5p	0	0	0

hsa-miR-6781-3p	0	0	0
hsa-miR-6782-5p	0	0	0
hsa-miR-6782-3p	0	0	0
hsa-miR-6783-5p	0	0	0
hsa-miR-6783-3p	0	0	0
hsa-miR-6784-5p	0	0	0
hsa-miR-6784-3p	0	0	0
hsa-miR-6785-5p	0	0	0
hsa-miR-6785-3p	0	0	0
hsa-miR-6786-5p	0	0	0
hsa-miR-6786-3p	0	0	0
hsa-miR-6787-5p	0	0	0
hsa-miR-6787-3p	0	0	0
hsa-miR-6788-5p	0	0	0
hsa-miR-6788-3p	0	0	0
hsa-miR-6789-5p	0	0	0
hsa-miR-6789-3p	0	0	0
hsa-miR-6790-5p	0	0	0
hsa-miR-6790-3p	0	0	0
hsa-miR-6791-5p	0	0	0
hsa-miR-6791-3p	0	0	0
hsa-miR-6792-5p	0	0	0
hsa-miR-6792-3p	0	0	0
hsa-miR-6793-5p	0	0	0
hsa-miR-6793-3p	0	0	0
hsa-miR-6794-5p	0	0	0
hsa-miR-6794-3p	0	0	0
hsa-miR-6795-5p	0	0	0
hsa-miR-6795-3p	0	0	0
hsa-miR-6796-5p	0	0	0
hsa-miR-6796-3p	0	0	0
hsa-miR-6797-5p	0	0	0
hsa-miR-6797-3p	0	0	0
hsa-miR-6798-5p	0	0	0
hsa-miR-6798-3p	0	0	0
hsa-miR-6799-5p	0	0	0
hsa-miR-6799-3p	0	0	0
hsa-miR-6800-5p	0	0	0
hsa-miR-6800-3p	0	0	0
hsa-miR-6801-5p	0	0	0
hsa-miR-6801-3p	0	0	0
hsa-miR-6802-5p	0	0	0
hsa-miR-6802-3p	0	0	0
hsa-miR-6803-5p	0	0	0
hsa-miR-6803-3p	0	0	0
hsa-miR-6804-5p	0	0	0
hsa-miR-6804-3p	0	0	0
hsa-miR-6805-5p	0	0	0
hsa-miR-6805-3p	0	0	0
hsa-miR-6806-5p	0	0	0
hsa-miR-6806-3p	0	0	0
hsa-miR-6807-5p	0	0	0

hsa-miR-6807-3p	0	0	0
hsa-miR-6808-5p	0	0	0
hsa-miR-6808-3p	0	0	0
hsa-miR-6809-5p	0	0	0
hsa-miR-6809-3p	0	0	0
hsa-miR-6810-5p	0	0	0
hsa-miR-6810-3p	0	0	0
hsa-miR-6811-5p	0	0	0
hsa-miR-6811-3p	0	0	0
hsa-miR-6812-5p	0	0	0
hsa-miR-6812-3p	0	0	0
hsa-miR-6813-5p	0	0	0
hsa-miR-6813-3p	0	0	0
hsa-miR-6814-5p	0	0	0
hsa-miR-6814-3p	0	0	0
hsa-miR-6815-5p	0	0	0
hsa-miR-6815-3p	0	0	0
hsa-miR-6816-5p	0	0	0
hsa-miR-6816-3p	0	0	0
hsa-miR-6817-5p	0	0	0
hsa-miR-6817-3p	0	0	0
hsa-miR-6818-5p	0	0	0
hsa-miR-6818-3p	0	0	0
hsa-miR-6819-5p	0	0	0
hsa-miR-6819-3p	0	0	0
hsa-miR-6820-5p	0	0	0
hsa-miR-6820-3p	0	0	0
hsa-miR-6821-5p	0	0	0
hsa-miR-6821-3p	0	0	0
hsa-miR-6822-5p	0	0	0
hsa-miR-6822-3p	0	0	0
hsa-miR-6823-5p	0	0	0
hsa-miR-6823-3p	0	0	0
hsa-miR-6824-5p	0	0	0
hsa-miR-6824-3p	0	0	0
hsa-miR-6825-5p	0	0	0
hsa-miR-6825-3p	0	0	0
hsa-miR-6826-5p	0	0	0
hsa-miR-6826-3p	0	0	0
hsa-miR-6827-5p	0	0	0
hsa-miR-6827-3p	0	0	0
hsa-miR-6828-5p	0	0	0
hsa-miR-6828-3p	0	0	0
hsa-miR-6829-5p	0	0	0
hsa-miR-6829-3p	0	0	0
hsa-miR-6830-5p	0	0	0
hsa-miR-6830-3p	0	0	0
hsa-miR-6831-5p	0	0	0
hsa-miR-6831-3p	0	0	0
hsa-miR-6832-5p	0	0	0
hsa-miR-6832-3p	0	0	0
hsa-miR-6833-5p	0	0	0

hsa-miR-6833-3p	0	0	0
hsa-miR-6834-5p	0	0	0
hsa-miR-6834-3p	0	0	0
hsa-miR-6835-5p	0	0	0
hsa-miR-6835-3p	0	0	0
hsa-miR-6780b-5p	0	0	0
hsa-miR-6780b-3p	0	0	0
hsa-miR-6836-5p	0	0	0
hsa-miR-6836-3p	0	0	0
hsa-miR-6837-5p	0	0	0
hsa-miR-6837-3p	0	0	0
hsa-miR-6838-5p	0	0	0
hsa-miR-6838-3p	0	0	0
hsa-miR-6839-5p	0	0	0
hsa-miR-6839-3p	0	0	0
hsa-miR-6840-5p	0	0	0
hsa-miR-6840-3p	0	0	0
hsa-miR-6841-5p	0	0	0
hsa-miR-6841-3p	0	0	0
hsa-miR-6842-5p	0	0	0
hsa-miR-6842-3p	0	0	0
hsa-miR-6843-3p	0	0	0
hsa-miR-6844	0	0	0
hsa-miR-6845-5p	0	0	0
hsa-miR-6845-3p	0	0	0
hsa-miR-6846-5p	0	0	0
hsa-miR-6846-3p	0	0	0
hsa-miR-6847-5p	0	0	0
hsa-miR-6847-3p	0	0	0
hsa-miR-6848-5p	0	0	0
hsa-miR-6848-3p	0	0	0
hsa-miR-6849-5p	0	0	0
hsa-miR-6849-3p	0	0	0
hsa-miR-6850-5p	0	0	0
hsa-miR-6850-3p	0	0	0
hsa-miR-6851-5p	0	0	0
hsa-miR-6851-3p	0	0	0
hsa-miR-6852-5p	0	0	0
hsa-miR-6852-3p	0	0	0
hsa-miR-6853-5p	0	0	0
hsa-miR-6853-3p	0	0	0
hsa-miR-6854-5p	0	0	0
hsa-miR-6854-3p	0	0	0
hsa-miR-6855-5p	0	0	0
hsa-miR-6855-3p	0	0	0
hsa-miR-6856-5p	0	0	0
hsa-miR-6856-3p	0	0	0
hsa-miR-6857-5p	0	0	0
hsa-miR-6857-3p	0	0	0
hsa-miR-6858-5p	0	0	0
hsa-miR-6858-3p	0	0	0
hsa-miR-6859-5p	0	0	0

hsa-miR-6859-3p	0	0	0
hsa-miR-6769b-5p	0	0	0
hsa-miR-6769b-3p	0	0	0
hsa-miR-6860	0	0	0
hsa-miR-6861-5p	0	0	0
hsa-miR-6861-3p	0	0	0
hsa-miR-6862-5p	0	0	0
hsa-miR-6862-3p	0	0	0
hsa-miR-6863	0	0	0
hsa-miR-6864-5p	0	0	0
hsa-miR-6864-3p	0	0	0
hsa-miR-6865-5p	0	0	0
hsa-miR-6865-3p	0	0	0
hsa-miR-6866-5p	0	0	0
hsa-miR-6866-3p	0	0	0
hsa-miR-6867-5p	0	0	0
hsa-miR-6867-3p	0	0	0
hsa-miR-6868-5p	0	0	0
hsa-miR-6868-3p	0	0	0
hsa-miR-6869-5p	0	0	0
hsa-miR-6869-3p	0	0	0
hsa-miR-6870-5p	0	0	0
hsa-miR-6870-3p	0	0	0
hsa-miR-6871-5p	0	0	0
hsa-miR-6871-3p	0	0	0
hsa-miR-6872-5p	0	0	0
hsa-miR-6872-3p	0	0	0
hsa-miR-6873-5p	0	0	0
hsa-miR-6873-3p	0	0	0
hsa-miR-6874-5p	0	0	0
hsa-miR-6874-3p	0	0	0
hsa-miR-6875-5p	0	0	0
hsa-miR-6875-3p	0	0	0
hsa-miR-6876-5p	0	0	0
hsa-miR-6876-3p	0	0	0
hsa-miR-6877-5p	0	0	0
hsa-miR-6877-3p	0	0	0
hsa-miR-6878-5p	0	0	0
hsa-miR-6878-3p	0	0	0
hsa-miR-6879-5p	0	0	0
hsa-miR-6879-3p	0	0	0
hsa-miR-6880-5p	0	0	0
hsa-miR-6880-3p	0	0	0
hsa-miR-6881-5p	0	0	0
hsa-miR-6881-3p	0	0	0
hsa-miR-6882-5p	0	0	0
hsa-miR-6882-3p	0	0	0
hsa-miR-6883-5p	0	0	0
hsa-miR-6883-3p	0	0	0
hsa-miR-6884-5p	0	0	0
hsa-miR-6884-3p	0	0	0
hsa-miR-6885-5p	0	0	0

hsa-miR-6885-3p	0	0	0
hsa-miR-6886-5p	0	0	0
hsa-miR-6886-3p	0	0	0
hsa-miR-6887-5p	0	0	0
hsa-miR-6887-3p	0	0	0
hsa-miR-6888-5p	0	0	0
hsa-miR-6888-3p	0	0	0
hsa-miR-6889-5p	0	0	0
hsa-miR-6889-3p	0	0	0
hsa-miR-6890-5p	0	0	0
hsa-miR-6890-3p	0	0	0
hsa-miR-6891-5p	0	0	0
hsa-miR-6891-3p	0	0	0
hsa-miR-6892-5p	0	0	0
hsa-miR-6892-3p	0	0	1
hsa-miR-6893-5p	0	0	0
hsa-miR-6893-3p	0	0	0
hsa-miR-6894-5p	0	0	0
hsa-miR-6894-3p	0	0	0
hsa-miR-6895-5p	0	0	0
hsa-miR-6895-3p	0	0	0
hsa-miR-7106-5p	0	0	0
hsa-miR-7106-3p	0	0	0
hsa-miR-7107-5p	0	0	0
hsa-miR-7107-3p	0	0	0
hsa-miR-7108-5p	0	0	0
hsa-miR-7108-3p	0	0	0
hsa-miR-7109-5p	0	0	0
hsa-miR-7109-3p	0	0	0
hsa-miR-7110-5p	0	0	0
hsa-miR-7110-3p	0	0	0
hsa-miR-7111-5p	0	0	0
hsa-miR-7111-3p	0	0	0
hsa-miR-7112-5p	0	0	0
hsa-miR-7112-3p	0	0	0
hsa-miR-7113-5p	0	0	0
hsa-miR-7113-3p	0	0	0
hsa-miR-7114-5p	0	0	0
hsa-miR-7114-3p	0	0	0
hsa-miR-7150	0	0	0
hsa-miR-7151-5p	0	0	0
hsa-miR-7151-3p	0	0	0
hsa-miR-7152-5p	0	0	0
hsa-miR-7152-3p	0	0	0
hsa-miR-7153-5p	0	0	0
hsa-miR-7153-3p	0	0	0
hsa-miR-7154-5p	0	0	0
hsa-miR-7154-3p	0	0	0
hsa-miR-7155-5p	0	0	0
hsa-miR-7155-3p	0	0	0
hsa-miR-7156-5p	0	0	0
hsa-miR-7156-3p	0	0	0

hsa-miR-7157-5p	0	0	0
hsa-miR-7157-3p	0	0	0
hsa-miR-7158-5p	0	0	0
hsa-miR-7158-3p	0	0	0
hsa-miR-7161-5p	0	0	0
hsa-miR-7161-3p	0	0	0
hsa-miR-7159-5p	0	0	0
hsa-miR-7159-3p	0	0	0
hsa-miR-7160-5p	0	0	0
hsa-miR-7160-3p	0	0	0
hsa-miR-7162-5p	0	0	0
hsa-miR-7162-3p	0	0	0
hsa-miR-7515	0	0	0
hsa-miR-7641	0	0	0
hsa-miR-7702	0	0	0
hsa-miR-7703	0	0	0
hsa-miR-7704	0	0	0
hsa-miR-7705	0	0	0
hsa-miR-7706	0	0	0
hsa-miR-7843-5p	0	0	0
hsa-miR-7843-3p	0	0	0
hsa-miR-4433b-5p	0	0	0
hsa-miR-4433b-3p	0	0	0
hsa-miR-1273h-5p	0	0	0
hsa-miR-1273h-3p	0	0	0
hsa-miR-6516-5p	0	0	0
hsa-miR-6516-3p	0	0	0
hsa-miR-7844-5p	0	0	0
hsa-miR-7845-5p	0	0	0
hsa-miR-7846-3p	0	0	0
hsa-miR-7847-3p	0	0	0
hsa-miR-7848-3p	0	0	0
hsa-miR-7849-3p	0	0	0
hsa-miR-7850-5p	0	0	0
hsa-miR-7851-3p	0	0	0
hsa-miR-7852-3p	0	0	0
hsa-miR-7853-5p	0	0	0
hsa-miR-7854-3p	0	0	0
hsa-miR-7855-5p	0	0	0
hsa-miR-7856-5p	0	0	0
hsa-miR-548ba	0	0	0
hsa-miR-7973	0	0	0
hsa-miR-7974	0	0	0
hsa-miR-7975	0	0	0
hsa-miR-7976	0	0	0
hsa-miR-7977	0	0	0
hsa-miR-7978	0	0	0
hsa-miR-8052	0	0	0
hsa-miR-8053	0	0	0
hsa-miR-8054	0	0	0
hsa-miR-8055	0	0	0
hsa-miR-8056	0	0	0

hsa-miR-8057	0	0	0
hsa-miR-8058	0	0	0
hsa-miR-8059	0	0	0
hsa-miR-8060	0	0	0
hsa-miR-8061	0	0	0
hsa-miR-8062	0	0	0
hsa-miR-8063	0	0	0
hsa-miR-8064	0	0	0
hsa-miR-8065	0	0	0
hsa-miR-8066	0	0	0
hsa-miR-8067	0	0	0
hsa-miR-8068	0	0	0
hsa-miR-8069	0	0	0
hsa-miR-8070	0	0	0
hsa-miR-8071	0	0	0
hsa-miR-8072	0	0	0
hsa-miR-8073	0	0	0
hsa-miR-8074	0	0	0
hsa-miR-8075	0	0	0
hsa-miR-8076	0	0	0
hsa-miR-8077	0	0	0
hsa-miR-8078	0	0	0
hsa-miR-8079	0	0	0
hsa-miR-8080	0	0	0
hsa-miR-8081	0	0	0
hsa-miR-8082	0	0	0
hsa-miR-8083	0	0	0
hsa-miR-8084	0	0	0
hsa-miR-8085	0	0	0
hsa-miR-8086	0	0	0
hsa-miR-8087	0	0	0
hsa-miR-8088	0	0	0
hsa-miR-8089	0	0	0
14q0	0	0	0
14qI-1	0	0	0
14qI-1	0	0	0
14qI-2	0	0	0
14qI-3	0	0	0
14qI-4	0	0	0
14qI-4	0	0	0
14qI-5	0	0	0
14qI-6	0	0	0
14qI-7	0	0	0
14qI-8	0	0	0
14qI-8	0	0	0
14qI-9	0	0	0
14qII-10	0	0	0
14qII-11	0	0	0
14qII-12	0	0	0
14qII-12	0	0	0
14qII-13	0	0	0
14qII-14	0	0	0

14qII-14	0	0	0
14qII-15	0	0	0
14qII-16	0	0	0
14qII-17	0	0	0
14qII-18	0	0	0
14qII-18	0	0	0
14qII-19	0	0	0
14qII-1	0	0	0
14qII-1	0	0	0
14qII-20	0	0	0
14qII-20	0	0	0
14qII-21	0	0	0
14qII-22	0	0	0
14qII-23	0	0	0
14qII-24	0	0	0
14qII-25	0	0	0
14qII-26	0	0	0
14qII-26	0	0	0
14qII-27	0	0	0
14qII-27	0	0	0
14qII-28	0	0	0
14qII-29	0	0	0
14qII-29	0	0	0
14qII-2	0	0	0
14qII-30	0	0	0
14qII-31	0	0	0
14qII-3	0	0	0
14qII-3	0	0	0
14qII-4	0	0	0
14qII-5	0	0	0
14qII-6	0	0	0
14qII-7	0	0	0
14qII-8	0	0	0
14qII-8	0	0	0
14qII-9	0	0	0
ACA10	0	0	0
ACA11	0	0	0
ACA12	0	0	0
ACA12	0	0	0
ACA13	0	0	0
ACA14a	0	0	0
ACA14b	0	0	0
ACA15	0	0	0
ACA16	0	0	0
ACA16	0	0	0
ACA17	0	0	0
ACA18	0	0	0
ACA19	0	0	0
ACA1	0	0	0
ACA20	0	0	0
ACA20	0	0	0
ACA21	0	0	0

ACA22	0	0	0
ACA23	0	0	0
ACA24	0	0	0
ACA24	0	0	0
ACA25	0	0	0
ACA26	0	0	0
ACA27	0	0	0
ACA28	0	0	0
ACA29	0	0	0
ACA2a	0	0	0
ACA2b	0	0	0
ACA2b	0	0	0
ACA2b	0	0	0
ACA3-2	0	0	0
ACA3-2	0	0	0
ACA30	0	0	0
ACA31	0	0	0
ACA32	0	0	0
ACA32	0	0	0
ACA33	0	0	0
ACA33	0	0	0
ACA34	0	0	0
ACA34	0	0	0
ACA34	0	0	0
ACA35	0	0	0
ACA36B	0	0	0
ACA36B	0	0	0
ACA36B	0	0	0
ACA36	0	0	0
ACA37	0	0	0
ACA37	0	0	0
ACA38	0	0	0
ACA39	0	0	0
ACA39	0	0	0
ACA3	0	0	0
ACA40	0	0	0
ACA41	0	0	0
ACA41	0	0	0
ACA42	0	0	0
ACA43	0	0	0
ACA43	0	0	0
ACA44	0	0	0
ACA44	0	0	0
ACA44	0	0	0
ACA45	0	0	0
ACA45	0	0	0
ACA46	0	0	0
ACA47	0	0	0
ACA47	0	0	0
ACA48	0	0	0
ACA48	0	0	0
ACA49	0	0	0

ACA4	0	0	0
ACA50	0	0	0
ACA51	0	0	0
ACA52	0	0	0
ACA53	0	0	0
ACA54	0	0	0
ACA55	0	0	0
ACA56	0	0	0
ACA57	0	0	0
ACA57	0	0	0
ACA58	0	0	0
ACA59B	0	0	0
ACA59	0	0	0
ACA5	0	0	0
ACA5	0	0	0
ACA5b	0	0	0
ACA5b	0	0	0
ACA5c	0	0	0
ACA60	0	0	0
ACA61	0	0	0
ACA62	0	0	0
ACA63	0	0	0
ACA63	0	0	0
ACA64	0	0	0
ACA65	0	0	0
ACA66	0	0	0
ACA67B	0	0	0
ACA67B	0	0	0
ACA67	0	0	0
ACA67	0	0	0
ACA68	0	0	0
ACA68	0	0	0
ACA6	0	0	0
ACA7B	0	0	0
ACA7	0	0	0
ACA8	0	0	0
ACA9	0	0	0
ENSG00000199196	0	0	0
ENSG00000199212	0	0	0
ENSG00000199231	0	0	0
ENSG00000199231	0	0	0
ENSG00000199231	0	0	0
ENSG00000199262	0	0	0
ENSG00000199282	0	0	0
ENSG00000199321	0	0	0
ENSG00000199321	0	0	0
ENSG00000199363	0	0	0
ENSG00000199370	0	0	0
ENSG00000199392	0	0	0
ENSG00000199405	0	0	0
ENSG00000199405	0	0	0
ENSG199411	0	0	0

ENSG299411	0	0	0
ENSG00000199452	0	0	0
ENSG00000199470	0	0	0
ENSG00000199473	0	0	0
ENSG00000199474	0	0	0
ENSG00000199474	0	0	0
ENSG00000199566	0	0	0
ENSG00000199571	0	0	0
ENSG00000199633	0	0	0
ENSG00000199666	0	0	0
ENSG00000199666	0	0	0
ENSG00000199675	0	0	0
ENSG00000199713	0	0	0
ENSG00000199727	0	0	0
ENSG00000199769	0	0	0
ENSG00000199783	0	0	0
ENSG00000199787	0	0	0
ENSG00000199815	0	0	0
ENSG00000199851	0	0	0
ENSG00000199851	0	0	0
ENSG00000199856	0	0	0
ENSG00000199856	0	0	0
ENSG00000199857	0	0	0
ENSG00000199894	0	0	0
ENSG00000199894	0	0	0
ENSG00000199894	0	0	0
ENSG00000199927	0	0	0
ENSG00000199934	0	0	0
ENSG00000199959	0	0	0
ENSG00000199959	0	0	0
ENSG00000199959	0	0	0
ENSG00000199977	0	0	0
ENSG00000200026	0	0	0
ENSG00000200026	0	0	0
ENSG00000200042	0	0	0
ENSG00000200051	0	0	0
ENSG00000200063	0	0	0
ENSG00000200063	0	0	0
ENSG00000200072	0	0	0
ENSG00000200075	0	0	0
ENSG00000200112	0	0	0
ENSG00000200113	0	0	0
ENSG00000200130	0	0	0
ENSG00000200150	0	0	0
ENSG00000200191	0	0	0
ENSG00000200206	0	0	0
ENSG00000200222	0	0	0
ENSG00000200235	0	0	0
ENSG00000200235	0	0	0
ENSG00000200237	0	0	0
ENSG00000200288	0	0	0
ENSG200288	0	0	0

ENSG200294	0	0	0
ENSG00000200294	0	0	0
ENSG00000200318	0	0	0
ENSG00000200355	0	0	0
ENSG00000200377	0	0	0
ENSG00000200385	0	0	0
ENSG00000200398	0	0	0
ENSG00000200418	0	0	0
ENSG00000200422	0	0	0
ENSG00000200442	0	0	0
ENSG00000200442	0	0	0
ENSG00000200492	0	0	0
ENSG00000200496	0	0	0
ENSG00000200536	0	0	0
ENSG00000200538	0	0	0
ENSG00000200538	0	0	0
ENSG00000200545	0	0	0
ENSG00000200620	0	0	0
ENSG00000200652	0	0	0
ENSG00000200652	0	0	0
ENSG00000200677	0	0	0
ENSG00000200677	0	0	0
ENSG00000200693	0	0	0
ENSG00000200706	0	0	0
ENSG00000200706	0	0	0
ENSG00000200733	0	0	0
ENSG00000200753	0	0	0
ENSG00000200801	0	0	0
ENSG00000200879	0	0	0
ENSG00000200891	0	0	0
ENSG00000200897	0	0	0
ENSG00000200897	0	0	0
ENSG00000200969	0	0	0
ENSG00000200969	0	0	0
ENSG00000200991	0	0	0
ENSG00000200991	0	0	0
ENSG00000200999	0	0	0
ENSG00000200999	0	0	0
ENSG00000201003	0	0	0
ENSG00000201009	0	0	0
ENSG00000201009	0	0	0
ENSG00000201009	0	0	0
ENSG00000201025	0	0	0
ENSG00000201036	0	0	0
ENSG00000201042	0	0	0
ENSG00000201042	0	0	0
ENSG00000201042	0	0	0
ENSG00000201129	0	0	0
ENSG00000201133	0	0	0
ENSG00000201151	0	0	0
ENSG00000201157	0	0	0
ENSG00000201199	0	0	0

ENSG00000201209	0	0	0
ENSG00000201229	0	0	0
ENSG00000201245	0	0	0
ENSG00000201245	0	0	0
ENSG00000201300	0	0	0
ENSG00000201316	0	0	0
ENSG00000201329	0	0	0
ENSG00000201346	0	0	0
ENSG00000201348	0	0	0
ENSG00000201368	0	0	0
ENSG00000201376	0	0	0
ENSG00000201384	0	0	0
ENSG00000201388	0	0	0
ENSG00000201393	0	0	0
ENSG00000201398	0	0	0
ENSG00000201398	0	0	0
ENSG00000201407	0	0	0
ENSG00000201410	0	0	0
ENSG00000201448	0	0	0
ENSG00000201448	0	0	0
ENSG00000201465	0	0	0
ENSG00000201467	0	0	0
ENSG00000201467	0	0	0
ENSG00000201500	0	0	0
ENSG00000201502	0	0	0
ENSG00000201502	0	0	0
ENSG00000201516	0	0	0
ENSG00000201541	0	0	0
ENSG00000201541	0	0	0
ENSG00000201542	0	0	0
ENSG00000201592	0	0	0
ENSG00000201619	0	0	0
ENSG00000201619	0	0	0
ENSG00000201660	0	0	0
ENSG00000201666	0	0	0
ENSG00000201674	0	0	0
ENSG00000201701	0	0	0
ENSG00000201701	0	0	0
ENSG00000201710	0	0	0
ENSG00000201710	0	0	0
ENSG00000201733	0	0	0
ENSG00000201733	0	0	0
ENSG00000201791	0	0	0
ENSG00000201791	0	0	0
ENSG00000201807	0	0	0
ENSG00000201807	0	0	0
ENSG00000201809	0	0	0
ENSG00000201810	0	0	0
ENSG00000201810	0	0	0
ENSG00000201811	0	0	0
ENSG00000201816	0	0	0
ENSG00000201827	0	0	0

ENSG00000201827	0	0	0
ENSG00000201847	0	0	0
ENSG00000201847	0	0	0
ENSG00000201853	0	0	0
ENSG00000201863	0	0	0
ENSG00000201863	0	0	0
ENSG00000201882	0	0	0
ENSG00000201898	0	0	0
ENSG00000201944	0	0	0
ENSG00000201945	0	0	0
ENSG00000201957	0	0	0
ENSG00000201980	0	0	0
ENSG00000202023	0	0	0
ENSG00000202023	0	0	0
ENSG00000202059	0	0	0
ENSG00000202059	0	0	0
ENSG00000202183	0	0	0
ENSG00000202189	0	0	0
ENSG00000202216	0	0	0
ENSG00000202216	0	0	0
ENSG00000202231	0	0	0
ENSG00000202233	0	0	0
ENSG00000202252	0	0	0
ENSG00000202268	0	0	0
ENSG00000202268	0	0	0
ENSG00000202269	0	0	0
ENSG00000202269	0	0	0
ENSG00000202275	0	0	0
ENSG00000202283	0	0	0
ENSG00000202335	0	0	0
ENSG00000202335	0	0	0
ENSG00000202343	0	0	0
ENSG00000202374	0	0	0
ENSG00000202377	0	0	0
ENSG00000202379	0	0	0
ENSG00000202389	0	0	0
ENSG00000202434	0	0	0
ENSG00000202440	0	0	0
ENSG00000202449	0	0	0
ENSG00000202479	0	0	0
ENSG00000202479	0	0	0
ENSG00000202482	0	0	0
ENSG00000202498	0	0	0
ENSG00000202517	0	0	0
ENSG00000202537	0	0	0
ENSG00000206592	0	0	0
ENSG00000206603	0	0	0
ENSG00000206603	0	0	0
ENSG00000206637	0	0	0
ENSG00000206647	0	0	0
ENSG00000206649	0	0	0
ENSG206649	0	0	0

ENSG207661	0	0	0
ENSG00000206731	0	0	0
ENSG00000206761	0	0	0
ENSG00000206761	0	0	0
ENSG00000206776	0	0	0
ENSG00000206780	0	0	0
ENSG00000206780	0	0	0
ENSG00000206785	0	0	0
ENSG00000206849	0	0	0
ENSG00000206849	0	0	0
ENSG00000206853	0	0	0
ENSG00000206878	0	0	0
ENSG00000206886	0	0	0
ENSG00000206886	0	0	0
ENSG00000206897	0	0	0
ENSG00000206897	0	0	0
ENSG00000206898	0	0	0
ENSG00000206901	0	0	0
ENSG00000206903	0	0	0
ENSG00000206903	0	0	0
ENSG00000206903	0	0	0
ENSG00000206909	0	0	0
ENSG00000206913	0	0	0
ENSG00000206913	0	0	0
ENSG00000206947	0	0	0
ENSG00000206947	0	0	0
ENSG00000206958	0	0	0
ENSG00000206958	0	0	0
ENSG00000206961	0	0	0
ENSG00000206976	0	0	0
ENSG00000206977	0	0	0
ENSG00000206987	0	0	0
ENSG00000207002	0	0	0
ENSG00000207002	0	0	0
ENSG00000207022	0	0	0
ENSG00000207027	0	0	0
ENSG00000207062	0	0	0
ENSG00000207084	0	0	0
ENSG00000207084	0	0	0
ENSG00000207094	0	0	0
ENSG00000207098	0	0	0
ENSG00000207098	0	0	0
ENSG00000207100	0	0	0
ENSG00000207100	0	0	0
ENSG00000207109	0	0	0
ENSG00000207118	0	0	0
ENSG00000207119	0	0	0
ENSG00000207130	0	0	0
ENSG00000207130	0	0	0
ENSG00000207147	0	0	0
ENSG00000207171	0	0	0
ENSG207171	0	0	0

ENSG207177	0	0	0
ENSG00000207187	0	0	0
ENSG00000207199	0	0	0
ENSG00000207199	0	0	0
ENSG00000207215	0	0	0
ENSG00000207217	0	0	0
ENSG00000207244	0	0	0
ENSG00000207249	0	0	0
ENSG00000207274	0	0	0
ENSG00000207299	0	0	0
ENSG00000207344	0	0	0
ENSG00000207407	0	0	0
ENSG00000207410	0	0	0
ENSG00000207419	0	0	0
ENSG00000207430	0	0	0
ENSG00000207432	0	0	0
ENSG00000207444	0	0	0
ENSG00000207444	0	0	0
ENSG00000207502	0	0	0
ENSG00000207502	0	0	0
ENSG00000207503	0	0	0
ENSG00000207516	0	0	0
ENSG00000208308	0	0	0
ENSG00000212134	0	0	0
ENSG00000212144	0	0	0
ENSG00000212145	0	0	0
ENSG00000212148	0	0	0
ENSG00000212148	0	0	0
ENSG00000212149	0	0	0
ENSG00000212161	0	0	0
ENSG00000212161	0	0	0
ENSG00000212165	0	0	0
ENSG00000212165	0	0	0
ENSG00000212168	0	0	0
ENSG00000212175	0	0	0
ENSG00000212181	0	0	0
ENSG00000212181	0	0	0
ENSG00000212182	0	0	0
ENSG00000212187	0	0	0
ENSG00000212191	0	0	0
ENSG00000212195	0	0	0
ENSG00000212206	0	0	0
ENSG00000212206	0	0	0
ENSG00000212211	0	0	0
ENSG00000212214	0	0	0
ENSG00000212214	0	0	0
ENSG00000212224	0	0	0
ENSG00000212224	0	0	0
ENSG00000212228	0	0	0
ENSG00000212229	0	0	0
ENSG00000212249	0	0	0
ENSG00000212264	0	0	0

ENSG00000212266	0	0	0
ENSG00000212270	0	0	0
ENSG00000212273	0	0	0
ENSG00000212277	0	0	0
ENSG00000212278	0	0	0
ENSG00000212279	0	0	0
ENSG00000212284	0	0	0
ENSG00000212293	0	0	0
ENSG00000212295	0	0	0
ENSG00000212302	0	0	0
ENSG00000212309	0	0	0
ENSG00000212309	0	0	0
ENSG00000212321	0	0	0
ENSG00000212338	0	0	0
ENSG00000212338	0	0	0
ENSG00000212342	0	0	0
ENSG00000212342	0	0	0
ENSG00000212347	0	0	0
ENSG00000212363	0	0	0
ENSG00000212363	0	0	0
ENSG00000212367	0	0	0
ENSG00000212371	0	0	0
ENSG00000212377	0	1	0
ENSG00000212378	0	0	0
ENSG00000212378	0	0	0
ENSG00000212378	0	0	0
ENSG00000212380	0	0	0
ENSG00000212383	0	0	0
ENSG00000212391	0	0	0
ENSG00000212395	0	0	0
ENSG00000212397	0	0	0
ENSG00000212411	0	0	0
ENSG00000212411	0	0	0
ENSG00000212414	0	0	0
ENSG00000212415	0	0	0
ENSG00000212421	0	0	0
ENSG00000212422	0	0	0
ENSG00000212428	0	0	0
ENSG00000212428	0	0	0
ENSG00000212432	0	0	0
ENSG00000212434	0	0	0
ENSG00000212440	0	0	0
ENSG00000212445	0	0	0
ENSG00000212455	0	0	0
ENSG00000212458	0	0	0
ENSG00000212461	0	0	0
ENSG00000212461	0	0	0
ENSG00000212479	0	0	0
ENSG00000212490	0	0	0
ENSG00000212502	0	0	0
ENSG00000212511	0	0	0
ENSG00000212517	0	0	0

ENSG00000212517	0	0	0
ENSG00000212528	0	0	0
ENSG00000212529	0	0	0
ENSG00000212532	0	0	0
ENSG00000212532	0	0	0
ENSG00000212533	0	0	0
ENSG00000212538	0	0	0
ENSG00000212539	0	0	0
ENSG00000212551	0	0	0
ENSG00000212553	0	0	0
ENSG00000212553	0	0	0
ENSG00000212558	0	0	0
ENSG00000212558	0	0	0
ENSG00000212565	0	0	0
ENSG00000212567	0	0	0
ENSG00000212579	0	0	0
ENSG00000212580	0	0	0
ENSG00000212581	0	0	0
ENSG00000212586	0	0	0
ENSG00000212587	0	0	0
ENSG00000212589	0	0	0
ENSG00000212589	0	0	0
ENSG00000212590	0	0	0
ENSG00000212593	0	0	0
ENSG00000212594	0	0	0
ENSG00000212598	0	0	0
ENSG00000212604	0	0	0
ENSG00000212604	0	0	0
ENSG00000212608	0	0	0
ENSG00000212610	0	0	0
ENSG00000212611	0	0	0
ENSG00000212615	0	0	0
ENSG00000212618	0	0	0
ENSG00000212620	0	0	0
ENSG00000212624	0	0	0
ENSG00000212624	0	0	0
ENSG00000212626	0	0	0
ENSG00000220986	0	0	0
ENSG00000221040	0	0	0
ENSG00000221043	0	0	0
ENSG00000221044	0	0	0
ENSG00000221060	0	0	0
ENSG00000221060	0	0	0
ENSG00000221083	0	0	0
ENSG00000221090	0	0	0
ENSG00000221093	0	0	0
ENSG00000221125	0	0	0
ENSG00000221139	0	0	0
ENSG00000221148	0	0	0
ENSG00000221164	0	0	0
ENSG00000221164	0	0	0
ENSG221245	0	0	0

ENSG231245	0	0	0
ENSG00000221252	0	0	0
ENSG00000221300	0	0	0
ENSG00000221300	0	0	0
ENSG00000221332	0	0	0
ENSG00000221345	0	0	0
ENSG00000221376	0	0	0
ENSG00000221398	0	0	0
ENSG00000221400	0	0	0
ENSG00000221455	0	0	0
ENSG00000221461	0	0	0
ENSG00000221496	0	0	0
ENSG00000221498	0	0	0
ENSG00000221611	0	0	0
ENSG00000221633	0	0	0
ENSG00000221638	0	0	0
ENSG00000221639	0	0	0
ENSG00000221639	0	0	0
ENSG00000221673	0	0	0
ENSG00000221711	0	0	0
ENSG00000221719	0	0	0
ENSG00000221719	0	0	0
ENSG00000221750	0	0	0
ENSG00000222095	0	0	0
ENSG00000222145	0	0	0
ENSG00000222185	0	0	0
ENSG00000222345	0	0	0
ENSG00000222489	0	0	0
ENSG00000222588	0	0	0
ENSG00000222588	0	0	0
ENSG00000222604	0	0	0
ENSG00000222666	0	0	0
ENSG00000222937	0	0	0
ENSG00000222966	0	0	0
ENSG00000223004	0	0	0
ENSG00000223027	0	0	0
ENSG00000223111	0	0	0
ENSG00000223182	0	0	0
ENSG00000223294	0	0	0
ENSG00000238294	0	0	0
ENSG00000238295	0	0	0
ENSG00000238296	0	0	0
ENSG00000238297	0	0	0
ENSG00000238298	0	0	0
ENSG00000238298	0	0	0
ENSG00000238301	0	0	0
ENSG00000238305	0	0	0
ENSG00000238306	0	0	0
ENSG00000238309	0	0	0
ENSG00000238311	0	0	0
ENSG00000238312	0	0	0
ENSG238313	0	0	0

ENSG238313	0	0	0
ENSG00000238314	0	0	0
ENSG00000238316	0	0	0
ENSG00000238316	0	0	0
ENSG00000238318	0	0	0
ENSG00000238319	0	0	0
ENSG00000238319	0	0	0
ENSG00000238321	0	0	0
ENSG00000238322	0	0	0
ENSG00000238325	0	0	0
ENSG00000238326	0	0	0
ENSG00000238327	0	0	0
ENSG00000238327	0	0	0
ENSG00000238328	0	0	0
ENSG00000238329	0	0	0
ENSG00000238330	0	0	0
ENSG00000238334	0	0	0
ENSG00000238334	0	0	0
ENSG00000238336	0	0	0
ENSG00000238337	0	0	0
ENSG00000238337	0	0	0
ENSG00000238339	0	0	0
ENSG00000238339	0	0	0
ENSG00000238341	0	0	0
ENSG00000238341	0	0	0
ENSG00000238342	0	0	0
ENSG00000238342	0	0	0
ENSG00000238343	0	0	0
ENSG00000238343	0	0	0
ENSG00000238345	0	0	0
ENSG00000238345	0	0	0
ENSG00000238348	0	0	0
ENSG00000238348	0	0	0
ENSG00000238349	0	0	0
ENSG00000238350	0	0	0
ENSG00000238351	0	0	0
ENSG00000238351	0	0	0
ENSG00000238352	0	0	0
ENSG00000238354	0	0	0
ENSG00000238355	0	0	0
ENSG00000238359	0	0	0
ENSG00000238361	0	0	0
ENSG00000238368	0	0	0
ENSG00000238368	0	0	0
ENSG00000238369	0	0	0
ENSG00000238371	0	0	0
ENSG00000238372	0	0	0
ENSG00000238375	0	0	0
ENSG00000238376	0	0	0
ENSG00000238377	0	0	0
ENSG00000238383	0	0	0
ENSG00000238384	0	0	0

ENSG00000238387	0	0	0
ENSG00000238388	0	0	0
ENSG00000238388	0	0	0
ENSG00000238389	0	0	0
ENSG00000238389	0	0	0
ENSG00000238390	0	0	0
ENSG00000238394	0	0	0
ENSG00000238395	0	0	0
ENSG00000238398	0	0	0
ENSG00000238400	0	0	0
ENSG00000238401	0	0	0
ENSG00000238402	0	0	0
ENSG00000238403	0	0	0
ENSG00000238407	0	0	0
ENSG00000238408	0	0	0
ENSG00000238409	0	0	0
ENSG00000238410	0	0	0
ENSG00000238414	0	0	0
ENSG00000238416	0	0	0
ENSG00000238418	0	0	0
ENSG00000238422	0	0	0
ENSG00000238425	0	0	0
ENSG00000238428	0	0	0
ENSG00000238430	0	0	0
ENSG00000238430	0	0	0
ENSG00000238433	0	0	0
ENSG00000238433	0	0	0
ENSG00000238436	0	0	0
ENSG00000238436	0	0	0
ENSG00000238437	0	0	0
ENSG00000238437	0	0	0
ENSG00000238438	0	0	0
ENSG00000238440	0	0	0
ENSG00000238440	0	0	0
ENSG00000238445	0	0	0
ENSG00000238450	0	0	0
ENSG00000238450	0	0	0
ENSG00000238451	0	0	0
ENSG00000238453	0	0	0
ENSG00000238455	0	0	0
ENSG00000238458	0	0	0
ENSG00000238459	0	0	0
ENSG00000238462	0	0	0
ENSG00000238462	0	0	0
ENSG00000238463	0	0	0
ENSG00000238464	0	0	0
ENSG00000238464	0	0	0
ENSG00000238465	0	0	0
ENSG00000238466	0	0	0
ENSG00000238472	0	0	0
ENSG00000238473	0	0	0
ENSG238474	0	0	0

ENSG238575	0	0	0
ENSG00000238475	0	0	0
ENSG00000238480	0	0	0
ENSG00000238481	0	0	0
ENSG00000238481	0	0	0
ENSG00000238483	0	0	0
ENSG00000238483	0	0	0
ENSG00000238484	0	0	0
ENSG00000238485	0	0	0
ENSG00000238486	0	0	0
ENSG00000238488	0	0	0
ENSG00000238491	0	0	0
ENSG00000238492	0	0	0
ENSG00000238494	0	0	0
ENSG00000238496	0	0	0
ENSG00000238501	0	0	0
ENSG00000238501	0	0	0
ENSG00000238502	0	0	0
ENSG00000238502	0	0	0
ENSG00000238503	0	0	0
ENSG00000238506	0	0	0
ENSG00000238506	0	0	0
ENSG00000238507	0	0	0
ENSG00000238511	0	0	0
ENSG00000238513	0	0	0
ENSG00000238514	0	0	0
ENSG00000238515	0	0	0
ENSG00000238519	0	0	0
ENSG00000238520	0	0	0
ENSG00000238521	0	0	0
ENSG00000238522	0	0	0
ENSG00000238525	0	0	0
ENSG00000238525	0	0	0
ENSG00000238526	0	0	0
ENSG00000238528	0	0	0
ENSG00000238528	0	0	0
ENSG00000238530	0	0	0
ENSG00000238530	0	0	0
ENSG00000238533	0	0	0
ENSG00000238535	0	0	0
ENSG00000238536	0	0	0
ENSG00000238536	0	0	0
ENSG00000238537	0	0	0
ENSG00000238538	0	0	0
ENSG00000238541	0	0	0
ENSG00000238543	0	0	0
ENSG00000238544	0	0	0
ENSG00000238545	0	0	0
ENSG00000238545	0	0	0
ENSG00000238546	0	0	0
ENSG00000238549	0	0	0
ENSG238552	0	0	0

ENSG238656	0	0	0
ENSG00000238557	0	0	0
ENSG00000238557	0	0	0
ENSG00000238559	0	0	0
ENSG00000238563	0	0	0
ENSG00000238563	0	0	0
ENSG00000238564	0	0	0
ENSG00000238564	0	0	0
ENSG00000238565	0	0	0
ENSG00000238565	0	0	0
ENSG00000238566	0	0	0
ENSG00000238566	0	0	0
ENSG00000238567	0	0	0
ENSG00000238567	0	0	0
ENSG00000238568	0	0	0
ENSG00000238568	0	0	0
ENSG00000238569	0	0	0
ENSG00000238569	0	0	0
ENSG00000238570	0	0	0
ENSG00000238571	0	0	0
ENSG00000238571	0	0	0
ENSG00000238572	0	0	0
ENSG00000238575	0	0	0
ENSG00000238575	0	0	0
ENSG00000238576	0	0	0
ENSG00000238577	0	0	0
ENSG00000238579	0	0	0
ENSG00000238581	0	0	0
ENSG00000238582	0	0	0
ENSG00000238582	0	0	0
ENSG00000238583	0	0	0
ENSG00000238587	0	0	0
ENSG00000238587	0	0	0
ENSG00000238588	0	0	0
ENSG00000238591	0	0	0
ENSG00000238592	0	0	0
ENSG00000238594	0	0	0
ENSG00000238595	0	0	0
ENSG00000238596	0	0	0
ENSG00000238598	0	0	0
ENSG00000238598	0	0	0
ENSG00000238605	0	0	0
ENSG00000238605	0	0	0
ENSG00000238608	0	0	0
ENSG00000238611	0	0	0
ENSG00000238611	0	0	0
ENSG00000238612	0	0	0
ENSG00000238612	0	0	0
ENSG00000238615	0	0	0
ENSG00000238618	0	0	0
ENSG00000238620	0	0	0
ENSG238620	0	0	0

ENSG238724	0	0	0
ENSG00000238625	0	0	0
ENSG00000238625	0	0	0
ENSG00000238626	0	0	0
ENSG00000238628	0	0	0
ENSG00000238629	0	0	0
ENSG00000238629	0	0	0
ENSG00000238630	0	0	0
ENSG00000238631	0	0	0
ENSG00000238639	0	0	0
ENSG00000238642	0	0	0
ENSG00000238645	0	0	0
ENSG00000238646	0	0	0
ENSG00000238651	0	0	0
ENSG00000238651	0	0	0
ENSG00000238652	0	0	0
ENSG00000238654	0	0	0
ENSG00000238656	0	0	0
ENSG00000238657	0	0	0
ENSG00000238661	0	0	0
ENSG00000238662	0	0	0
ENSG00000238663	0	0	0
ENSG00000238665	0	0	0
ENSG00000238666	0	0	0
ENSG00000238666	0	0	0
ENSG00000238670	0	0	0
ENSG00000238673	0	0	0
ENSG00000238674	0	0	0
ENSG00000238676	0	0	0
ENSG00000238676	0	0	0
ENSG00000238679	0	0	0
ENSG00000238683	0	0	0
ENSG00000238683	0	0	0
ENSG00000238684	0	0	0
ENSG00000238685	0	0	0
ENSG00000238685	0	0	0
ENSG00000238686	0	0	0
ENSG00000238686	0	0	0
ENSG00000238687	0	0	0
ENSG00000238688	0	0	0
ENSG00000238690	0	0	0
ENSG00000238691	0	0	0
ENSG00000238692	0	0	0
ENSG00000238692	0	0	0
ENSG00000238693	0	0	0
ENSG00000238694	0	0	0
ENSG00000238695	0	0	0
ENSG00000238695	0	0	0
ENSG00000238696	0	0	0
ENSG00000238696	0	0	0
ENSG00000238699	0	0	0
ENSG238701	0	0	0

ENSG238701	0	0	0
ENSG00000238702	0	0	0
ENSG00000238703	0	0	0
ENSG00000238707	0	0	0
ENSG00000238708	0	0	0
ENSG00000238712	0	0	0
ENSG00000238714	0	0	0
ENSG00000238715	0	0	0
ENSG00000238715	0	0	0
ENSG00000238717	0	0	0
ENSG00000238718	0	0	0
ENSG00000238722	0	0	0
ENSG00000238724	0	0	0
ENSG00000238729	0	0	0
ENSG00000238729	0	0	0
ENSG00000238732	0	0	0
ENSG00000238733	0	0	0
ENSG00000238734	0	0	0
ENSG00000238736	0	0	0
ENSG00000238736	0	0	0
ENSG00000238739	0	0	0
ENSG00000238739	0	0	0
ENSG00000238740	0	0	0
ENSG00000238744	0	0	0
ENSG00000238745	0	0	0
ENSG00000238746	0	0	0
ENSG00000238746	0	0	0
ENSG00000238747	0	0	0
ENSG00000238747	0	0	0
ENSG00000238748	0	0	0
ENSG00000238748	0	0	0
ENSG00000238752	0	0	0
ENSG00000238753	0	0	0
ENSG00000238754	0	0	0
ENSG00000238756	0	0	0
ENSG00000238756	0	0	0
ENSG00000238760	0	0	0
ENSG00000238761	0	0	0
ENSG00000238761	0	0	0
ENSG00000238763	0	0	0
ENSG00000238764	0	0	0
ENSG00000238767	0	0	0
ENSG00000238767	0	0	0
ENSG00000238768	0	0	0
ENSG00000238769	0	0	0
ENSG00000238769	0	0	0
ENSG00000238770	0	0	0
ENSG00000238771	0	0	0
ENSG00000238772	0	0	0
ENSG00000238775	0	0	0
ENSG00000238781	0	0	0
ENSG00000238790	0	0	0

ENSG00000238791	1	0	0
ENSG00000238791	0	0	0
ENSG00000238792	0	0	0
ENSG00000238792	0	0	0
ENSG00000238796	0	0	0
ENSG00000238796	0	0	0
ENSG00000238798	0	0	0
ENSG00000238799	0	0	0
ENSG00000238800	0	0	0
ENSG00000238801	0	0	0
ENSG00000238801	0	0	0
ENSG00000238802	0	0	0
ENSG00000238804	0	0	0
ENSG00000238805	0	0	0
ENSG00000238806	0	0	0
ENSG00000238807	0	0	0
ENSG00000238807	0	0	0
ENSG00000238809	0	0	0
ENSG00000238811	0	0	0
ENSG00000238811	0	0	0
ENSG00000238815	0	0	0
ENSG00000238816	0	0	0
ENSG00000238818	0	0	0
ENSG00000238819	0	0	0
ENSG00000238819	0	0	0
ENSG00000238821	0	0	0
ENSG00000238822	0	0	0
ENSG00000238824	0	0	0
ENSG00000238824	0	0	0
ENSG00000238832	0	0	0
ENSG00000238834	0	0	0
ENSG00000238834	0	0	0
ENSG00000238838	0	0	0
ENSG00000238838	0	0	0
ENSG00000238840	0	0	0
ENSG00000238841	0	0	0
ENSG00000238843	0	0	0
ENSG00000238843	0	0	0
ENSG00000238849	0	0	0
ENSG00000238851	0	0	0
ENSG00000238851	0	0	0
ENSG00000238852	0	0	0
ENSG00000238853	0	0	0
ENSG00000238854	0	0	0
ENSG00000238855	0	0	0
ENSG00000238856	0	0	0
ENSG00000238856	0	0	0
ENSG00000238857	0	0	0
ENSG00000238858	0	0	0
ENSG00000238858	0	0	0
ENSG00000238859	0	0	0
ENSG238860	0	0	0

ENSG238963	0	0	0
ENSG00000238864	0	0	0
ENSG00000238865	0	0	0
ENSG00000238868	0	0	0
ENSG00000238869	0	0	0
ENSG00000238869	0	0	0
ENSG00000238871	0	0	0
ENSG00000238872	0	0	0
ENSG00000238872	0	0	0
ENSG00000238874	0	0	0
ENSG00000238878	0	0	0
ENSG00000238885	0	0	0
ENSG00000238887	0	0	0
ENSG00000238888	0	0	0
ENSG00000238889	0	0	0
ENSG00000238889	0	0	0
ENSG00000238891	0	0	0
ENSG00000238891	0	0	0
ENSG00000238892	0	0	0
ENSG00000238893	0	0	0
ENSG00000238895	0	0	0
ENSG00000238896	0	0	0
ENSG00000238899	0	0	0
ENSG00000238899	0	0	0
ENSG00000238900	0	0	0
ENSG00000238901	0	0	0
ENSG00000238902	0	0	0
ENSG00000238905	0	0	0
ENSG00000238906	0	0	0
ENSG00000238907	0	0	0
ENSG00000238910	0	0	0
ENSG00000238914	0	0	0
ENSG00000238914	0	0	0
ENSG00000238918	0	0	0
ENSG00000238920	0	0	0
ENSG00000238920	0	0	0
ENSG00000238922	0	0	0
ENSG00000238925	0	0	0
ENSG00000238925	0	0	0
ENSG00000238929	0	0	0
ENSG00000238931	0	0	0
ENSG00000238932	0	0	0
ENSG00000238934	0	0	0
ENSG00000238935	0	0	0
ENSG00000238936	0	0	0
ENSG00000238938	0	0	0
ENSG00000238939	0	0	0
ENSG00000238940	0	0	0
ENSG00000238940	0	0	0
ENSG00000238945	0	0	0
ENSG00000238946	0	0	0
ENSG238947	0	0	0

ENSG239047	0	0	0
ENSG00000238948	0	0	0
ENSG00000238951	0	0	0
ENSG00000238951	0	0	0
ENSG00000238954	0	0	0
ENSG00000238960	0	0	0
ENSG00000238963	0	0	0
ENSG00000238966	0	0	0
ENSG00000238968	0	0	0
ENSG00000238969	0	0	0
ENSG00000238970	0	0	0
ENSG00000238970	0	0	0
ENSG00000238972	0	0	0
ENSG00000238972	0	0	0
ENSG00000238974	0	0	0
ENSG00000238975	0	0	0
ENSG00000238978	0	0	0
ENSG00000238979	0	0	0
ENSG00000238981	0	0	0
ENSG00000238981	0	0	0
ENSG00000238982	0	0	0
ENSG00000238983	0	0	0
ENSG00000238983	0	0	0
ENSG00000238984	0	0	0
ENSG00000238985	0	0	0
ENSG00000238986	0	0	0
ENSG00000238986	0	0	0
ENSG00000238988	0	0	0
ENSG00000238991	0	0	0
ENSG00000238992	0	0	0
ENSG00000238995	0	0	0
ENSG00000238995	0	0	0
ENSG00000238996	0	0	0
ENSG00000238999	0	0	0
ENSG00000238999	0	0	0
ENSG00000239000	0	0	0
ENSG00000239005	0	0	0
ENSG00000239005	0	0	0
ENSG00000239008	0	0	0
ENSG00000239008	0	0	0
ENSG00000239011	0	0	0
ENSG00000239011	0	0	0
ENSG00000239013	0	0	0
ENSG00000239015	0	0	0
ENSG00000239017	0	0	0
ENSG00000239018	0	0	0
ENSG00000239018	0	0	0
ENSG00000239020	0	0	0
ENSG00000239020	0	0	0
ENSG00000239024	0	0	0
ENSG00000239025	0	0	0
ENSG239026	0	0	0

ENSG239027	0	0	0
ENSG00000239031	0	0	0
ENSG00000239033	0	0	0
ENSG00000239033	0	0	0
ENSG00000239034	0	0	0
ENSG00000239034	0	0	0
ENSG00000239035	0	0	0
ENSG00000239037	0	0	0
ENSG00000239038	0	0	0
ENSG00000239041	0	0	0
ENSG00000239041	0	0	0
ENSG00000239044	0	0	0
ENSG00000239045	0	0	0
ENSG00000239046	0	0	0
ENSG00000239049	0	0	0
ENSG00000239049	0	0	0
ENSG00000239052	0	0	0
ENSG00000239052	0	0	0
ENSG00000239054	0	0	0
ENSG00000239055	0	0	0
ENSG00000239056	0	0	0
ENSG00000239058	0	0	0
ENSG00000239059	0	0	0
ENSG00000239059	0	0	0
ENSG00000239061	0	0	0
ENSG00000239063	0	0	0
ENSG00000239063	0	0	0
ENSG00000239064	0	0	0
ENSG00000239065	0	0	0
ENSG00000239066	0	0	0
ENSG00000239066	0	0	0
ENSG00000239067	0	0	0
ENSG00000239068	0	0	0
ENSG00000239072	0	0	0
ENSG00000239073	0	0	0
ENSG00000239077	0	0	0
ENSG00000239079	0	0	0
ENSG00000239080	0	0	0
ENSG00000239080	0	0	0
ENSG00000239083	0	0	0
ENSG00000239084	0	0	0
ENSG00000239084	0	0	0
ENSG00000239086	0	0	0
ENSG00000239087	0	0	0
ENSG00000239089	0	0	0
ENSG00000239091	0	0	0
ENSG00000239093	0	0	0
ENSG00000239094	0	0	0
ENSG00000239094	0	0	0
ENSG00000239095	0	0	0
ENSG00000239096	0	0	0
ENSG00000239098	0	0	0

ENSG00000239098	0	0	0
ENSG00000239100	0	0	0
ENSG00000239103	0	0	0
ENSG00000239111	0	0	0
ENSG00000239114	0	0	0
ENSG00000239116	0	0	0
ENSG00000239121	0	0	0
ENSG00000239123	0	0	0
ENSG00000239125	0	0	0
ENSG00000239126	0	0	0
ENSG00000239128	0	0	0
ENSG00000239129	0	0	0
ENSG00000239130	0	0	0
ENSG00000239130	0	0	0
ENSG00000239132	0	0	0
ENSG00000239133	0	0	0
ENSG00000239134	0	0	0
ENSG00000239134	0	0	0
ENSG00000239135	0	0	0
ENSG00000239136	0	0	0
ENSG00000239137	0	0	0
ENSG00000239140	0	0	0
ENSG00000239140	0	0	0
ENSG00000239141	0	0	0
ENSG00000239142	0	0	0
ENSG00000239144	0	0	0
ENSG00000239144	0	0	0
ENSG00000239145	0	0	0
ENSG00000239146	0	0	0
ENSG00000239148	0	0	0
ENSG00000239153	0	0	0
ENSG00000239154	0	0	0
ENSG00000239154	0	0	0
ENSG00000239155	0	0	0
ENSG00000239157	0	0	0
ENSG00000239157	0	0	0
ENSG00000239159	0	0	0
ENSG00000239159	0	0	0
ENSG00000239161	0	0	0
ENSG00000239166	0	0	0
ENSG00000239170	0	0	0
ENSG00000239171	0	0	0
ENSG00000239172	0	0	0
ENSG00000239173	0	0	0
ENSG00000239176	0	0	0
ENSG00000239182	0	0	0
ENSG00000239186	0	0	0
ENSG00000239188	0	0	0
ENSG00000239188	0	0	0
ENSG00000239191	0	0	0
ENSG00000239191	0	0	0
ENSG00000239193	0	0	0

ENSG00000239197	0	0	0
ENSG00000251699	0	0	0
ENSG00000251700	0	0	0
ENSG00000251704	0	0	0
ENSG00000251709	0	0	0
ENSG00000251715	0	0	0
ENSG00000251721	0	0	0
ENSG00000251730	0	0	0
ENSG00000251735	0	0	0
ENSG00000251737	0	0	0
ENSG00000251740	0	0	0
ENSG00000251744	0	0	0
ENSG00000251749	0	0	0
ENSG00000251762	0	0	0
ENSG00000251769	0	0	0
ENSG00000251775	0	0	0
ENSG00000251778	0	0	0
ENSG00000251778	0	0	0
ENSG00000251793	0	0	0
ENSG00000251795	0	0	0
ENSG00000251796	0	0	0
ENSG00000251800	0	0	0
ENSG00000251801	0	0	0
ENSG00000251802	0	0	0
ENSG00000251805	0	0	0
ENSG00000251817	0	0	0
ENSG00000251818	0	0	0
ENSG00000251822	0	0	0
ENSG00000251824	0	0	0
ENSG00000251828	0	0	0
ENSG00000251830	0	0	0
ENSG00000251833	0	0	0
ENSG00000251836	0	0	0
ENSG00000251838	0	0	0
ENSG00000251844	0	0	0
ENSG00000251846	0	0	0
ENSG00000251847	0	0	0
ENSG00000251848	0	0	0
ENSG00000251858	0	0	0
ENSG00000251860	0	0	0
ENSG00000251861	0	0	0
ENSG00000251861	0	0	0
ENSG00000251863	0	0	0
ENSG00000251866	0	0	0
ENSG00000251866	0	0	0
ENSG00000251878	0	0	0
ENSG00000251881	0	0	0
ENSG00000251893	0	0	0
ENSG00000251901	0	0	0
ENSG00000251909	0	0	0
ENSG00000251911	0	0	0
ENSG00000251918	0	0	0

ENSG00000251922	0	0	0
ENSG00000251925	0	0	0
ENSG00000251926	0	0	0
ENSG00000251930	0	0	0
ENSG00000251938	0	0	0
ENSG00000251938	0	0	0
ENSG00000251940	0	0	0
ENSG00000251942	0	0	0
ENSG00000251944	0	0	0
ENSG00000251949	0	0	0
ENSG00000251959	0	0	0
ENSG00000251967	0	0	0
ENSG00000251974	0	0	0
ENSG00000251974	0	0	0
ENSG00000251974	0	0	0
ENSG00000251979	0	0	0
ENSG00000251987	0	0	0
ENSG00000251992	0	0	0
ENSG00000251999	0	0	0
ENSG00000252000	0	0	0
ENSG00000252009	0	0	0
ENSG00000252011	0	0	0
ENSG00000252014	0	0	0
ENSG00000252016	0	0	0
ENSG00000252020	0	0	0
ENSG00000252022	0	0	0
ENSG00000252024	0	0	0
ENSG00000252040	0	0	0
ENSG00000252040	0	0	0
ENSG00000252045	0	0	0
ENSG00000252048	0	0	0
ENSG00000252049	0	0	0
ENSG00000252050	0	0	0
ENSG00000252054	0	0	0
ENSG00000252058	0	0	0
ENSG00000252058	0	0	0
ENSG00000252071	0	0	0
ENSG00000252077	0	0	0
ENSG00000252078	0	0	0
ENSG00000252083	0	0	0
ENSG00000252088	0	0	0
ENSG00000252096	0	0	0
ENSG00000252102	0	0	0
ENSG00000252109	0	0	0
ENSG00000252110	0	0	0
ENSG00000252112	0	0	0
ENSG00000252114	0	0	0
ENSG00000252119	0	0	0
ENSG00000252122	0	0	0
ENSG00000252127	0	0	0
ENSG00000252128	0	0	0
ENSG00000252129	0	0	0

ENSG00000252133	0	0	0
ENSG00000252136	0	0	0
ENSG00000252138	0	0	0
ENSG00000252140	0	0	0
ENSG00000252143	0	0	0
ENSG00000252144	0	0	0
ENSG00000252154	0	0	0
ENSG00000252154	0	0	0
ENSG00000252158	0	0	0
ENSG00000252158	0	0	0
ENSG00000252170	0	0	0
ENSG00000252170	0	0	0
ENSG00000252175	0	0	0
ENSG00000252188	0	0	0
ENSG00000252189	0	0	0
ENSG00000252190	0	0	0
ENSG00000252192	0	0	0
ENSG00000252193	0	0	0
ENSG00000252199	0	0	0
ENSG00000252200	0	0	0
ENSG00000252203	0	0	0
ENSG00000252204	0	0	0
ENSG00000252213	0	0	0
ENSG00000252218	0	0	0
ENSG00000252218	0	0	0
ENSG00000252227	0	0	0
ENSG00000252228	0	0	0
ENSG00000252230	0	0	0
ENSG00000252236	0	0	0
ENSG00000252236	0	0	0
ENSG00000252238	0	0	0
ENSG00000252241	0	0	0
ENSG00000252241	0	0	0
ENSG00000252249	0	0	0
ENSG00000252256	0	0	0
ENSG00000252258	0	0	0
ENSG00000252258	0	0	0
ENSG00000252265	0	0	0
ENSG00000252274	0	0	0
ENSG00000252277	0	0	0
ENSG00000252277	0	0	0
ENSG00000252281	0	0	0
ENSG00000252284	0	0	0
ENSG00000252284	0	0	0
ENSG00000252290	0	0	0
ENSG00000252291	0	0	0
ENSG00000252291	0	0	0
ENSG00000252295	0	0	0
ENSG00000252296	0	0	0
ENSG00000252298	0	0	0
ENSG00000252300	0	0	0
ENSG00000252305	0	0	0

ENSG00000252314	0	0	0
ENSG00000252329	0	0	0
ENSG00000252329	0	0	0
ENSG00000252337	0	0	0
ENSG00000252349	0	0	0
ENSG00000252352	0	0	0
ENSG00000252352	0	0	0
ENSG00000252354	0	0	0
ENSG00000252356	0	0	0
ENSG00000252356	0	0	0
ENSG00000252359	0	0	0
ENSG00000252365	0	0	0
ENSG00000252372	0	0	0
ENSG00000252380	0	0	0
ENSG00000252388	0	0	0
ENSG00000252392	0	0	0
ENSG00000252402	0	0	0
ENSG00000252404	0	0	0
ENSG00000252405	0	0	0
ENSG00000252408	0	0	0
ENSG00000252409	0	0	0
ENSG00000252425	0	0	0
ENSG00000252427	0	0	0
ENSG00000252433	0	0	0
ENSG00000252433	0	0	0
ENSG00000252434	0	0	0
ENSG00000252435	0	0	0
ENSG00000252438	0	0	0
ENSG00000252440	0	0	0
ENSG00000252441	0	0	0
ENSG00000252443	0	0	0
ENSG00000252447	0	0	0
ENSG00000252448	0	0	0
ENSG00000252458	0	0	0
ENSG00000252459	0	0	0
ENSG00000252461	0	0	0
ENSG00000252461	0	0	0
ENSG00000252473	0	0	0
ENSG00000252476	0	0	0
ENSG00000252495	0	0	0
ENSG00000252502	0	0	0
ENSG00000252505	0	0	0
ENSG00000252505	0	0	0
ENSG00000252517	0	0	0
ENSG00000252525	0	0	0
ENSG00000252526	0	0	0
ENSG00000252526	0	0	0
ENSG00000252529	0	0	0
ENSG00000252531	0	0	0
ENSG00000252536	0	0	0
ENSG00000252537	0	0	0
ENSG00000252543	1	0	0

ENSG00000252550	0	0	0
ENSG00000252557	0	0	0
ENSG00000252557	0	0	0
ENSG00000252559	0	0	0
ENSG00000252565	0	0	0
ENSG00000252566	0	0	0
ENSG00000252571	0	0	0
ENSG00000252572	0	0	0
ENSG00000252576	0	0	0
ENSG00000252580	0	0	0
ENSG00000252582	0	0	0
ENSG00000252592	0	0	0
ENSG00000252601	0	0	0
ENSG00000252602	0	0	0
ENSG00000252605	0	0	0
ENSG00000252609	0	0	0
ENSG00000252617	0	0	0
ENSG00000252638	0	0	0
ENSG00000252640	0	0	0
ENSG00000252646	0	0	0
ENSG00000252646	0	0	0
ENSG00000252657	0	0	0
ENSG00000252657	0	0	0
ENSG00000252668	0	0	0
ENSG00000252668	0	0	0
ENSG00000252669	0	0	0
ENSG00000252672	0	0	0
ENSG00000252677	0	0	0
ENSG00000252679	0	0	0
ENSG00000252682	0	0	0
ENSG00000252683	0	0	0
ENSG00000252687	0	0	0
ENSG00000252689	0	0	0
ENSG00000252691	0	0	0
ENSG00000252692	0	0	0
ENSG00000252693	0	0	0
ENSG00000252699	0	0	0
ENSG00000252701	0	0	0
ENSG00000252701	0	0	0
ENSG00000252706	0	0	0
ENSG00000252709	0	0	0
ENSG00000252719	0	0	0
ENSG00000252722	0	0	0
ENSG00000252722	0	0	0
ENSG00000252724	0	0	0
ENSG00000252727	0	0	0
ENSG00000252727	0	0	0
ENSG00000252728	0	0	0
ENSG00000252740	0	0	0
ENSG00000252762	0	0	0
ENSG00000252765	0	0	0
ENSG252765	0	0	0

ENSG00000252771	0	0	0
ENSG00000252774	0	0	0
ENSG00000252777	0	0	0
ENSG00000252778	0	0	0
ENSG00000252787	0	0	0
ENSG00000252790	0	0	0
ENSG00000252792	0	0	0
ENSG00000252792	0	0	0
ENSG00000252798	0	0	0
ENSG00000252798	0	0	0
ENSG00000252798	0	0	0
ENSG00000252799	0	0	0
ENSG00000252800	0	0	0
ENSG00000252805	0	0	0
ENSG00000252824	0	0	0
ENSG00000252829	0	0	0
ENSG00000252829	0	0	0
ENSG00000252834	0	0	0
ENSG00000252840	0	0	0
ENSG00000252840	0	0	0
ENSG00000252840	0	0	0
ENSG00000252844	0	0	0
ENSG00000252849	0	0	0
ENSG00000252852	0	0	0
ENSG00000252853	0	0	0
ENSG00000252868	0	0	0
ENSG00000252870	0	0	0
ENSG00000252873	0	0	0
ENSG00000252878	0	0	0
ENSG00000252883	0	0	0
ENSG00000252883	0	0	0
ENSG00000252888	0	0	0
ENSG00000252904	0	0	0
ENSG00000252917	0	0	0
ENSG00000252920	0	0	0
ENSG00000252921	0	0	0
ENSG00000252921	0	0	0
ENSG00000252923	0	0	0
ENSG00000252923	0	0	0
ENSG00000252932	0	0	0
ENSG00000252945	0	0	0
ENSG00000252946	0	0	0
ENSG00000252961	0	0	0
ENSG00000252969	0	0	0
ENSG00000252981	0	0	0
ENSG00000252985	0	0	0
ENSG00000252989	0	0	0
ENSG00000252992	0	0	0
ENSG00000252992	0	0	0
ENSG00000252993	0	0	0
ENSG00000253004	0	0	0
ENSG253007	0	0	0

ENSG00000253009	0	0	0
ENSG00000253013	0	0	0
ENSG00000253014	0	0	0
ENSG00000253014	0	0	0
ENSG00000253027	0	0	0
ENSG00000253028	0	0	0
ENSG00000253036	0	0	0
ENSG00000253041	0	0	0
ENSG00000253042	0	0	0
ENSG00000253042	0	0	0
ENSG00000253047	0	0	0
ENSG00000253047	0	0	0
ENSG00000253049	0	0	0
ENSG00000253051	0	0	0
ENSG00000253052	0	0	0
ENSG00000253059	0	0	0
ENSG00000253060	0	0	0
ENSG00000253065	0	0	0
ENSG00000253067	0	0	0
ENSG00000253068	0	0	0
ENSG00000253072	0	0	0
ENSG00000253076	0	0	0
ENSG00000253085	0	0	0
ENSG00000253090	0	0	0
ENSG00000253090	0	0	0
ENSG00000253091	0	0	0
ENSG00000253092	0	0	0
ENSG00000253094	0	0	0
ENSG00000261955	0	0	0
ENSG00000261990	0	0	0
ENSG00000262064	0	0	0
ENSG00000262205	0	0	0
ENSG00000262240	0	0	0
ENSG00000262432	0	0	0
ENSG00000262522	0	0	0
ENSG00000262620	0	0	0
ENSG00000262698	0	0	0
ENSG00000262778	0	0	0
ENSG00000263358	0	0	0
ENSG00000263359	0	0	0
ENSG00000263362	0	0	0
ENSG00000263442	0	0	0
ENSG00000263486	0	0	0
ENSG00000263495	0	0	0
ENSG00000263517	0	0	0
ENSG00000263589	0	0	0
ENSG00000263625	0	0	0
ENSG00000263695	0	0	0
ENSG00000263723	0	0	0
ENSG00000263864	0	0	0
ENSG00000263879	0	0	0
ENSG00000263942	0	0	0

ENSG00000263994	0	0	0
ENSG00000264086	0	0	0
ENSG00000264153	0	0	0
ENSG00000264202	0	0	0
ENSG00000264346	0	0	0
ENSG00000264346	0	0	0
ENSG00000264351	0	0	0
ENSG00000264379	0	0	0
ENSG00000264452	0	0	0
ENSG00000264466	0	0	0
ENSG00000264591	0	0	0
ENSG00000264997	0	0	0
ENSG00000265013	0	0	0
ENSG00000265116	0	0	0
ENSG00000265138	0	0	0
ENSG00000265156	0	0	0
ENSG00000265183	0	0	0
ENSG00000265325	0	0	0
ENSG00000265431	0	0	0
ENSG00000265495	0	0	0
ENSG00000265602	0	0	0
ENSG00000265607	0	0	0
ENSG00000265634	0	0	0
ENSG00000265651	0	0	0
ENSG00000265663	0	0	0
ENSG00000265674	0	0	0
ENSG00000265706	0	0	0
ENSG00000265707	0	0	0
ENSG00000265721	0	0	0
ENSG00000265732	0	0	0
ENSG00000265733	0	0	0
ENSG00000265754	0	0	0
ENSG00000265849	0	0	0
ENSG00000265926	0	0	0
ENSG00000265941	0	0	0
ENSG00000266004	0	0	0
ENSG00000266132	0	0	0
ENSG00000266284	0	0	0
ENSG00000266295	0	0	0
ENSG00000266300	0	0	0
ENSG00000266419	0	0	0
ENSG00000266646	0	0	0
ENSG00000266672	0	0	0
ENSG00000266692	0	0	0
ENSG00000266692	0	0	0
ENSG00000266755	0	0	0
ENSG00000266834	0	0	0
ENSG00000266847	0	0	0
ENSG00000267818	0	0	0
ENSG00000267933	0	0	0
ENSG00000268063	0	0	0
ENSG00000268073	0	0	0

ENSG00000268145	0	0	0
ENSG00000268237	0	0	0
ENSG00000268261	0	0	0
ENSG00000268305	0	0	0
ENSG00000268369	0	0	0
ENSG00000268513	0	0	0
ENSG00000268874	0	0	0
ENSG00000268890	0	0	0
ENSG00000268911	0	0	0
ENSG00000269023	0	0	0
ENSG00000269160	0	0	0
ENSG00000269368	0	0	0
ENSG00000269629	0	0	0
ENSG00000269689	0	0	0
ENSG00000269874	0	0	0
HBI-100	0	0	0
HBI-115	0	0	0
HBI-36	0	0	0
HBI-43	0	0	0
HBI-61	0	0	0
HBI-61	0	0	0
HBI-61	0	0	0
HBI-6	0	0	0
HBII-108B	0	0	0
HBII-108	0	0	0
HBII-115	0	0	0
HBII-135	0	0	0
HBII-13	0	0	0
HBII-13	0	0	0
HBII-142	0	0	0
HBII-142	0	0	0
HBII-166	0	0	0
HBII-180A	0	0	0
HBII-180B	0	0	0
HBII-180C	0	0	0
HBII-202	0	0	0
HBII-210	0	0	0
HBII-234	0	0	0
HBII-234	0	0	0
HBII-239	0	0	0
HBII-240	1	1	0
HBII-251	0	0	0
HBII-276	0	0	0
HBII-289	0	0	0
HBII-295	0	0	0
HBII-296A	0	0	0
HBII-296A	0	0	0
HBII-296B	0	0	0
HBII-296B	0	0	0
HBII-316	0	0	0
HBII-336	0	0	0
HBII-382	0	0	0

HBII-419	0	0	0
HBII-420	0	0	0
HBII-429	0	0	0
HBII-436	0	0	0
HBII-437	0	0	0
HBII-438A	0	0	0
HBII-438B	0	0	0
HBII-52-10	0	0	0
HBII-52-11	0	0	0
HBII-52-12	0	0	0
HBII-52-13	0	0	0
HBII-52-14	0	0	0
HBII-52-15	0	1	0
HBII-52-16	0	0	0
HBII-52-17	0	0	0
HBII-52-18	0	0	0
HBII-52-19	0	0	0
HBII-52-1	0	0	0
HBII-52-20	0	0	0
HBII-52-21	0	0	0
HBII-52-22	0	0	0
HBII-52-23	0	0	0
HBII-52-23	0	0	0
HBII-52-25	0	0	0
HBII-52-26	0	0	0
HBII-52-29	0	0	0
HBII-52-2	0	0	0
HBII-52-30	0	0	0
HBII-52-31	0	0	0
HBII-52-32	0	0	0
HBII-52-33	0	0	0
HBII-52-34	0	0	0
HBII-52-35	0	0	0
HBII-52-36	0	0	0
HBII-52-37	0	0	0
HBII-52-37	0	0	0
HBII-52-38	0	0	0
HBII-52-39	0	0	0
HBII-52-3	0	0	0
HBII-52-40	0	0	0
HBII-52-41	0	0	0
HBII-52-42	0	0	0
HBII-52-43	0	0	0
HBII-52-44	0	0	0
HBII-52-48	0	0	0
HBII-52-48	0	0	0
HBII-52-4	0	0	0
HBII-52-5	0	0	0
HBII-52-6	0	0	0
HBII-52-7	0	0	0
HBII-52-8	0	0	0
HBII-52-9	0	0	0

HBII-55	0	0	0
HBII-82B	0	0	0
HBII-82	0	0	0
HBII-85-10	0	0	0
HBII-85-11	0	0	0
HBII-85-11	0	0	0
HBII-85-12	0	0	0
HBII-85-13	0	0	0
HBII-85-14	0	0	0
HBII-85-15	0	0	0
HBII-85-16	0	0	0
HBII-85-17	0	0	0
HBII-85-18	0	0	0
HBII-85-19	0	0	0
HBII-85-1	0	0	0
HBII-85-20	0	0	0
HBII-85-21	0	0	0
HBII-85-22	0	0	0
HBII-85-23	0	0	0
HBII-85-24	0	0	0
HBII-85-25	0	0	0
HBII-85-26	0	0	0
HBII-85-26	0	0	0
HBII-85-26	0	0	0
HBII-85-27	0	0	0
HBII-85-28	0	0	0
HBII-85-29	0	0	0
HBII-85-2	0	0	0
HBII-85-3	0	0	0
HBII-85-4	0	0	0
HBII-85-5	0	0	0
HBII-85-6	0	0	0
HBII-85-7	0	0	0
HBII-85-8	0	0	0
HBII-85-9	0	0	0
HBII-95B	0	0	0
HBII-95	0	0	0
HBII-95	0	0	0
HBII-99B	0	0	0
HBII-99	0	0	0
hsa-let-7a-1	0	0	0
hsa-let-7a-1	0	0	0
hsa-let-7a-2	0	0	0
hsa-let-7a-3	0	0	0
hsa-let-7b	0	0	0
hsa-let-7b	0	0	0
hsa-let-7c	0	0	0
hsa-let-7d	0	0	0
hsa-let-7e	0	0	0
hsa-let-7f-1	0	0	0
hsa-let-7f-1	0	0	0
hsa-let-7f-2	0	0	0

hsa-let-7f-2	0	0	0
hsa-mir-15a	0	0	0
hsa-mir-16-1	0	0	0
hsa-mir-17	0	0	0
hsa-mir-18a	0	0	0
hsa-mir-18a	0	0	0
hsa-mir-19a	0	0	0
hsa-mir-19b-1	0	0	0
hsa-mir-19b-1	0	0	0
hsa-mir-19b-2	0	0	0
hsa-mir-20a	0	0	0
hsa-mir-21	0	0	0
hsa-mir-22	0	0	0
hsa-mir-23a	0	0	0
hsa-mir-24-1	0	0	0
hsa-mir-24-1	0	0	0
hsa-mir-24-2	0	0	0
hsa-mir-24-2	0	0	0
hsa-mir-25	0	0	0
hsa-mir-26a-1	0	0	0
hsa-mir-26a-1	0	0	0
hsa-mir-26b	0	0	0
hsa-mir-27a	0	0	0
hsa-mir-27a	0	0	0
hsa-mir-28	0	0	0
hsa-mir-29a	0	0	0
hsa-mir-29a	0	0	0
hsa-mir-30a	0	0	0
hsa-mir-30a	0	0	0
hsa-mir-31	0	0	0
hsa-mir-32	0	0	0
hsa-mir-33a	0	0	0
hsa-mir-92a-1	0	0	0
hsa-mir-92a-1	0	0	0
hsa-mir-92a-2	0	0	0
hsa-mir-92a-2	0	0	0
hsa-mir-93	0	0	0
hsa-mir-95	0	0	0
hsa-mir-96	0	0	0
hsa-mir-98	0	0	0
hsa-mir-99a	0	0	0
hsa-mir-100	0	0	0
hsa-mir-101-1	0	0	0
hsa-mir-101-1	0	0	0
hsa-mir-29b-1	0	0	0
hsa-mir-29b-1	0	0	0
hsa-mir-29b-2	0	0	0
hsa-mir-29b-2	0	0	0
hsa-mir-103a-2	0	0	0
hsa-mir-103a-2	0	0	0
hsa-mir-103a-1	0	0	0
hsa-mir-103a-1	0	0	0

hsa-mir-105-1	0	0	0
hsa-mir-105-2	0	0	0
hsa-mir-106a	0	0	0
hsa-mir-107	0	0	0
hsa-mir-16-2	0	0	0
hsa-mir-16-2	0	0	0
hsa-mir-192	0	0	0
hsa-mir-196a-1	0	0	0
hsa-mir-197	0	0	0
hsa-mir-198	0	0	0
hsa-mir-199a-1	0	0	0
hsa-mir-199a-1	0	0	0
hsa-mir-208a	0	0	0
hsa-mir-129-1	0	0	0
hsa-mir-129-1	0	0	0
hsa-mir-148a	0	0	0
hsa-mir-30c-2	0	0	0
hsa-mir-30d	0	0	0
hsa-mir-139	0	0	0
hsa-mir-147a	0	0	0
hsa-mir-147a	0	0	0
hsa-mir-7-1	0	0	0
hsa-mir-7-2	0	0	0
hsa-mir-7-3	0	0	0
hsa-mir-10a	0	0	0
hsa-mir-10b	0	0	0
hsa-mir-34a	0	0	0
hsa-mir-181a-2	0	0	0
hsa-mir-181b-1	0	0	0
hsa-mir-181c	0	0	0
hsa-mir-182	0	0	0
hsa-mir-183	0	0	0
hsa-mir-187	0	0	0
hsa-mir-196a-2	0	0	0
hsa-mir-199a-2	0	0	0
hsa-mir-199b	0	0	0
hsa-mir-203a	0	0	0
hsa-mir-204	0	0	0
hsa-mir-205	0	0	0
hsa-mir-210	0	0	0
hsa-mir-211	0	0	0
hsa-mir-212	0	0	0
hsa-mir-181a-1	0	0	0
hsa-mir-181a-1	0	0	0
hsa-mir-214	0	0	0
hsa-mir-215	0	0	0
hsa-mir-216a	0	0	0
hsa-mir-217	0	0	0
hsa-mir-218-1	0	0	0
hsa-mir-218-2	0	0	0
hsa-mir-219a-1	0	0	0
hsa-mir-221	0	0	0

hsa-mir-222	0	0	0
hsa-mir-223	0	0	0
hsa-mir-224	0	0	0
hsa-mir-200b	0	0	0
hsa-let-7g	0	0	0
hsa-let-7i	0	0	0
hsa-mir-1-2	0	0	0
hsa-mir-1-2	0	0	0
hsa-mir-15b	0	0	0
hsa-mir-23b	0	0	0
hsa-mir-27b	0	0	0
hsa-mir-30b	0	0	0
hsa-mir-122	0	0	0
hsa-mir-124-1	0	0	0
hsa-mir-124-1	0	0	0
hsa-mir-124-1	0	0	0
hsa-mir-124-2	0	0	0
hsa-mir-124-2	0	0	0
hsa-mir-124-3	0	0	0
hsa-mir-124-3	0	0	0
hsa-mir-125b-1	0	0	0
hsa-mir-125b-1	0	0	0
hsa-mir-128-1	0	0	0
hsa-mir-130a	0	0	0
hsa-mir-132	0	0	0
hsa-mir-133a-1	0	0	0
hsa-mir-133a-1	0	0	0
hsa-mir-133a-1	0	0	0
hsa-mir-133a-2	0	0	0
hsa-mir-133a-2	0	0	0
hsa-mir-133a-2	0	0	0
hsa-mir-135a-1	0	0	0
hsa-mir-135a-2	0	0	0
hsa-mir-137	0	0	0
hsa-mir-138-2	0	0	0
hsa-mir-138-2	0	0	0
hsa-mir-140	0	0	0
hsa-mir-141	0	0	0
hsa-mir-142	0	0	0
hsa-mir-143	0	0	0
hsa-mir-144	0	0	0
hsa-mir-145	0	0	0
hsa-mir-152	0	0	0
hsa-mir-153-1	0	0	0
hsa-mir-153-1	0	0	0
hsa-mir-153-2	0	0	0
hsa-mir-153-2	0	0	0
hsa-mir-191	0	0	0
hsa-mir-9-1	0	0	0
hsa-mir-9-1	0	0	0
hsa-mir-9-2	0	0	0
hsa-mir-9-2	0	0	0

hsa-mir-9-3	0	0	0
hsa-mir-125a	0	0	0
hsa-mir-125b-2	0	0	0
hsa-mir-126	0	0	0
hsa-mir-127	0	0	0
hsa-mir-129-2	0	0	0
hsa-mir-134	0	0	0
hsa-mir-136	0	0	0
hsa-mir-138-1	0	0	0
hsa-mir-146a	0	0	0
hsa-mir-149	0	0	0
hsa-mir-150	0	0	0
hsa-mir-154	0	0	0
hsa-mir-184	0	0	0
hsa-mir-185	0	0	0
hsa-mir-186	0	0	0
hsa-mir-188	0	0	0
hsa-mir-190a	0	0	0
hsa-mir-190a	0	0	0
hsa-mir-193a	0	0	0
hsa-mir-194-1	0	0	0
hsa-mir-195	0	0	0
hsa-mir-206	0	0	0
hsa-mir-320a	0	0	0
hsa-mir-200c	0	0	0
hsa-mir-1-1	0	0	0
hsa-mir-1-1	0	0	0
hsa-mir-155	0	0	0
hsa-mir-181b-2	0	0	0
hsa-mir-128-2	0	0	0
hsa-mir-194-2	0	0	0
hsa-mir-106b	0	0	0
hsa-mir-29c	0	0	0
hsa-mir-30c-1	0	0	0
hsa-mir-200a	0	0	0
hsa-mir-302a	0	0	0
hsa-mir-101-2	0	0	0
hsa-mir-101-2	0	0	0
hsa-mir-219a-2	0	0	0
hsa-mir-34b	0	0	0
hsa-mir-34c	0	0	0
hsa-mir-299	0	0	0
hsa-mir-301a	0	0	0
hsa-mir-301a	0	0	0
hsa-mir-99b	0	0	0
hsa-mir-296	0	0	0
hsa-mir-130b	0	0	0
hsa-mir-30e	0	0	0
hsa-mir-26a-2	0	0	0
hsa-mir-361	0	0	0
hsa-mir-362	0	0	0
hsa-mir-363	0	0	0

hsa-mir-365a	0	0	0
hsa-mir-365a	0	0	0
hsa-mir-365b	0	0	0
hsa-mir-302b	0	0	0
hsa-mir-302b	0	0	0
hsa-mir-302c	0	0	0
hsa-mir-302d	0	0	0
hsa-mir-367	0	0	0
hsa-mir-376c	0	0	0
hsa-mir-369	0	0	0
hsa-mir-370	0	0	0
hsa-mir-371a	0	0	0
hsa-mir-372	0	0	0
hsa-mir-373	0	0	0
hsa-mir-374a	0	0	0
hsa-mir-375	0	0	0
hsa-mir-376a-1	0	0	0
hsa-mir-376a-1	0	0	0
hsa-mir-377	0	0	0
hsa-mir-378a	0	0	0
hsa-mir-379	0	0	0
hsa-mir-380	0	0	0
hsa-mir-381	0	0	0
hsa-mir-382	0	0	0
hsa-mir-383	0	0	0
hsa-mir-340	0	0	0
hsa-mir-330	0	0	0
hsa-mir-328	0	0	0
hsa-mir-342	0	0	0
hsa-mir-337	0	0	0
hsa-mir-323a	0	0	0
hsa-mir-326	0	0	0
hsa-mir-151a	0	0	0
hsa-mir-135b	0	0	0
hsa-mir-148b	0	0	0
hsa-mir-331	0	0	0
hsa-mir-324	0	0	0
hsa-mir-338	0	0	0
hsa-mir-339	0	0	0
hsa-mir-335	0	0	0
hsa-mir-133b	0	0	0
hsa-mir-325	0	0	0
hsa-mir-345	0	0	0
hsa-mir-346	0	0	0
hsa-mir-384	0	0	0
hsa-mir-196b	0	0	0
hsa-mir-422a	0	0	0
hsa-mir-423	0	0	0
hsa-mir-424	0	0	0
hsa-mir-425	0	0	0
hsa-mir-18b	0	0	0
hsa-mir-20b	0	0	0

hsa-mir-448	0	0	0
hsa-mir-429	0	0	0
hsa-mir-449a	0	0	0
hsa-mir-450a-1	0	0	0
hsa-mir-450a-1	0	0	0
hsa-mir-431	0	0	0
hsa-mir-433	0	0	0
hsa-mir-329-1	0	0	0
hsa-mir-329-2	0	0	0
hsa-mir-451a	0	0	0
hsa-mir-452	0	0	0
hsa-mir-409	0	0	0
hsa-mir-412	0	0	0
hsa-mir-410	0	0	0
hsa-mir-376b	0	0	0
hsa-mir-376b	0	0	0
hsa-mir-483	0	0	0
hsa-mir-484	0	0	0
hsa-mir-485	0	0	0
hsa-mir-486	0	0	0
hsa-mir-486	0	0	0
hsa-mir-487a	0	0	0
hsa-mir-488	0	0	0
hsa-mir-489	0	0	0
hsa-mir-490	0	0	0
hsa-mir-491	0	0	0
hsa-mir-511	0	0	0
hsa-mir-146b	0	0	0
hsa-mir-202	0	0	0
hsa-mir-492	0	0	0
hsa-mir-493	0	0	0
hsa-mir-432	0	0	0
hsa-mir-494	0	0	0
hsa-mir-495	0	0	0
hsa-mir-496	0	0	0
hsa-mir-193b	0	0	0
hsa-mir-497	0	0	0
hsa-mir-181d	0	0	0
hsa-mir-181d	0	0	0
hsa-mir-512-1	0	0	0
hsa-mir-512-2	0	0	0
hsa-mir-498	0	0	0
hsa-mir-520e	0	0	0
hsa-mir-515-1	0	0	0
hsa-mir-519e	0	0	0
hsa-mir-520f	0	0	0
hsa-mir-520f	0	0	0
hsa-mir-515-2	0	0	0
hsa-mir-519c	0	0	0
hsa-mir-520a	0	0	0
hsa-mir-526b	0	0	0
hsa-mir-519b	0	0	0

hsa-mir-525	0	0	0
hsa-mir-525	0	0	0
hsa-mir-523	0	0	0
hsa-mir-523	0	0	0
hsa-mir-518f	0	0	0
hsa-mir-520b	0	0	0
hsa-mir-518b	0	0	0
hsa-mir-526a-1	0	0	0
hsa-mir-520c	0	0	0
hsa-mir-518c	0	0	0
hsa-mir-518c	0	0	0
hsa-mir-524	0	0	0
hsa-mir-517a	0	0	0
hsa-mir-519d	0	0	0
hsa-mir-521-2	0	0	0
hsa-mir-520d	0	0	0
hsa-mir-517b	0	0	0
hsa-mir-520g	0	0	0
hsa-mir-516b-2	0	0	0
hsa-mir-526a-2	0	0	0
hsa-mir-518e	0	0	0
hsa-mir-518a-1	0	0	0
hsa-mir-518d	0	0	0
hsa-mir-516b-1	0	0	0
hsa-mir-518a-2	0	0	0
hsa-mir-517c	0	0	0
hsa-mir-520h	0	0	0
hsa-mir-521-1	0	0	0
hsa-mir-522	0	0	0
hsa-mir-519a-1	0	0	0
hsa-mir-527	0	0	0
hsa-mir-516a-1	0	0	0
hsa-mir-516a-2	0	0	0
hsa-mir-519a-2	0	0	0
hsa-mir-499a	0	0	0
hsa-mir-500a	0	0	0
hsa-mir-501	0	0	0
hsa-mir-501	0	0	0
hsa-mir-502	0	0	0
hsa-mir-502	0	0	0
hsa-mir-450a-2	0	0	0
hsa-mir-450a-2	0	0	0
hsa-mir-503	0	0	0
hsa-mir-504	0	0	0
hsa-mir-505	0	0	0
hsa-mir-513a-1	0	0	0
hsa-mir-513a-2	0	0	0
hsa-mir-506	0	0	0
hsa-mir-507	0	0	0
hsa-mir-508	0	0	0
hsa-mir-509-1	0	0	0
hsa-mir-510	0	0	0

hsa-mir-514a-1	0	0	0
hsa-mir-514a-2	0	0	0
hsa-mir-514a-3	0	0	0
hsa-mir-532	0	0	0
hsa-mir-455	0	0	0
hsa-mir-539	0	0	0
hsa-mir-544a	0	0	0
hsa-mir-545	0	0	0
hsa-mir-376a-2	0	0	0
hsa-mir-487b	0	0	0
hsa-mir-487b	0	0	0
hsa-mir-551a	0	0	0
hsa-mir-552	0	0	0
hsa-mir-553	0	0	0
hsa-mir-554	0	0	0
hsa-mir-92b	0	0	0
hsa-mir-555	0	0	0
hsa-mir-556	0	0	0
hsa-mir-557	0	0	0
hsa-mir-558	0	0	0
hsa-mir-559	0	0	0
hsa-mir-561	0	0	0
hsa-mir-562	0	0	0
hsa-mir-563	0	0	0
hsa-mir-564	0	0	0
hsa-mir-567	0	0	0
hsa-mir-568	0	0	0
hsa-mir-551b	0	0	0
hsa-mir-569	0	0	0
hsa-mir-570	0	0	0
hsa-mir-570	0	0	0
hsa-mir-571	0	0	0
hsa-mir-572	0	0	0
hsa-mir-573	0	0	0
hsa-mir-574	0	0	0
hsa-mir-575	0	0	0
hsa-mir-576	0	0	0
hsa-mir-577	0	0	0
hsa-mir-578	0	0	0
hsa-mir-579	0	0	0
hsa-mir-580	0	0	0
hsa-mir-581	0	0	0
hsa-mir-582	0	0	0
hsa-mir-583	0	0	0
hsa-mir-584	0	0	0
hsa-mir-585	0	0	0
hsa-mir-548a-1	0	0	0
hsa-mir-586	0	0	0
hsa-mir-587	0	0	0
hsa-mir-548b	0	0	0
hsa-mir-588	0	0	0
hsa-mir-548a-2	0	0	0

hsa-mir-589	0	0	0
hsa-mir-550a-1	0	0	0
hsa-mir-550a-2	0	0	0
hsa-mir-590	0	0	0
hsa-mir-591	0	0	0
hsa-mir-592	0	0	0
hsa-mir-593	0	0	0
hsa-mir-595	0	0	0
hsa-mir-596	0	0	0
hsa-mir-597	0	0	0
hsa-mir-598	0	0	0
hsa-mir-599	0	0	0
hsa-mir-548a-3	0	0	0
hsa-mir-548a-3	0	0	0
hsa-mir-600	0	0	0
hsa-mir-601	0	0	0
hsa-mir-602	0	0	0
hsa-mir-603	0	0	0
hsa-mir-603	0	0	0
hsa-mir-604	0	0	0
hsa-mir-605	0	0	0
hsa-mir-606	0	0	0
hsa-mir-607	0	0	0
hsa-mir-608	0	0	0
hsa-mir-609	0	0	0
hsa-mir-610	0	0	0
hsa-mir-611	0	0	0
hsa-mir-612	0	0	0
hsa-mir-613	0	0	0
hsa-mir-614	0	0	0
hsa-mir-615	0	0	0
hsa-mir-616	0	0	0
hsa-mir-548c	0	0	0
hsa-mir-617	0	0	0
hsa-mir-618	0	0	0
hsa-mir-619	0	0	0
hsa-mir-620	0	0	0
hsa-mir-621	0	0	0
hsa-mir-622	0	0	0
hsa-mir-623	0	0	0
hsa-mir-624	0	0	0
hsa-mir-625	0	0	0
hsa-mir-626	0	0	0
hsa-mir-627	0	0	0
hsa-mir-628	0	0	0
hsa-mir-629	0	0	0
hsa-mir-630	0	0	0
hsa-mir-631	0	0	0
hsa-mir-33b	0	0	0
hsa-mir-632	0	0	0
hsa-mir-633	0	0	0
hsa-mir-634	0	0	0

hsa-mir-635	0	0	0
hsa-mir-636	0	0	0
hsa-mir-637	0	0	0
hsa-mir-638	0	0	0
hsa-mir-639	0	0	0
hsa-mir-640	0	0	0
hsa-mir-641	0	0	0
hsa-mir-642a	0	0	0
hsa-mir-642a	0	0	0
hsa-mir-643	0	0	0
hsa-mir-644a	0	0	0
hsa-mir-645	0	0	0
hsa-mir-646	0	0	0
hsa-mir-647	0	0	0
hsa-mir-648	0	0	0
hsa-mir-649	0	0	0
hsa-mir-650	0	0	0
hsa-mir-651	0	0	0
hsa-mir-652	0	0	0
hsa-mir-548d-1	0	0	0
hsa-mir-661	0	0	0
hsa-mir-662	0	0	0
hsa-mir-663a	0	0	0
hsa-mir-663a	0	0	0
hsa-mir-449b	0	0	0
hsa-mir-449b	0	0	0
hsa-mir-653	0	0	0
hsa-mir-411	0	0	0
hsa-mir-654	0	0	0
hsa-mir-655	0	0	0
hsa-mir-656	0	0	0
hsa-mir-549a	0	0	0
hsa-mir-657	0	0	0
hsa-mir-658	0	0	0
hsa-mir-659	0	0	0
hsa-mir-660	0	0	0
hsa-mir-421	0	0	0
hsa-mir-542	0	0	0
hsa-mir-758	0	0	0
hsa-mir-1264	0	0	0
hsa-mir-671	0	0	0
hsa-mir-668	0	0	0
hsa-mir-550a-3	0	0	0
hsa-mir-767	0	0	0
hsa-mir-1224	0	0	0
hsa-mir-151b	0	0	0
hsa-mir-320b-1	0	0	0
hsa-mir-320b-1	0	0	0
hsa-mir-320c-1	0	0	0
hsa-mir-1296	0	0	0
hsa-mir-1468	0	0	0
hsa-mir-1323	0	0	0

hsa-mir-1271	0	0	0
hsa-mir-1301	0	0	0
hsa-mir-454	0	0	0
hsa-mir-1185-2	0	0	0
hsa-mir-449c	0	0	0
hsa-mir-1283-1	0	0	0
hsa-mir-769	0	0	0
hsa-mir-766	0	0	0
hsa-mir-320b-2	0	0	0
hsa-mir-378d-2	0	0	0
hsa-mir-1185-1	0	0	0
hsa-mir-762	0	0	0
hsa-mir-802	0	0	0
hsa-mir-670	0	0	0
hsa-mir-1298	0	0	0
hsa-mir-2113	0	0	0
hsa-mir-761	0	0	0
hsa-mir-764	0	0	0
hsa-mir-759	0	0	0
hsa-mir-765	0	0	0
hsa-mir-770	0	0	0
hsa-mir-675	0	0	0
hsa-mir-298	0	0	0
hsa-mir-891a	0	0	0
hsa-mir-891a	0	0	0
hsa-mir-300	0	0	0
hsa-mir-892a	0	0	0
hsa-mir-892a	0	0	0
hsa-mir-509-2	0	0	0
hsa-mir-450b	0	0	0
hsa-mir-874	0	0	0
hsa-mir-890	0	0	0
hsa-mir-891b	0	0	0
hsa-mir-891b	0	0	0
hsa-mir-888	0	0	0
hsa-mir-892b	0	0	0
hsa-mir-541	0	0	0
hsa-mir-889	0	0	0
hsa-mir-875	0	0	0
hsa-mir-876	0	0	0
hsa-mir-708	0	0	0
hsa-mir-147b	0	0	0
hsa-mir-147b	0	0	0
hsa-mir-190b	0	0	0
hsa-mir-744	0	0	0
hsa-mir-885	0	0	0
hsa-mir-877	0	0	0
hsa-mir-887	0	0	0
hsa-mir-665	0	0	0
hsa-mir-873	0	0	0
hsa-mir-543	0	0	0
hsa-mir-374b	0	0	0

hsa-mir-760	0	0	0
hsa-mir-301b	0	0	0
hsa-mir-301b	0	0	0
hsa-mir-216b	0	0	0
hsa-mir-208b	0	0	0
hsa-mir-920	0	0	0
hsa-mir-921	0	0	0
hsa-mir-922	0	0	0
hsa-mir-924	0	0	0
hsa-mir-509-3	0	0	0
hsa-mir-933	0	0	0
hsa-mir-934	0	0	0
hsa-mir-935	0	0	0
hsa-mir-936	0	0	0
hsa-mir-937	0	0	0
hsa-mir-938	0	0	0
hsa-mir-939	0	0	0
hsa-mir-940	0	0	0
hsa-mir-941-1	0	0	0
hsa-mir-941-2	0	0	0
hsa-mir-941-3	0	0	0
hsa-mir-941-4	0	0	0
hsa-mir-942	0	0	0
hsa-mir-943	0	0	0
hsa-mir-944	0	0	0
hsa-mir-297	0	0	0
hsa-mir-1178	0	0	0
hsa-mir-1179	0	0	0
hsa-mir-1180	0	0	0
hsa-mir-1181	0	0	0
hsa-mir-1182	0	0	0
hsa-mir-1183	0	0	0
hsa-mir-1184-1	0	0	0
hsa-mir-1225	0	0	0
hsa-mir-1226	0	0	0
hsa-mir-1227	0	0	0
hsa-mir-1228	0	0	0
hsa-mir-1229	0	0	0
hsa-mir-1231	0	0	0
hsa-mir-1233-1	0	0	0
hsa-mir-1234	0	0	0
hsa-mir-1236	0	0	0
hsa-mir-1237	0	0	0
hsa-mir-1238	0	0	0
hsa-mir-1200	0	0	0
hsa-mir-1202	0	0	0
hsa-mir-1203	0	0	0
hsa-mir-663b	0	0	0
hsa-mir-663b	0	0	0
hsa-mir-1204	0	0	0
hsa-mir-1205	0	0	0
hsa-mir-1206	0	0	0

hsa-mir-1207	0	0	0
hsa-mir-1208	0	0	0
hsa-mir-548e	0	0	0
hsa-mir-548j	0	0	0
hsa-mir-548j	0	0	0
hsa-mir-1285-1	0	0	0
hsa-mir-1285-1	0	0	0
hsa-mir-1285-2	0	0	0
hsa-mir-1286	0	0	0
hsa-mir-1287	0	0	0
hsa-mir-1289-1	0	0	0
hsa-mir-1289-2	0	0	0
hsa-mir-1289-2	0	0	0
hsa-mir-1290	0	0	0
hsa-mir-1291	0	0	0
hsa-mir-548k	0	0	0
hsa-mir-548k	0	0	0
hsa-mir-1293	0	0	0
hsa-mir-1294	0	0	0
hsa-mir-1295a	0	0	0
hsa-mir-1297	0	0	0
hsa-mir-1299	0	0	0
hsa-mir-548l	0	0	0
hsa-mir-1302-1	0	0	0
hsa-mir-1302-2	0	0	0
hsa-mir-1302-3	0	0	0
hsa-mir-1302-4	0	0	0
hsa-mir-1302-5	0	0	0
hsa-mir-1302-6	0	0	0
hsa-mir-1302-7	0	0	0
hsa-mir-1302-8	0	0	0
hsa-mir-1304	0	0	0
hsa-mir-1305	0	0	0
hsa-mir-1243	0	0	0
hsa-mir-548f-1	0	0	0
hsa-mir-548f-1	0	0	0
hsa-mir-548f-2	0	0	0
hsa-mir-548f-3	0	0	0
hsa-mir-548f-3	0	0	0
hsa-mir-548f-4	0	0	0
hsa-mir-548f-4	0	0	0
hsa-mir-548f-5	0	0	0
hsa-mir-548f-5	0	0	0
hsa-mir-1244-1	0	0	0
hsa-mir-1245a	0	0	0
hsa-mir-1246	0	0	0
hsa-mir-1247	0	0	0
hsa-mir-1248	0	0	0
hsa-mir-1249	0	0	0
hsa-mir-1250	0	0	0
hsa-mir-1251	0	0	0
hsa-mir-1253	0	0	0

hsa-mir-1254-1	0	0	0
hsa-mir-1254-1	0	0	0
hsa-mir-1255a	0	0	0
hsa-mir-1256	0	0	0
hsa-mir-1257	0	0	0
hsa-mir-1258	0	0	0
hsa-mir-1260a	0	0	0
hsa-mir-548g	0	0	0
hsa-mir-548g	1	1	0
hsa-mir-1261	0	0	0
hsa-mir-1262	0	0	0
hsa-mir-1262	0	0	0
hsa-mir-1263	0	0	0
hsa-mir-548m	0	0	0
hsa-mir-548m	0	0	0
hsa-mir-1265	0	0	0
hsa-mir-548o	0	0	0
hsa-mir-1266	0	0	0
hsa-mir-1267	0	0	0
hsa-mir-1269a	0	0	0
hsa-mir-1270-1	0	0	0
hsa-mir-1272	0	0	0
hsa-mir-548h-1	0	0	0
hsa-mir-548h-1	0	0	0
hsa-mir-548h-2	0	0	0
hsa-mir-548h-3	0	0	0
hsa-mir-548h-3	0	0	0
hsa-mir-548h-4	0	0	0
hsa-mir-1275	0	0	0
hsa-mir-1276	0	0	0
hsa-mir-302e	0	0	0
hsa-mir-302f	0	0	0
hsa-mir-1277	0	0	0
hsa-mir-548p	0	0	0
hsa-mir-548p	0	0	0
hsa-mir-548i-1	0	0	0
hsa-mir-548i-2	0	0	0
hsa-mir-548i-2	0	0	0
hsa-mir-548i-3	0	0	0
hsa-mir-548i-4	0	0	0
hsa-mir-548i-4	0	0	0
hsa-mir-1278	0	0	0
hsa-mir-1279	0	0	0
hsa-mir-1281	0	0	0
hsa-mir-1282	0	0	0
hsa-mir-1283-2	0	0	0
hsa-mir-1284	0	0	0
hsa-mir-1288	0	0	0
hsa-mir-1292	0	0	0
hsa-mir-1252	0	0	0
hsa-mir-1255b-1	0	0	0
hsa-mir-1255b-2	0	0	0

hsa-mir-664a	0	0	0
hsa-mir-1306	0	0	0
hsa-mir-1307	0	0	0
hsa-mir-513b	0	0	0
hsa-mir-513b	0	0	0
hsa-mir-513c	0	0	0
hsa-mir-1321	0	0	0
hsa-mir-1322	0	0	0
hsa-mir-1197	0	0	0
hsa-mir-1324	0	0	0
hsa-mir-1471	0	0	0
hsa-mir-1537	0	0	0
hsa-mir-1538	0	0	0
hsa-mir-1539	0	0	0
hsa-mir-103b-1	0	0	0
hsa-mir-103b-1	0	0	0
hsa-mir-103b-2	0	0	0
hsa-mir-103b-2	0	0	0
hsa-mir-320d-1	0	0	0
hsa-mir-320c-2	0	0	0
hsa-mir-320c-2	0	0	0
hsa-mir-320d-2	0	0	0
hsa-mir-1825	0	0	0
hsa-mir-1827	0	0	0
hsa-mir-1908	0	0	0
hsa-mir-1909	0	0	0
hsa-mir-1910	0	0	0
hsa-mir-1911	0	0	0
hsa-mir-1912	0	0	0
hsa-mir-1913	0	0	0
hsa-mir-1914	0	0	0
hsa-mir-1915	0	0	0
hsa-mir-1972-1	0	0	0
hsa-mir-1973	0	0	0
hsa-mir-1976	0	0	0
hsa-mir-2052	0	0	0
hsa-mir-2053	0	0	0
hsa-mir-2110	0	0	0
hsa-mir-2114	0	0	0
hsa-mir-2115	0	0	0
hsa-mir-2116	0	0	0
hsa-mir-2117	0	0	0
hsa-mir-548q	0	0	0
hsa-mir-2276	0	0	0
hsa-mir-2277	0	0	0
hsa-mir-2278	0	0	0
hsa-mir-2681	0	0	0
hsa-mir-2682	0	0	0
hsa-mir-711	0	0	0
hsa-mir-2861	0	0	0
hsa-mir-2909	0	0	0
hsa-mir-3115	0	0	0

hsa-mir-3116-1	0	0	0
hsa-mir-3116-1	0	0	0
hsa-mir-3116-1	0	0	0
hsa-mir-3116-2	0	0	0
hsa-mir-3116-2	0	0	0
hsa-mir-3116-2	0	0	0
hsa-mir-3117	0	0	0
hsa-mir-3118-1	0	0	0
hsa-mir-3118-2	0	0	0
hsa-mir-3118-3	0	0	0
hsa-mir-3119-1	0	0	0
hsa-mir-3119-1	0	0	0
hsa-mir-3119-2	0	0	0
hsa-mir-3119-2	0	0	0
hsa-mir-3120	0	0	0
hsa-mir-3121	0	0	0
hsa-mir-3122	0	0	0
hsa-mir-3123	0	0	0
hsa-mir-3124	0	0	0
hsa-mir-548s	0	0	0
hsa-mir-548s	0	0	0
hsa-mir-3125	0	0	0
hsa-mir-3126	0	0	0
hsa-mir-3127	0	0	0
hsa-mir-3128	0	0	0
hsa-mir-3129	0	0	0
hsa-mir-3130-1	0	0	0
hsa-mir-3130-1	0	0	0
hsa-mir-3130-1	0	0	0
hsa-mir-3130-2	0	0	0
hsa-mir-3130-2	0	0	0
hsa-mir-3130-2	0	0	0
hsa-mir-3131	0	0	0
hsa-mir-3132	0	0	0
hsa-mir-378b	0	0	0
hsa-mir-3134	0	0	0
hsa-mir-3135a	0	0	0
hsa-mir-3135a	0	0	0
hsa-mir-466	0	0	0
hsa-mir-3136	0	0	0
hsa-mir-544b	0	0	0
hsa-mir-3137	0	0	0
hsa-mir-3138	0	0	0
hsa-mir-3139	0	0	0
hsa-mir-3140	0	0	0
hsa-mir-548t	0	0	0
hsa-mir-548t	0	0	0
hsa-mir-3141	0	0	0
hsa-mir-3142	0	0	0
hsa-mir-3142	0	0	0
hsa-mir-3143	0	0	0
hsa-mir-548u	0	0	0

hsa-mir-3144	0	0	0
hsa-mir-3145	0	0	0
hsa-mir-3146	0	0	0
hsa-mir-3147	0	0	0
hsa-mir-548v	0	0	0
hsa-mir-3148	0	0	0
hsa-mir-3149	0	0	0
hsa-mir-3150a	0	0	0
hsa-mir-3150a	0	0	0
hsa-mir-3151	0	0	0
hsa-mir-3152	0	0	0
hsa-mir-3153	0	0	0
hsa-mir-3074	0	0	0
hsa-mir-3154	0	0	0
hsa-mir-3155a	0	0	0
hsa-mir-3155a	0	0	0
hsa-mir-3156-1	0	0	0
hsa-mir-3157	0	0	0
hsa-mir-3158-1	0	0	0
hsa-mir-3158-1	0	0	0
hsa-mir-3158-1	0	0	0
hsa-mir-3158-2	0	0	0
hsa-mir-3158-2	0	0	0
hsa-mir-3158-2	0	0	0
hsa-mir-3159	0	0	0
hsa-mir-3160-1	0	0	0
hsa-mir-3160-1	0	0	0
hsa-mir-3160-1	0	0	0
hsa-mir-3160-2	0	0	0
hsa-mir-3160-2	0	0	0
hsa-mir-3160-2	0	0	0
hsa-mir-3161	0	0	0
hsa-mir-3162	0	0	0
hsa-mir-3163	0	0	0
hsa-mir-3164	0	0	0
hsa-mir-3165	0	0	0
hsa-mir-3166	0	0	0
hsa-mir-1260b	0	0	0
hsa-mir-3167	0	0	0
hsa-mir-3168	0	0	0
hsa-mir-3169	0	0	0
hsa-mir-3170	0	0	0
hsa-mir-3171	0	0	0
hsa-mir-3173	0	0	0
hsa-mir-1193	0	0	0
hsa-mir-323b	0	0	0
hsa-mir-323b	0	0	0
hsa-mir-3118-4	0	0	0
hsa-mir-3174	0	0	0
hsa-mir-3175	0	0	0
hsa-mir-3176	0	0	0
hsa-mir-3177	0	0	0

hsa-mir-3178	0	0	0
hsa-mir-3179-1	0	0	0
hsa-mir-3180-1	0	0	0
hsa-mir-3180-2	0	0	0
hsa-mir-3179-2	0	0	0
hsa-mir-3180-3	0	0	0
hsa-mir-3179-3	0	0	0
hsa-mir-548w	0	0	0
hsa-mir-3181	0	0	0
hsa-mir-3182	0	0	0
hsa-mir-3183	0	0	0
hsa-mir-3184	0	0	0
hsa-mir-3185	0	0	0
hsa-mir-3065	0	0	0
hsa-mir-3186	0	0	0
hsa-mir-3156-2	0	0	0
hsa-mir-3187	0	0	0
hsa-mir-3188	0	0	0
hsa-mir-3189	0	0	0
hsa-mir-320e	0	0	0
hsa-mir-3190	0	0	0
hsa-mir-3191	0	0	0
hsa-mir-3192	0	0	0
hsa-mir-3193	0	0	0
hsa-mir-3194	0	0	0
hsa-mir-3156-3	0	0	0
hsa-mir-3118-5	0	0	0
hsa-mir-548x	0	0	0
hsa-mir-3197	0	0	0
hsa-mir-3198-1	0	0	0
hsa-mir-3199-1	0	0	0
hsa-mir-3199-1	0	0	0
hsa-mir-3199-1	0	0	0
hsa-mir-3199-2	0	0	0
hsa-mir-3199-2	0	0	0
hsa-mir-3199-2	0	0	0
hsa-mir-3200	0	0	0
hsa-mir-3201	0	0	0
hsa-mir-3201	0	0	0
hsa-mir-514b	0	0	0
hsa-mir-3202-1	0	0	0
hsa-mir-3202-1	0	0	0
hsa-mir-3202-1	0	0	0
hsa-mir-3202-2	0	0	0
hsa-mir-3202-2	0	0	0
hsa-mir-3202-2	0	0	0
hsa-mir-4295	0	0	0
hsa-mir-4296	0	0	0
hsa-mir-4297	0	0	0
hsa-mir-378c	0	0	0
hsa-mir-4293	0	0	0
hsa-mir-4294	0	0	0

hsa-mir-4301	0	0	0
hsa-mir-4299	0	0	0
hsa-mir-4298	0	0	0
hsa-mir-4300	0	0	0
hsa-mir-4304	0	0	0
hsa-mir-4302	0	0	0
hsa-mir-4303	0	0	0
hsa-mir-4305	0	0	0
hsa-mir-4306	0	0	0
hsa-mir-4309	0	0	0
hsa-mir-4307	0	0	0
hsa-mir-4308	0	0	0
hsa-mir-4310	0	0	0
hsa-mir-4311	0	0	0
hsa-mir-4312	0	0	0
hsa-mir-4313	0	0	0
hsa-mir-4315-1	0	0	0
hsa-mir-4316	0	0	0
hsa-mir-4314	0	0	0
hsa-mir-4318	0	0	0
hsa-mir-4319	0	0	0
hsa-mir-4320	0	0	0
hsa-mir-4317	0	0	0
hsa-mir-4322	0	0	0
hsa-mir-4321	0	0	0
hsa-mir-4323	0	0	0
hsa-mir-4324	0	0	0
hsa-mir-4256	0	0	0
hsa-mir-4257	0	1	1
hsa-mir-4258	0	0	0
hsa-mir-4259	0	0	0
hsa-mir-4260	0	0	0
hsa-mir-4253	0	0	0
hsa-mir-4251	0	0	0
hsa-mir-4254	0	0	0
hsa-mir-4255	0	0	0
hsa-mir-4252	0	0	0
hsa-mir-4325	0	0	0
hsa-mir-4326	0	0	0
hsa-mir-4327	0	0	0
hsa-mir-4261	0	0	0
hsa-mir-4265	0	0	0
hsa-mir-4266	0	0	0
hsa-mir-4267	0	0	0
hsa-mir-4262	0	0	0
hsa-mir-2355	0	0	0
hsa-mir-4268	0	0	0
hsa-mir-4269	0	0	0
hsa-mir-4263	0	0	0
hsa-mir-4264	0	0	0
hsa-mir-4270	0	0	0
hsa-mir-4271	0	0	0

hsa-mir-4272	0	0	0
hsa-mir-4273	0	0	0
hsa-mir-4276	0	0	0
hsa-mir-4275	0	0	0
hsa-mir-4274	0	0	0
hsa-mir-4281	0	0	0
hsa-mir-4277	0	0	0
hsa-mir-4279	0	0	0
hsa-mir-4278	0	0	0
hsa-mir-4280	0	0	0
hsa-mir-4282	0	0	0
hsa-mir-4285	0	0	0
hsa-mir-4283-1	0	0	0
hsa-mir-4284	0	0	0
hsa-mir-4286	0	0	0
hsa-mir-4287	0	0	0
hsa-mir-4288	0	0	0
hsa-mir-4292	0	0	0
hsa-mir-4289	0	0	0
hsa-mir-4290	0	0	0
hsa-mir-4291	0	0	0
hsa-mir-4329	0	0	0
hsa-mir-4330	0	0	0
hsa-mir-500b	0	0	0
hsa-mir-500b	0	0	0
hsa-mir-4328	0	0	0
hsa-mir-1184-2	0	0	0
hsa-mir-1184-3	0	0	0
hsa-mir-1233-2	0	0	0
hsa-mir-1244-2	0	0	0
hsa-mir-1244-3	0	0	0
hsa-mir-1270-2	0	0	0
hsa-mir-1972-2	0	0	0
hsa-mir-1302-9	0	0	0
hsa-mir-1302-10	0	0	0
hsa-mir-1302-11	0	0	0
hsa-mir-3118-6	0	0	0
hsa-mir-4283-2	0	0	0
hsa-mir-4315-2	0	0	0
hsa-mir-3605	0	0	0
hsa-mir-3606	0	0	0
hsa-mir-3607	0	0	0
hsa-mir-3609	0	0	0
hsa-mir-3610	0	0	0
hsa-mir-3611	0	0	0
hsa-mir-3612	0	0	0
hsa-mir-3614	0	0	0
hsa-mir-3615	0	0	0
hsa-mir-3616	0	0	0
hsa-mir-3617	0	0	0
hsa-mir-3618	0	0	0
hsa-mir-3619	0	0	0

hsa-mir-23c	0	0	0
hsa-mir-3620	0	0	0
hsa-mir-3621	0	0	0
hsa-mir-3622a	0	0	0
hsa-mir-3622b	0	0	0
hsa-mir-3622b	0	0	0
hsa-mir-3646	0	0	0
hsa-mir-3648	0	0	0
hsa-mir-3649	0	0	0
hsa-mir-3650	0	0	0
hsa-mir-3651	0	0	0
hsa-mir-3652	0	0	0
hsa-mir-3653	0	0	0
hsa-mir-3654	0	0	0
hsa-mir-3655	0	0	0
hsa-mir-3656	0	0	1
hsa-mir-3657	0	0	0
hsa-mir-3658	0	0	0
hsa-mir-3659	0	0	0
hsa-mir-3660	0	0	0
hsa-mir-3661	0	0	0
hsa-mir-3662	0	0	0
hsa-mir-3663	0	0	0
hsa-mir-3664	0	0	0
hsa-mir-3665	0	0	0
hsa-mir-3666	0	0	0
hsa-mir-3667	0	0	0
hsa-mir-3668	0	0	0
hsa-mir-3669	0	0	0
hsa-mir-3670-1	0	0	0
hsa-mir-3671	0	0	0
hsa-mir-3672	0	0	0
hsa-mir-3673	0	0	0
hsa-mir-3674	0	0	0
hsa-mir-3675	0	0	0
hsa-mir-3677	0	0	0
hsa-mir-3678	0	0	0
hsa-mir-3679	0	0	0
hsa-mir-3680-1	0	0	0
hsa-mir-3681	0	0	0
hsa-mir-3682	0	0	0
hsa-mir-3683	0	0	0
hsa-mir-3684	0	0	0
hsa-mir-3685	0	0	0
hsa-mir-3686	0	0	0
hsa-mir-3687	0	0	0
hsa-mir-3688-1	0	0	0
hsa-mir-3688-1	0	0	0
hsa-mir-3689a	0	0	0
hsa-mir-3690-1	0	0	0
hsa-mir-3691	0	0	0
hsa-mir-3692	0	0	0

hsa-mir-3713	0	0	0
hsa-mir-3714	0	0	0
hsa-mir-3180-4	0	0	0
hsa-mir-3180-5	0	0	0
hsa-mir-3907	0	0	0
hsa-mir-3689b	0	0	0
hsa-mir-3689b	0	0	0
hsa-mir-3908	0	0	0
hsa-mir-3909	0	0	0
hsa-mir-3910-1	0	0	0
hsa-mir-3910-1	0	0	0
hsa-mir-3910-1	0	0	0
hsa-mir-3911	0	0	0
hsa-mir-3912	0	0	0
hsa-mir-3913-1	0	0	0
hsa-mir-3913-2	0	0	0
hsa-mir-3914-1	0	0	0
hsa-mir-3915	0	0	0
hsa-mir-3914-2	0	0	0
hsa-mir-3914-2	0	0	0
hsa-mir-3916	0	0	0
hsa-mir-3917	0	0	0
hsa-mir-3918	0	0	0
hsa-mir-3919	0	0	0
hsa-mir-3150b	0	0	0
hsa-mir-3150b	0	0	0
hsa-mir-3920	0	0	0
hsa-mir-3921	0	0	0
hsa-mir-3922	0	0	0
hsa-mir-3923	0	0	0
hsa-mir-3910-2	0	0	0
hsa-mir-3910-2	0	0	0
hsa-mir-3910-2	0	0	0
hsa-mir-3924	0	0	0
hsa-mir-3925	0	0	0
hsa-mir-3926-1	0	0	0
hsa-mir-3926-1	0	0	0
hsa-mir-3927	0	0	0
hsa-mir-676	0	0	0
hsa-mir-3926-2	0	0	0
hsa-mir-3926-2	0	0	0
hsa-mir-3928	0	0	0
hsa-mir-3934	0	0	0
hsa-mir-3935	0	0	0
hsa-mir-3936	0	0	0
hsa-mir-3937	0	0	0
hsa-mir-3938	0	0	0
hsa-mir-548y	0	0	0
hsa-mir-3939	0	0	0
hsa-mir-3940	0	0	0
hsa-mir-3941	0	0	0
hsa-mir-3942	0	0	0

hsa-mir-3943	0	0	0
hsa-mir-3944	0	0	0
hsa-mir-3945	0	0	0
hsa-mir-374c	0	0	0
hsa-mir-642b	0	0	0
hsa-mir-550b-1	0	0	0
hsa-mir-550b-2	0	0	0
hsa-mir-548z	0	0	0
hsa-mir-548z	0	0	0
hsa-mir-548aa-1	0	0	0
hsa-mir-548o-2	0	0	0
hsa-mir-1254-2	0	0	0
hsa-mir-378d-1	0	0	0
hsa-mir-378e	0	0	0
hsa-mir-548ab	0	0	0
hsa-mir-548ab	0	0	0
hsa-mir-4417	0	0	0
hsa-mir-4418	0	0	0
hsa-mir-4419a	0	0	0
hsa-mir-378f	0	0	0
hsa-mir-4420	0	0	0
hsa-mir-4421	0	0	0
hsa-mir-4422	0	0	0
hsa-mir-4423	0	0	0
hsa-mir-378g	0	0	0
hsa-mir-4424	0	0	0
hsa-mir-4425	0	0	0
hsa-mir-4426	0	0	0
hsa-mir-4427	0	0	0
hsa-mir-4428	0	0	0
hsa-mir-4429	0	0	0
hsa-mir-4430	0	0	0
hsa-mir-548ad	0	0	0
hsa-mir-4431	0	0	0
hsa-mir-4432	0	0	0
hsa-mir-4433	0	0	0
hsa-mir-4435-1	0	0	0
hsa-mir-4436a	0	0	0
hsa-mir-4435-2	0	0	0
hsa-mir-4437	0	0	0
hsa-mir-548ae-1	0	0	0
hsa-mir-548ae-2	0	0	0
hsa-mir-4438	0	0	0
hsa-mir-4439	0	0	0
hsa-mir-4440	0	0	0
hsa-mir-4440	0	0	0
hsa-mir-4441	0	0	0
hsa-mir-4442	0	0	0
hsa-mir-4443	0	0	0
hsa-mir-4444-1	0	0	0
hsa-mir-4445	0	0	0
hsa-mir-4446	0	0	0

hsa-mir-4447	0	0	0
hsa-mir-4449	0	0	0
hsa-mir-548ag-1	0	0	0
hsa-mir-548ag-2	0	0	0
hsa-mir-4450	0	0	0
hsa-mir-548ah	0	0	0
hsa-mir-548ah	0	0	0
hsa-mir-4451	0	0	0
hsa-mir-4452	0	0	0
hsa-mir-4453	0	0	0
hsa-mir-4454	0	0	0
hsa-mir-4455	0	0	0
hsa-mir-4456	0	0	0
hsa-mir-4457	0	0	0
hsa-mir-4458	0	0	0
hsa-mir-4459	0	0	0
hsa-mir-4460	0	0	0
hsa-mir-4461	0	0	0
hsa-mir-378h	0	0	0
hsa-mir-3135b	0	0	0
hsa-mir-4462	0	0	0
hsa-mir-4463	0	0	0
hsa-mir-4464	0	0	0
hsa-mir-548ai	0	0	0
hsa-mir-548aj-1	0	0	0
hsa-mir-4465	0	0	0
hsa-mir-4467	0	0	0
hsa-mir-4468	0	0	0
hsa-mir-4469	0	0	0
hsa-mir-4470	0	0	0
hsa-mir-4471	0	0	0
hsa-mir-4472-1	0	0	0
hsa-mir-4473	0	0	0
hsa-mir-4474	0	0	0
hsa-mir-4475	0	0	0
hsa-mir-4476	0	0	0
hsa-mir-4477a	0	0	0
hsa-mir-4477a	0	0	0
hsa-mir-4477b	0	0	0
hsa-mir-4477b	0	0	0
hsa-mir-4478	0	0	0
hsa-mir-3689c	0	0	0
hsa-mir-548x-2	0	0	0
hsa-mir-3689d-1	0	0	0
hsa-mir-3689d-2	0	0	0
hsa-mir-3689e	0	1	1
hsa-mir-3689f	0	0	0
hsa-mir-4479	0	0	0
hsa-mir-3155b	0	0	0
hsa-mir-3155b	0	0	0
hsa-mir-548ak	0	0	0
hsa-mir-4480	0	0	0

hsa-mir-4481	0	0	0
hsa-mir-4482	0	0	0
hsa-mir-4483	0	0	0
hsa-mir-4484	0	0	0
hsa-mir-4485	0	0	0
hsa-mir-4486	0	0	0
hsa-mir-4487	0	0	0
hsa-mir-4487	0	0	0
hsa-mir-4488	0	0	0
hsa-mir-4489	0	0	0
hsa-mir-548al	0	0	0
hsa-mir-4490	0	0	0
hsa-mir-4491	0	0	0
hsa-mir-4492	0	0	0
hsa-mir-4493	0	0	0
hsa-mir-4494	0	0	0
hsa-mir-4495	0	0	0
hsa-mir-4496	0	0	0
hsa-mir-4497	0	0	0
hsa-mir-4498	0	0	0
hsa-mir-4419b	0	0	0
hsa-mir-4499	0	0	0
hsa-mir-4500	0	0	0
hsa-mir-4501	0	0	0
hsa-mir-4502	0	0	0
hsa-mir-4503	0	0	0
hsa-mir-4504	0	0	0
hsa-mir-4505	0	0	0
hsa-mir-4506	0	0	0
hsa-mir-2392	0	0	0
hsa-mir-4507	0	0	0
hsa-mir-4508	0	0	0
hsa-mir-4509-1	0	0	0
hsa-mir-4509-2	0	0	0
hsa-mir-4509-3	0	0	0
hsa-mir-4510	0	0	0
hsa-mir-4511	0	0	0
hsa-mir-4512	0	0	0
hsa-mir-4513	0	0	0
hsa-mir-4514	0	0	0
hsa-mir-4515	0	0	0
hsa-mir-4516	0	0	0
hsa-mir-4517	0	0	0
hsa-mir-4518	0	0	0
hsa-mir-4519	0	0	0
hsa-mir-4520a	0	0	0
hsa-mir-4520a	0	0	0
hsa-mir-4521	0	0	0
hsa-mir-1269b	0	0	0
hsa-mir-4522	0	0	0
hsa-mir-4523	0	0	0
hsa-mir-4524a	0	0	0

hsa-mir-4525	0	0	0
hsa-mir-4526	0	0	0
hsa-mir-4527	0	0	0
hsa-mir-4528	0	0	0
hsa-mir-4529	0	0	0
hsa-mir-4530	0	0	0
hsa-mir-4531	0	0	0
hsa-mir-4532	0	0	0
hsa-mir-4533	0	0	0
hsa-mir-4534	0	0	0
hsa-mir-378i	0	0	0
hsa-mir-4535	0	0	0
hsa-mir-1587	0	0	0
hsa-mir-4536-1	0	0	0
hsa-mir-4536-1	0	0	0
hsa-mir-548an	0	0	0
hsa-mir-4537	0	0	0
hsa-mir-4538	0	0	0
hsa-mir-4539	0	0	0
hsa-mir-4540	0	0	0
hsa-mir-3960	0	0	0
hsa-mir-3972	0	0	0
hsa-mir-3973	0	0	0
hsa-mir-3974	0	0	0
hsa-mir-3975	0	0	0
hsa-mir-3976	0	0	0
hsa-mir-3977	0	0	0
hsa-mir-3978	0	0	0
hsa-mir-4632	0	0	0
hsa-mir-4633	0	0	0
hsa-mir-4634	0	0	0
hsa-mir-4635	0	0	0
hsa-mir-4636	0	0	0
hsa-mir-4637	0	0	0
hsa-mir-4638	0	0	0
hsa-mir-4639	0	0	0
hsa-mir-4640	0	0	0
hsa-mir-4641	0	0	0
hsa-mir-4642	0	0	0
hsa-mir-4643	0	0	0
hsa-mir-4644	0	0	0
hsa-mir-4645	0	0	0
hsa-mir-4646	0	0	0
hsa-mir-4647	0	0	0
hsa-mir-4648	0	0	0
hsa-mir-4649	0	0	0
hsa-mir-4650-1	0	0	0
hsa-mir-4650-2	0	0	0
hsa-mir-4651	0	0	0
hsa-mir-4652	0	0	0
hsa-mir-4653	0	0	0
hsa-mir-4654	0	0	0

hsa-mir-4655	0	0	0
hsa-mir-4656	0	0	0
hsa-mir-4657	0	0	0
hsa-mir-4658	0	0	0
hsa-mir-4659a	0	0	0
hsa-mir-4659a	0	0	0
hsa-mir-4660	0	0	0
hsa-mir-4661	0	0	0
hsa-mir-4662a	0	0	0
hsa-mir-4659b	0	0	0
hsa-mir-4663	0	0	0
hsa-mir-4662b	0	0	0
hsa-mir-4662b	0	0	0
hsa-mir-4664	0	0	0
hsa-mir-4665	0	0	0
hsa-mir-4666a	0	0	0
hsa-mir-4667	0	0	0
hsa-mir-4668	0	0	0
hsa-mir-219b	0	0	0
hsa-mir-4669	0	0	0
hsa-mir-4670	0	0	0
hsa-mir-4671	0	0	0
hsa-mir-4672	0	0	0
hsa-mir-4673	0	0	0
hsa-mir-4674	0	0	0
hsa-mir-4675	0	0	0
hsa-mir-4676	0	0	0
hsa-mir-4677	0	0	0
hsa-mir-4678	0	0	0
hsa-mir-4679-1	0	0	0
hsa-mir-4679-1	0	0	0
hsa-mir-4679-1	0	0	0
hsa-mir-4679-2	0	0	0
hsa-mir-4679-2	0	0	0
hsa-mir-4680	0	0	0
hsa-mir-4681	0	0	0
hsa-mir-4682	0	0	0
hsa-mir-4683	0	0	0
hsa-mir-4684	0	0	0
hsa-mir-4685	0	0	0
hsa-mir-4686	0	0	0
hsa-mir-4687	0	0	0
hsa-mir-1343	0	0	0
hsa-mir-4688	0	0	0
hsa-mir-4689	0	0	0
hsa-mir-4690	0	0	0
hsa-mir-4691	0	0	0
hsa-mir-4692	0	0	0
hsa-mir-4693	0	0	0
hsa-mir-4694	0	0	0
hsa-mir-4695	0	0	0
hsa-mir-4696	0	0	0

hsa-mir-4697	0	0	0
hsa-mir-4698	0	0	0
hsa-mir-4699	0	0	0
hsa-mir-4700	0	0	0
hsa-mir-4701	0	0	0
hsa-mir-3198-2	0	0	0
hsa-mir-4703	0	0	0
hsa-mir-4704	0	0	0
hsa-mir-4705	0	0	0
hsa-mir-4706	0	0	0
hsa-mir-4707	0	0	0
hsa-mir-4708	0	0	0
hsa-mir-4709	0	0	0
hsa-mir-203b	0	0	0
hsa-mir-4710	0	0	0
hsa-mir-4711	0	0	0
hsa-mir-4712	0	0	0
hsa-mir-4713	0	0	0
hsa-mir-4714	0	0	0
hsa-mir-4715	0	0	0
hsa-mir-4716	0	0	0
hsa-mir-4716	0	0	0
hsa-mir-3529	0	0	0
hsa-mir-4717	0	0	0
hsa-mir-4718	1	0	0
hsa-mir-4719	0	0	0
hsa-mir-4720	0	0	0
hsa-mir-4721	0	0	0
hsa-mir-4722	0	0	0
hsa-mir-4520b	0	0	0
hsa-mir-4723	0	0	0
hsa-mir-451b	0	0	0
hsa-mir-4724	0	0	0
hsa-mir-4725	0	0	0
hsa-mir-4726	0	0	0
hsa-mir-4727	0	0	0
hsa-mir-4728	0	0	0
hsa-mir-4729	0	0	0
hsa-mir-4730	0	0	0
hsa-mir-4731	0	0	0
hsa-mir-4732	0	0	0
hsa-mir-4733	0	0	0
hsa-mir-4734	0	0	0
hsa-mir-4735	0	0	0
hsa-mir-4736	0	0	0
hsa-mir-4737	0	0	0
hsa-mir-4737	0	0	0
hsa-mir-3064	0	0	0
hsa-mir-4738	0	0	0
hsa-mir-4739	0	0	0
hsa-mir-4740	0	0	0
hsa-mir-4741	0	0	0

hsa-mir-4742	0	0	0
hsa-mir-4743	0	0	0
hsa-mir-4744	0	0	0
hsa-mir-3591	0	0	0
hsa-mir-4745	0	0	0
hsa-mir-4746	0	0	0
hsa-mir-4747	0	0	0
hsa-mir-4748	0	0	0
hsa-mir-4749	0	0	0
hsa-mir-4750	0	0	0
hsa-mir-4751	0	0	0
hsa-mir-4752	0	0	0
hsa-mir-4753	0	0	0
hsa-mir-371b	0	0	0
hsa-mir-4754	0	0	0
hsa-mir-4755	0	0	0
hsa-mir-499b	0	0	0
hsa-mir-4756	0	0	0
hsa-mir-4757	0	0	0
hsa-mir-4758	0	0	0
hsa-mir-4758	0	0	0
hsa-mir-4759	0	0	0
hsa-mir-4760	0	0	0
hsa-mir-4761	0	0	0
hsa-mir-4762	0	0	0
hsa-mir-4763	0	0	0
hsa-mir-4764	0	0	0
hsa-mir-4765	0	0	0
hsa-mir-4766	0	0	0
hsa-mir-4767	0	0	0
hsa-mir-4768	0	0	0
hsa-mir-4769	0	0	0
hsa-mir-4770	0	0	0
hsa-mir-4771-1	0	0	0
hsa-mir-4771-2	0	0	0
hsa-mir-4772	0	0	0
hsa-mir-4773-1	0	0	0
hsa-mir-4773-1	0	0	0
hsa-mir-4773-1	0	0	0
hsa-mir-4773-2	0	0	0
hsa-mir-4773-2	0	0	0
hsa-mir-4773-2	0	0	0
hsa-mir-4774	0	0	0
hsa-mir-4775	0	0	0
hsa-mir-4776-1	0	0	0
hsa-mir-4776-1	0	0	0
hsa-mir-4776-1	0	0	0
hsa-mir-4776-2	0	0	0
hsa-mir-4776-2	0	0	0
hsa-mir-4776-2	0	0	0
hsa-mir-4777	0	0	0
hsa-mir-4778	0	0	0

hsa-mir-4779	0	0	0
hsa-mir-4780	0	0	0
hsa-mir-4436b-1	0	0	0
hsa-mir-4781	0	0	0
hsa-mir-4782	0	0	0
hsa-mir-4783	0	0	0
hsa-mir-4784	0	0	0
hsa-mir-4785	0	0	0
hsa-mir-1245b	0	0	0
hsa-mir-2467	0	0	0
hsa-mir-4786	0	0	0
hsa-mir-4787	0	0	0
hsa-mir-4788	0	0	0
hsa-mir-4789	0	0	0
hsa-mir-4790	0	0	0
hsa-mir-4791	0	0	0
hsa-mir-4792	0	0	0
hsa-mir-4793	0	0	0
hsa-mir-4794	0	0	0
hsa-mir-4795	0	0	0
hsa-mir-4796	0	0	0
hsa-mir-4797	0	0	0
hsa-mir-4798	0	0	0
hsa-mir-4799	0	0	0
hsa-mir-3688-2	0	0	0
hsa-mir-3688-2	0	0	0
hsa-mir-4800	0	0	0
hsa-mir-4801	0	0	0
hsa-mir-4802	0	0	0
hsa-mir-4803	0	0	0
hsa-mir-4804	0	0	0
hsa-mir-4999	0	0	0
hsa-mir-5000	0	0	0
hsa-mir-5001	0	0	0
hsa-mir-5002	0	0	0
hsa-mir-5003	0	0	0
hsa-mir-5004	0	0	0
hsa-mir-548ao	0	0	0
hsa-mir-5006	0	0	0
hsa-mir-5007	0	0	0
hsa-mir-548ap	0	0	0
hsa-mir-5008	0	0	0
hsa-mir-5009	0	0	0
hsa-mir-5010	0	0	0
hsa-mir-5011	0	0	0
hsa-mir-5047	0	0	0
hsa-mir-5087	0	0	0
hsa-mir-5088	0	0	0
hsa-mir-5089	0	0	0
hsa-mir-5090	0	0	0
hsa-mir-5091	0	0	0
hsa-mir-5092	0	0	0

hsa-mir-5093	0	0	0
hsa-mir-5094	0	0	0
hsa-mir-5095	0	0	0
hsa-mir-1273f	0	0	0
hsa-mir-1273g	0	0	0
hsa-mir-5186	0	0	0
hsa-mir-5187	0	0	0
hsa-mir-5188	0	0	0
hsa-mir-5189	0	0	0
hsa-mir-5190	0	0	0
hsa-mir-5191	0	0	0
hsa-mir-5192	0	0	0
hsa-mir-5193	0	0	0
hsa-mir-5194	0	0	0
hsa-mir-5195	0	0	0
hsa-mir-5196	0	0	0
hsa-mir-5197	0	0	0
hsa-mir-4436b-2	0	0	0
hsa-mir-4444-2	0	0	0
hsa-mir-3670-2	0	0	0
hsa-mir-3680-2	0	0	0
hsa-mir-4524b	0	0	0
hsa-mir-5571	0	0	0
hsa-mir-5100	0	0	0
hsa-mir-5572	0	0	0
hsa-mir-548aq	0	0	0
hsa-mir-548ar	0	0	0
hsa-mir-5579	0	0	0
hsa-mir-664b	0	0	0
hsa-mir-5580	0	0	0
hsa-mir-5581	0	0	0
hsa-mir-548at	0	0	0
hsa-mir-5582	0	0	0
hsa-mir-5583-1	0	0	0
hsa-mir-5583-2	0	0	0
hsa-mir-5583-2	0	0	0
hsa-mir-5584	0	0	0
hsa-mir-5585	0	0	0
hsa-mir-5586	0	0	0
hsa-mir-5587	0	0	0
hsa-mir-548au	0	0	0
hsa-mir-1295b	0	0	0
hsa-mir-5589	0	0	0
hsa-mir-4536-2	0	0	0
hsa-mir-5590	0	0	0
hsa-mir-5591	0	0	0
hsa-mir-548av	0	0	0
hsa-mir-548av	0	0	0
hsa-mir-5680	0	0	0
hsa-mir-5681a	0	0	0
hsa-mir-5681a	0	0	0
hsa-mir-5682	0	0	0

hsa-mir-548aw	0	0	0
hsa-mir-5683	0	0	0
hsa-mir-5684	0	0	0
hsa-mir-5685	0	0	0
hsa-mir-5692c-1	0	0	0
hsa-mir-5692c-1	0	0	0
hsa-mir-5692c-2	0	0	0
hsa-mir-5687	0	0	0
hsa-mir-5688	0	0	0
hsa-mir-5681b	0	0	0
hsa-mir-5681b	0	0	0
hsa-mir-5689	0	0	0
hsa-mir-5690	0	0	0
hsa-mir-5691	0	0	0
hsa-mir-5692a-1	0	0	0
hsa-mir-5692a-2	0	0	0
hsa-mir-4666b	0	0	0
hsa-mir-5693	0	0	0
hsa-mir-5694	0	0	0
hsa-mir-5695	0	0	0
hsa-mir-5696	0	0	0
hsa-mir-5697	0	0	0
hsa-mir-5698	0	0	0
hsa-mir-5699	0	0	0
hsa-mir-5700	0	0	0
hsa-mir-5701-1	0	0	0
hsa-mir-5702	0	0	0
hsa-mir-5703	0	0	0
hsa-mir-5692b	0	0	0
hsa-mir-5704	0	0	0
hsa-mir-5705	0	0	0
hsa-mir-5706	0	0	0
hsa-mir-5707	0	0	0
hsa-mir-5708	0	0	0
hsa-mir-5739	0	0	0
hsa-mir-5701-2	0	0	0
hsa-mir-5787	0	0	0
hsa-mir-1199	0	0	0
hsa-mir-6068	0	0	0
hsa-mir-6069	0	0	0
hsa-mir-6070	0	0	0
hsa-mir-6071	0	0	0
hsa-mir-6072	0	0	0
hsa-mir-6073	0	0	0
hsa-mir-6074	0	0	0
hsa-mir-6075	0	0	0
hsa-mir-6076	0	0	0
hsa-mir-6077-1	0	0	0
hsa-mir-6078	0	0	0
hsa-mir-6079	0	0	0
hsa-mir-6080	0	0	0
hsa-mir-6081	0	0	0

hsa-mir-6082	0	0	0
hsa-mir-6083	0	0	0
hsa-mir-6084	0	0	0
hsa-mir-6085	0	0	0
hsa-mir-6086	0	0	0
hsa-mir-6088	0	0	0
hsa-mir-6089-1	0	0	0
hsa-mir-6090	0	0	0
hsa-mir-6124	0	0	0
hsa-mir-6125	0	0	0
hsa-mir-6126	0	0	0
hsa-mir-6127	0	0	0
hsa-mir-6128	0	0	0
hsa-mir-378j	0	0	0
hsa-mir-6129	0	0	0
hsa-mir-6130	0	0	0
hsa-mir-6131	0	0	0
hsa-mir-6132	0	0	0
hsa-mir-6132	0	0	0
hsa-mir-6133	0	0	0
hsa-mir-6134	0	0	0
hsa-mir-6165	0	0	0
hsa-mir-6499	0	0	0
hsa-mir-548ay	0	0	0
hsa-mir-6500	0	0	0
hsa-mir-548az	0	0	0
hsa-mir-6501	0	0	0
hsa-mir-6502	0	0	0
hsa-mir-6503	0	0	0
hsa-mir-6504	0	0	0
hsa-mir-6505	0	0	0
hsa-mir-6506	0	0	0
hsa-mir-6507	0	0	0
hsa-mir-6508	0	0	0
hsa-mir-6509	0	0	0
hsa-mir-6510	0	0	0
hsa-mir-6511a-1	0	0	0
hsa-mir-6512	0	0	0
hsa-mir-6513	0	0	0
hsa-mir-6514	0	0	0
hsa-mir-6515	0	0	0
hsa-mir-6715a	0	0	0
hsa-mir-6715b	0	0	0
hsa-mir-6716	0	0	0
hsa-mir-6717	0	0	0
hsa-mir-6511b-1	0	0	0
hsa-mir-6718	0	0	0
hsa-mir-6719	0	0	0
hsa-mir-6720	0	0	0
hsa-mir-6721	0	0	0
hsa-mir-6722	0	0	0
hsa-mir-6723	0	0	0

hsa-mir-6724	0	0	0
hsa-mir-892c	0	0	0
hsa-mir-892c	0	0	0
hsa-mir-6726	0	0	0
hsa-mir-6727	0	0	0
hsa-mir-6727	0	0	0
hsa-mir-6728	0	0	0
hsa-mir-6729	0	0	0
hsa-mir-6730	0	0	0
hsa-mir-6731	0	0	0
hsa-mir-6732	0	0	0
hsa-mir-6733	0	0	0
hsa-mir-6734	0	0	0
hsa-mir-6735	0	0	0
hsa-mir-6736	0	0	0
hsa-mir-6738	0	0	0
hsa-mir-6739	0	0	0
hsa-mir-6740	0	0	0
hsa-mir-6741	0	0	0
hsa-mir-6742	0	0	0
hsa-mir-6743	0	0	0
hsa-mir-6744	0	0	0
hsa-mir-6745	0	0	0
hsa-mir-6746	0	0	0
hsa-mir-6747	0	0	0
hsa-mir-6748	0	0	0
hsa-mir-6749	0	0	0
hsa-mir-6750	0	0	0
hsa-mir-6751	0	0	0
hsa-mir-6752	0	0	0
hsa-mir-6753	0	0	0
hsa-mir-6754	0	0	0
hsa-mir-6755	0	0	0
hsa-mir-6756	0	0	0
hsa-mir-6757	0	0	0
hsa-mir-6758	0	0	0
hsa-mir-6759	0	0	0
hsa-mir-6760	0	0	0
hsa-mir-6761	0	0	0
hsa-mir-6762	0	0	0
hsa-mir-6763	0	0	0
hsa-mir-6764	0	0	0
hsa-mir-6765	0	0	0
hsa-mir-6766	0	0	0
hsa-mir-6767	0	0	0
hsa-mir-6768	0	0	0
hsa-mir-6769a	0	0	0
hsa-mir-6770-1	0	0	0
hsa-mir-6771	0	0	0
hsa-mir-6772	0	0	0
hsa-mir-6773	0	0	0
hsa-mir-6774	0	0	0

hsa-mir-6775	0	0	0
hsa-mir-6776	0	0	0
hsa-mir-6777	0	0	0
hsa-mir-6778	0	0	0
hsa-mir-6779	0	0	0
hsa-mir-6780a	0	0	0
hsa-mir-6781	0	0	0
hsa-mir-6782	0	0	0
hsa-mir-6783	0	0	0
hsa-mir-6784	0	0	0
hsa-mir-6785	0	0	0
hsa-mir-6786	0	0	0
hsa-mir-6787	0	0	0
hsa-mir-6788	0	0	0
hsa-mir-6789	0	0	0
hsa-mir-6790	0	0	0
hsa-mir-6791	0	0	0
hsa-mir-6792	0	0	0
hsa-mir-6793	0	0	0
hsa-mir-6794	0	0	0
hsa-mir-6795	0	0	0
hsa-mir-6796	0	0	0
hsa-mir-6797	0	0	0
hsa-mir-6798	0	0	0
hsa-mir-6799	0	0	0
hsa-mir-6800	0	0	0
hsa-mir-6800	0	0	0
hsa-mir-6801	0	0	0
hsa-mir-6802	0	0	0
hsa-mir-6803	0	0	0
hsa-mir-6804	0	0	0
hsa-mir-6805	0	0	0
hsa-mir-6806	0	0	0
hsa-mir-6807	0	0	0
hsa-mir-6808	0	0	0
hsa-mir-6809	0	0	0
hsa-mir-6810	0	0	0
hsa-mir-6811	0	0	0
hsa-mir-6812	0	0	0
hsa-mir-6813	0	0	0
hsa-mir-6814	0	0	0
hsa-mir-6815	0	0	0
hsa-mir-6816	0	0	0
hsa-mir-6817	0	0	0
hsa-mir-6818	0	0	0
hsa-mir-6819	0	0	0
hsa-mir-6820	0	0	0
hsa-mir-6821	0	0	0
hsa-mir-6822	0	0	0
hsa-mir-6823	0	0	0
hsa-mir-6824	0	0	0
hsa-mir-6825	0	0	0

hsa-mir-6826	0	0	0
hsa-mir-6827	0	0	0
hsa-mir-6828	0	0	1
hsa-mir-6829	0	0	0
hsa-mir-6830	0	0	0
hsa-mir-6831	0	0	0
hsa-mir-6832	0	0	0
hsa-mir-6833	0	0	0
hsa-mir-6834	0	0	0
hsa-mir-6835	0	0	0
hsa-mir-6780b	0	0	0
hsa-mir-6836	0	0	0
hsa-mir-6837	0	0	0
hsa-mir-6838	0	0	0
hsa-mir-6839	0	0	0
hsa-mir-6840	0	0	0
hsa-mir-6841	0	0	0
hsa-mir-6842	0	0	0
hsa-mir-6843	0	0	0
hsa-mir-6844	0	0	0
hsa-mir-6845	0	0	0
hsa-mir-6846	0	0	0
hsa-mir-6847	0	0	0
hsa-mir-6848	0	0	0
hsa-mir-6849	0	0	0
hsa-mir-6850	0	0	0
hsa-mir-6851	0	0	0
hsa-mir-6852	0	0	0
hsa-mir-6853	0	0	0
hsa-mir-6854	0	0	0
hsa-mir-6855	0	0	0
hsa-mir-6856	0	0	0
hsa-mir-6857	0	0	0
hsa-mir-6858	0	0	0
hsa-mir-6859-1	0	0	0
hsa-mir-6769b	0	0	0
hsa-mir-6860	0	0	0
hsa-mir-6861	0	0	0
hsa-mir-6862-1	0	0	0
hsa-mir-6863	0	0	0
hsa-mir-6864	0	0	0
hsa-mir-6865	0	0	0
hsa-mir-6866	0	0	0
hsa-mir-6867	0	0	0
hsa-mir-6868	0	0	0
hsa-mir-6869	0	0	0
hsa-mir-6870	0	0	0
hsa-mir-6871	0	0	0
hsa-mir-6872	0	0	0
hsa-mir-6873	0	0	0
hsa-mir-6874	0	0	0
hsa-mir-6875	0	0	0

hsa-mir-6876	0	0	0
hsa-mir-6877	0	0	0
hsa-mir-6878	0	0	0
hsa-mir-6879	0	0	0
hsa-mir-6880	0	0	0
hsa-mir-6881	0	0	0
hsa-mir-6882	0	0	0
hsa-mir-6883	0	0	0
hsa-mir-6884	0	0	0
hsa-mir-6885	0	0	0
hsa-mir-6886	0	0	0
hsa-mir-6887	0	0	0
hsa-mir-6888	0	0	0
hsa-mir-6889	0	0	0
hsa-mir-6890	0	0	0
hsa-mir-6891	0	0	0
hsa-mir-6892	0	0	0
hsa-mir-6893	0	0	0
hsa-mir-6894	0	0	0
hsa-mir-6895	0	0	0
hsa-mir-7106	0	0	0
hsa-mir-7107	0	0	0
hsa-mir-7108	0	0	0
hsa-mir-7109	0	0	0
hsa-mir-7110	0	0	0
hsa-mir-7111	0	0	0
hsa-mir-7112-1	0	0	0
hsa-mir-7113	0	0	0
hsa-mir-7114	0	0	0
hsa-mir-6511b-2	0	0	0
hsa-mir-3690-2	0	0	0
hsa-mir-6077-2	0	0	0
hsa-mir-6089-2	0	0	0
hsa-mir-6511a-2	0	0	0
hsa-mir-6511a-3	0	0	0
hsa-mir-6511a-4	0	0	0
hsa-mir-7150	0	0	0
hsa-mir-7151	0	0	0
hsa-mir-7152	0	0	0
hsa-mir-7153	0	0	0
hsa-mir-7154	0	0	0
hsa-mir-7155	0	0	0
hsa-mir-7156	0	0	0
hsa-mir-7157	0	0	0
hsa-mir-7158	0	0	0
hsa-mir-7161	0	0	0
hsa-mir-7159	0	0	0
hsa-mir-7160	0	0	0
hsa-mir-486-2	0	0	0
hsa-mir-486-2	0	0	0
hsa-mir-7162	0	0	0
hsa-mir-7515	0	0	0

hsa-mir-7702	0	0	0
hsa-mir-7703	0	0	0
hsa-mir-7704	0	0	0
hsa-mir-7705	0	0	0
hsa-mir-7706	0	0	0
hsa-mir-7843	0	0	0
hsa-mir-4433b	0	0	0
hsa-mir-1273h	0	0	0
hsa-mir-6516	0	0	0
hsa-mir-7844	0	0	0
hsa-mir-7845	0	0	0
hsa-mir-7846	0	0	0
hsa-mir-7847	0	0	0
hsa-mir-7848	0	0	0
hsa-mir-7849	0	0	0
hsa-mir-7850	0	0	0
hsa-mir-7851	0	0	0
hsa-mir-7852	0	0	0
hsa-mir-7853	0	0	0
hsa-mir-7854	0	0	0
hsa-mir-7855	0	0	0
hsa-mir-7856	0	0	0
hsa-mir-548ba	0	0	0
hsa-mir-548ba	0	0	0
hsa-mir-7973-1	0	0	0
hsa-mir-7973-2	0	0	0
hsa-mir-7973-2	0	0	0
hsa-mir-7974	0	0	0
hsa-mir-7975	0	0	0
hsa-mir-7976	0	0	0
hsa-mir-7977	0	0	0
hsa-mir-7978	0	0	0
hsa-mir-8052	0	0	0
hsa-mir-8053	0	0	0
hsa-mir-8054	0	0	0
hsa-mir-8055	0	0	0
hsa-mir-8056	0	0	0
hsa-mir-8057	0	0	0
hsa-mir-8058	0	0	0
hsa-mir-8059	0	0	0
hsa-mir-8060	0	0	0
hsa-mir-8061	0	0	0
hsa-mir-8062	0	0	0
hsa-mir-8063	0	0	0
hsa-mir-8064	0	0	0
hsa-mir-8065	0	0	0
hsa-mir-8066	0	0	0
hsa-mir-8067	0	0	0
hsa-mir-8068	0	0	0
hsa-mir-8069	0	0	0
hsa-mir-8070	0	0	0
hsa-mir-8070	0	0	0

hsa-mir-8071-1	0	0	0
hsa-mir-8072	0	0	0
hsa-mir-8073	0	0	0
hsa-mir-8074	0	0	0
hsa-mir-8075	0	0	0
hsa-mir-8075	0	0	0
hsa-mir-8076	0	0	0
hsa-mir-8077	0	0	0
hsa-mir-8078	0	0	0
hsa-mir-8079	0	0	0
hsa-mir-8080	0	0	0
hsa-mir-8081	0	0	0
hsa-mir-8082	0	0	0
hsa-mir-8083	0	0	0
hsa-mir-8084	0	0	0
hsa-mir-8085	0	0	0
hsa-mir-8086	0	0	0
hsa-mir-8087	0	0	0
hsa-mir-8088	0	0	0
hsa-mir-8089	0	0	0
hsa-mir-7112-2	0	0	0
hsa-mir-6862-2	0	0	0
hsa-mir-8071-2	0	0	0
hsa-mir-6770-2	0	0	0
hsa-mir-6770-3	0	0	0
hsa-mir-6859-2	0	0	0
hsa-mir-6859-3	0	0	0
SNORA11B	0	0	0
SNORA11C	0	0	0
SNORA11D	0	0	0
SNORA11E	0	0	0
SNORA36C	0	0	0
SNORA38B	0	0	0
SNORA38B	0	0	0
SNORA38B	0	0	0
SNORA84	0	0	0
SNORA84	0	0	0
SNORA84	0	0	0
SNORD119	0	0	0
SNORD121A	0	0	0
SNORD121B	0	0	0
SNORD121B	0	0	0
SNORD123	0	0	0
SNORD124	0	0	0
SNORD125	0	0	0
SNORD126	0	0	0
SNORD127	0	0	0
U100	0	0	0
U101	0	0	0
U102	0	0	0
U103B	0	0	0
U103	0	0	0

U104	0	0	0
U105B	0	0	0
U105	0	0	0
U106	0	0	0
U107	0	0	0
U108	0	0	0
U108	0	0	0
U109	0	0	0
U109	0	0	0
U13	0	0	0
U14A	0	0	0
U14A	0	0	0
U14B	0	0	0
U14B	0	0	0
U15A	0	0	0
U15B	0	0	0
U16	0	0	0
U17b	0	0	0
U18A	0	0	0
U18A	0	0	0
U18B	0	0	0
U18C	0	0	0
U19-2	0	0	0
U19	0	0	0
U20	0	0	0
U21	0	0	0
U23	0	0	0
U23	0	0	0
U24	0	0	0
U25	0	0	0
U26	0	0	0
U27	0	0	0
U27	0	0	0
U28	0	0	0
U28	0	0	0
U29	0	0	0
U30	0	0	0
U31	0	0	0
U31	0	0	0
U32A	0	0	0
U32B	0	0	0
U33	0	0	0
U34	0	0	0
U35A	0	0	0
U35B	0	0	0
U36A	0	0	0
U36B	0	0	0
U36C	0	0	0
U37	0	0	0
U38A	0	0	0
U38B	0	0	0
U38B	0	0	0

U3	0	0	0
U41	0	0	0
U42A	0	0	0
U42B	0	0	0
U42B	0	0	0
U43	0	0	0
U43	0	0	0
U44	0	0	0
U45A	0	0	0
U45A	0	0	0
U45B	0	0	0
U45C	0	0	0
U46	0	0	0
U46	0	0	0
U46	0	0	0
U47	0	0	0
U48	0	0	0
U49A	0	0	0
U49A	0	0	0
U49A	0	0	0
U49B	0	0	0
U49B	0	0	0
U50B	0	0	0
U50B	0	0	0
U50	0	0	0
U51	0	0	0
U52	0	0	0
U53	0	0	0
U54	0	0	0
U55	0	0	0
U56	0	0	0
U56	0	0	0
U57	0	0	0
U58A	0	0	0
U58B	0	0	0
U58C	0	0	0
U58C	0	0	0
U59A	0	0	0
U59B	0	0	0
U60	0	0	0
U61	0	0	0
U62A	0	0	0
U62B	0	0	0
U63	0	0	0
U64	0	0	0
U65	0	0	0
U66	0	0	0
U67	0	0	0
U68	0	0	0
U68	0	0	0
U69	0	0	0
U70B	0	0	0

U70C	0	0	0
U70D	0	0	0
U70D	0	0	0
U70E	0	0	0
U70F	0	0	0
U70F	0	0	0
U70G	0	0	0
U70G	0	0	0
U70	0	0	0
U71a	0	0	0
U71b	0	0	0
U71b	0	0	0
U71c	0	0	0
U71c	0	0	0
U71d	0	0	0
U71d	0	0	0
U72	0	0	0
U73a	0	0	0
U73b	0	0	0
U74	0	0	0
U75	0	0	0
U75	0	0	0
U76	0	0	0
U77	0	0	0
U77	0	0	0
U78	0	0	0
U78	0	0	0
U79	0	0	0
U80	0	0	0
U81	0	0	0
U82	0	0	0
U83A	0	0	0
U83B	0	0	0
U83	0	0	0
U84	0	0	0
U85	0	0	0
U86	0	0	0
U87	0	0	0
U87	0	0	0
U88	0	0	0
U89	0	0	0
U8	0	0	0
U8	0	0	0
U90	0	0	0
U91	0	0	0
U92	0	0	0
U93	0	0	0
U94	0	0	0
U95	0	0	0
U96a	0	0	0
U96b	0	0	0
U97	0	0	0

U98b	0	0	0
U98b	0	0	0
U99	0	0	0
Z17B	0	0	0
hTR	0	0	0
mgU12-22-U4-8	0	0	0
mgU12-22-U4-8	0	0	0
mgU12-22-U4-8	0	0	0
mgU2-19-30	0	0	0
mgU2-19-30	0	0	0
mgU2-25-61	0	0	0
mgU2-25-61	0	0	0
mgU2-25-61	0	0	0
mgU6-47	0	0	0
mgU6-53B	0	0	0
mgU6-53B	0	0	0
mgU6-53	0	0	0
mgU6-53	0	0	0
mgU6-77	0	0	0
mgh18S-121	0	0	0
mgh28S-2409	0	0	0
mgh28S-2411	0	0	0
snR38A	0	0	0
snR38B	0	0	0
snR38C	0	0	0
snR39B	0	0	0
E2	0	0	0
E3	0	0	0
ENSG00000199552	0	0	0
ENSG00000238498	0	0	0
ENSG00000238776	0	0	0
ENSG00000252299	0	0	0
ENSG00000252387	0	0	0
hsa-mir-548n	0	0	0
hsa-mir-548n	0	0	0
hsa-mir-3133	0	0	0
hsa-mir-3613	0	0	0
hsa-mir-548h-5	0	0	0
hsa-mir-4434	0	0	0
hsa-mir-548aj-2	0	0	0
hsa-mir-4466	0	0	0
hsa-mir-548am	0	0	0
hsa-mir-548am	0	0	0
U17a	0	0	0
U17a	0	0	0
U22	0	0	0
U3-2B	0	0	0
U3-2	0	0	0
U3-3	0	0	0
U3-4	0	0	0
AFFX-r2-Ec-c1-bioB-3	0	0	0
AFFX-r2-Ec-c1-bioB-5	0	0	0

AFFX-r2-Ec-c1-bioB-M	0	0	0
AFFX-r2-Ec-c1-bioC-3	0	0	0
AFFX-r2-Ec-c1-bioC-5	0	0	0
AFFX-r2-Ec-c1-bioD-3	0	0	0
AFFX-r2-Ec-c1-bioD-5	0	0	0
AFFX-r2-Ec-c2-bioB-3	0	0	0
AFFX-r2-Ec-c2-bioB-5	0	0	0
AFFX-r2-Ec-c2-bioB-M	0	0	0
AFFX-r2-Ec-c2-bioC-3	0	0	0
AFFX-r2-Ec-c2-bioC-5	0	0	0
AFFX-r2-Ec-c2-bioD-3	0	0	0
AFFX-r2-Ec-c2-bioD-5	0	0	0
AFFX-r2-Ec-c3-bioB-3	0	0	0
AFFX-r2-Ec-c3-bioB-5	0	0	0
AFFX-r2-Ec-c3-bioB-M	0	0	0
AFFX-r2-Ec-c3-bioC-3	0	0	0
AFFX-r2-Ec-c3-bioC-5	0	0	0
AFFX-r2-Ec-c3-bioD-3	0	0	0
AFFX-r2-Ec-c3-bioD-5	0	0	0
AFFX-r2-P1-c1-cre-3	0	0	0
AFFX-r2-P1-c1-cre-5	0	0	0
AFFX-r2-P1-c2-cre-3	0	0	0
AFFX-r2-P1-c2-cre-5	0	0	0
AFFX-r2-P1-c3-cre-3	0	0	0
AFFX-r2-P1-c3-cre-5	0	0	0
spike_in-control-17	0	0	0
spike_in-control-1	0	0	0
spike_in-control-21	0	0	0
spike_in-control-23	0	0	0
spike_in-control-27	0	0	0
spike_in-control-28	0	0	0
spike_in-control-29			
spike_in-control-2	0	0	0
spike_in-control-30	0	0	0
spike_in-control-31	0	0	0
spike_in-control-34	0	0	0
spike_in-control-36	0	0	0
spike_in-control-37	0	0	0
spike_in-control-38	0	0	0
spike_in-control-3	0	0	0
spike_in-control-44	0	0	0
spike_in-control-45	0	0	0
spike_in-control-48	0	0	0
spike_in-control-49	0	0	0
spike_in-control-4	0	0	0
spike_in-control-7	0	0	0
spike_in-control-8	0	0	0
gi:555853	0	0	0
gi:555853	0	0	0
gi:555853	0	0	0
gi:555853	0	0	0
gi:555853	0	0	0

gi:555853	0	0	0
gi:555853	0	0	0
gi:555853	0	0	0
gi:555853	0	0	0
gi:555853	0	0	0
