# Supplementary material for: Novel Broccoli Sulforaphane-Based Analogues Inhibit the Progression of Pancreatic Cancer without Side Effects
Source: Biomolecules. 2020 May 15;10(5):769. doi: 10.3390/biom10050769 (PMC7277136; doi:10.3390/biom10050769)
Supplement: Supplementary file 1 [file biomolecules-10-00769-s001.zip › Biomolecules upload/Suppl_TableS3 Georgikou.pdf]

Table S3 List of differently expressed miRNAs shown in Venn diagrams of Figure 6D

Transcript ID	SF-CO	SF102-SF	SF134-SF
hsa-miR-22-3p	1	0	0
hsa-miR-25-5p	0	1	1
hsa-miR-92a-1-5p	1	0	0
hsa-miR-29b-1-5p	1	1	1
hsa-miR-105-5p	0	0	1
hsa-miR-200b-5p	1	0	0
hsa-miR-23b-5p	1	0	0
hsa-miR-27b-5p	1	1	1
hsa-miR-200a-5p	1	0	0
hsa-miR-525-5p	1	0	0
hsa-miR-2278	1	0	0
hsa-miR-3617-5p	1	0	0
hsa-miR-4521	1	0	1
hsa-miR-4737	1	0	1
hsa-miR-4774-5p	1	0	0
hsa-miR-4784	1	0	0
hsa-miR-5089-5p	0	1	0
hsa-miR-6779-5p	1	0	0
hsa-miR-6892-3p	0	0	1
ENSG00000212377	0	1	0
ENSG00000238791	1	0	0
ENSG00000252543	1	0	0
HBII-240	1	1	0
HBII-52-15	0	1	0
hsa-mir-548g	1	1	0
hsa-mir-4257	0	1	1
hsa-mir-3656	0	0	1
hsa-mir-3689e	0	1	1
hsa-mir-4718	1	0	0
hsa-mir-6828	0	0	1
Sum	20	10	11
SF+102+134	2	2	2
SF+102	2	2	0
SF+134	2	0	2
102+134	0	3	3
**Sum - No. of joined miRs**	**14**	**3**	**4**
