# Supplementary material for: Novel Broccoli Sulforaphane-Based Analogues Inhibit the Progression of Pancreatic Cancer without Side Effects
Source: Biomolecules. 2020 May 15;10(5):769. doi: 10.3390/biom10050769 (PMC7277136; doi:10.3390/biom10050769)
Supplement: Supplementary file 1 [file biomolecules-10-00769-s001.zip › Biomolecules upload/Suppl_TableS4 Georgikou.pdf]

Table S4 Bioinformatic analysis of target genes of the identified miRNA candidates miR2278, miR27b-5p and miR29b-1-5p regarding relation to NF-κB signaling.

hsa-miR	Source	Gene Symbol (NF-κB signaling)
hsa-miR-2278	TargetScan Human and mirDB	CCL21
	TargetScan Human and mirDB	TLR4
	TargetScan Human and mirDB	TRAF1
	TargetScan Human and mirDB	LTA
	TargetScan Human and mirDB	CFLAR
	TargetScan Human and mirDB	BCL2
	TargetScan Human and mirDB	TRAF3
	TargetScan Human and mirDB	TIRAP
	TargetScan Human and mirDB	ERC1
	TargetScan Human and mirDB	PRKCB
	TargetScan Human and mirDB	IL1R1
	TargetScan Human and mirDB	PLCG2
	TargetScan Human and mirDB	XIAP
	TargetScan Human and mirDB	IKBKG
	TargetScan Human and mirDB	TAB1
	TargetScan Human and mirDB	TAB3
	TargetScan Human and mirDB	CSNK2A1
	TargetScan Human and mirDB	CARD10
	TargetScan Human and mirDB	MAR3K7
	TargetScan Human and mirDB	RELA
	TargetScan Human and mirDB	MYD88
	TargetScan Human and mirDB	TRAF6
	TargetScan Human and mirDB	DDX58
hsa-miR-27b-5p	TargetScan Human and mirDB	PLCG1
	TargetScan Human and mirDB	CFLAR
	TargetScan Human and mirDB	TRAF3
	TargetScan Human and mirDB	TRIM25
	TargetScan Human and mirDB	ERC1
	TargetScan Human and mirDB	PRKCB
	TargetScan Human and mirDB	XIAP
	TargetScan Human and mirDB	UBE2I
	TargetScan Human and mirDB	TNFSF14
hsa-miR-29b-1-5p	TargetScan Human and mirDB	PLCG1
	TargetScan Human and mirDB	TRAF3
	TargetScan Human and mirDB	BCL2A1
	TargetScan Human and mirDB	TRIM25
	TargetScan Human and mirDB	TIRAP
	TargetScan Human and mirDB	XIAP
	TargetScan Human and mirDB	DDX58
	TargetScan Human and mirDB	CXCL12
	TargetScan Human and mirDB	MAP3K7
	TargetScan Human and mirDB	TAB2
	TargetScan Human and mirDB	RIPK1
	TargetScan Human and mirDB	ICAM1
