# Supplementary figures and images for: Novel Broccoli Sulforaphane-Based Analogues Inhibit the Progression of Pancreatic Cancer without Side Effects
Source: Biomolecules. 2020 May 15;10(5):769. doi: 10.3390/biom10050769 (PMC7277136; doi:10.3390/biom10050769)

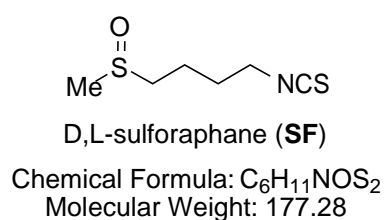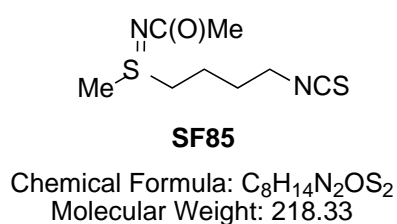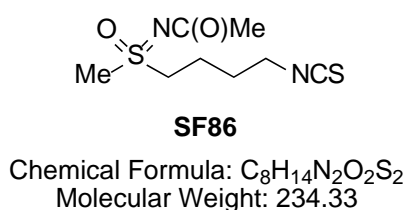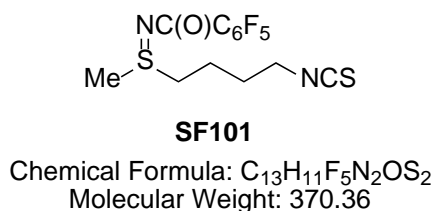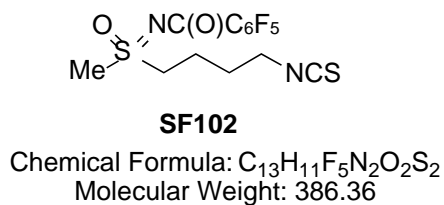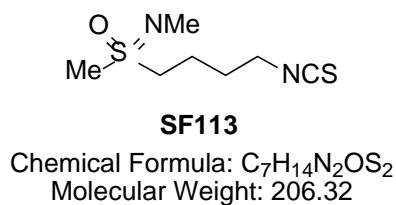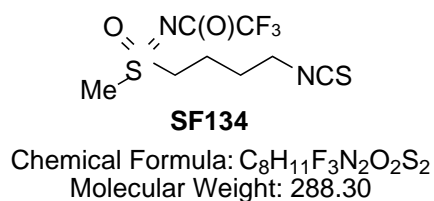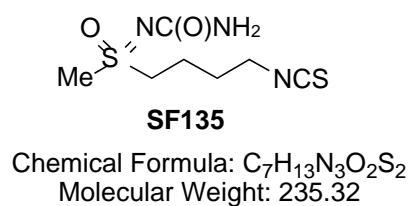

**Figure S1 Schematic representation of sulforaphane derivatives**

Supplement: Supplementary file 1 [file biomolecules-10-00769-s001.zip › Biomolecules upload/Suppl_FigS1 Georgikou.pdf]
